# Promoting Proton Transfer and Stabilizing Intermediates in Catalytic Water Oxidation via Hydrophobic Outer Sphere Interactions

**DOI:** 10.1002/chem.202104562

**Published:** 2022-03-24

**Authors:** Tianqi Liu, Ge Li, Nannan Shen, Linqin Wang, Brian J. J. Timmer, Alexander Kravchenko, Shengyang Zhou, Ying Gao, Yi Yang, Hao Yang, Bo Xu, Biaobiao Zhang, Mårten S. G. Ahlquist, Licheng Sun

**Affiliations:** ^1^ Department of Chemistry School of Engineering Sciences in Chemistry Biotechnology and Health KTH Royal Institute of Technology 10044 Stockholm Sweden; ^2^ Department of Theoretical Chemistry & Biology School of Engineering Sciences in Chemistry Biotechnology and Health KTH Royal Institute of Technology 10691 Stockholm Sweden; ^3^ State Key Laboratory of Radiation Medicine and Protection School for Radiological and Interdisciplinary Sciences (RAD−X) and Collaborative Innovation Center of Radiation Medicine of Jiangsu Higher Education Institutions Soochow University 215123 Suzhou China; ^4^ Center of Artificial Photosynthesis for Solar Fuels School of Science Westlake University 310024 Hangzhou China; ^5^ Nanotechnology and Functional Materials, Department of Materials Sciences and Engineering The Ångström Laboratory Uppsala University 751 03 Uppsala Sweden; ^6^ Wallenberg Wood Science Center Department of Fiber and Polymer Technology KTH Royal Institute of Technology Stockholm 10044 Sweden; ^7^ Institute of Artificial Photosynthesis (IAP) State Key Laboratory of Fine Chemicals Dalian University of Technology (DUT) Dalian 116024 China

**Keywords:** hydrophobicity, intermediates, outer sphere, proton transfer, third coordination sphere, water oxidation

## Abstract

The outer coordination sphere of metalloenzyme often plays an important role in its high catalytic activity, however, this principle is rarely considered in the design of man‐made molecular catalysts. Herein, four Ru‐bda (bda=2,2′‐bipyridine‐6,6′‐dicarboxylate) based molecular water oxidation catalysts with well‐defined outer spheres are designed and synthesized. Experimental and theoretical studies showed that the hydrophobic environment around the Ru center could lead to thermodynamic stabilization of the high‐valent intermediates and kinetic acceleration of the proton transfer process during catalytic water oxidation. By this outer sphere stabilization, a 6‐fold rate increase for water oxidation catalysis has been achieved.

## Graphical abstract

**Outer coordination sphere engineering**: A series of pocket‐shaped catalysts with well‐defined outer coordination environments were synthesized and characterized, showing that the hydrophobic outer coordination sphere could stabilize high‐valent intermediates and promote proton transfer during water oxidation catalysis. The introduction of hydrophobic outer‐sphere interactions is therefore envisaged as an effective strategy to tune other related proton‐coupled electron transfer (PCET) reactions.

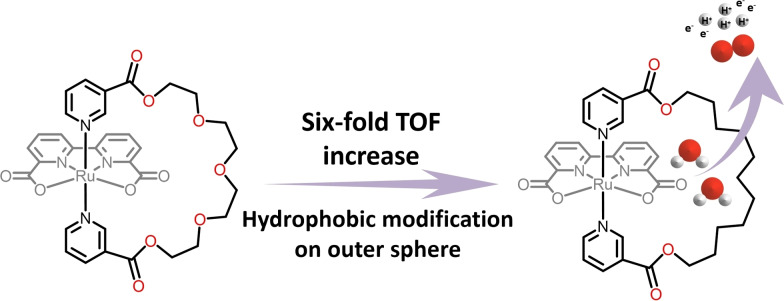

## Introduction

Water splitting into hydrogen and oxygen has received substantial attention as a way to store intermittent electricity in the form of chemical bonds.^[1]^ The overall efficiency of water splitting is usually limited by the sluggish anodic half‐reaction: water oxidation. Therefore, the development of efficient water oxidation catalysts and mechanistic understanding of their functions are highly desirable. Molecular catalysts offer a great platform to investigate the structure‐activity relationship because of the ability to geometrically and electronically tune individual active sites.^[2]^ Chemists have synthesized a library of molecular water oxidation catalysts to mimic the function^[3]^ and structure^[4]^ of the oxygen‐evolving complex (OEC) in photosystem II (PSII) during the past decades, thus a comprehensive understanding of the primary coordination sphere in water oxidation catalysis has been established. In addition to the primary coordination effects, the local chemical environment surrounding OEC also plays a major role in proton and electron transfers in natural photosynthesis. Even the closest structural mimic of the OEC to date displays low activity without mimicking the protein subunits.[^4a^, ^5^] This unfolds into the importance of the outer sphere effect on catalytic water oxidation.

The outer sphere encompasses the solvent and microenvironment in the vicinity of the catalytic site that noncovalently participates in chemical reactions and can influence catalytic activity. Understanding those local chemical environmental factors is a major topic inspiring the design of synthetic catalysts that rival the activity of enzymes.^[6]^ Varieties of microenvironments have been incorporated into molecular water oxidation catalysts, where the interactions between the catalytic site and distal superstructure are mainly composed of hydrogen‐bonding networks and proton transfer‐related functional groups.^[7]^ For example, both introduction of dangling phosphonate,^[8]^ carboxylate,^[9]^ sulfonate^[10]^ group at the second coordination sphere and preorganization of water molecule network^[11]^ near the catalytic site enable remarkable rate enhancements by accelerating the proton transfer process, a concept that also applies to material catalysts.^[12]^ Except for proton transfer, oxygen atom transference to pyridine moieties also has a beneficial effect on the water oxidation or provides alternative pathway for O−O bond formation.^[13]^ The above‐mentioned negatively charged groups also contribute to lower the overpotentials once directly coordinated to the catalytic sites.^[14]^ However, it is challenging to simultaneously incorporate these groups in the inner and outer coordination environments, and complete protonation of more basic groups such as pyridines and more basic oxygenated bases also prevents their wide applications under acidic conditions.^[15]^

Alternatively, the ubiquitous hydrophobic interactions have been shown to significantly influence activities in enzymatic catalysis and molecular devices through stabilizing the intermediates.^[16]^ Meyer found that limited water concentration at nonaqueous solvent could increase water oxidation activity.^[17]^ In addition, hydrophobic interfaces have been proposed to be basic and negatively charged due to the accumulated OH^−^ ions, suggesting that interfacial properties could be used to engineer the proton and electron transfer processes during catalysis.^[18]^ Gounder found that the remarkable rate enhancements of glucose isomerization occurred in hydrophobic zeolite more efficiently than in hydrophilic analogs due to the decreased entropy of the relevant transition states, which was related to hydrogen bonds formed between confined water and glucoses.[^16e^, ^16f^, ^19^] Li also reported that introduction of trifluoromethyl group into the secondary coordination sphere of a Ru‐based polymer could stabilize the charged high‐valent intermediates during water oxidation,^[16d]^ while the lack of well‐defined structure at the molecular level for polymeric materials makes it challenging to draw concrete conclusions about such an outer sphere effect. Accordingly, despite being attractive strategies, control of the hydrophobicity/hydrophilicity of outer spheres is seldom given deliberate consideration in the synthetic design of molecular catalysts to rationally tune activity. Thus, it is necessary to construct catalytic models that feature defined local chemical environments at the molecular level and to study the role of the outer sphere in modulating water oxidation activity.

**Figure 1:**
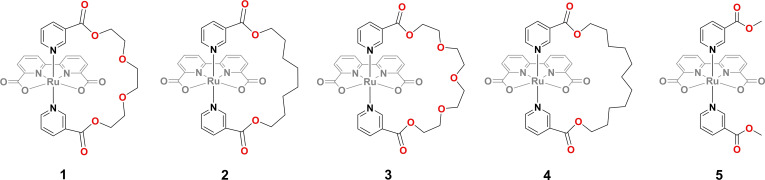
Structures of molecular water oxidation catalysts **1**–**4** and reference catalyst **5**

In this study, four pocket‐shaped water oxidation catalysts (Figure 1) were designed as models of interest in which the hydrophilic/hydrophobic microenvironments provided by the distal ligands could be fine‐tuned.^[20]^ The effect of the outer sphere on the structure‐activity relationship was clarified to provide a better understanding of the modus operandi of water oxidation, i. e., the hydrophobic microenvironment facilitates proton transfer and stabilizes high‐valent intermediates in water oxidation catalysis.

## Results and Discussion

**Synthesis, characterization, and theoretical studies**: Four Ru‐bda‐type catalysts with variable pocket sizes and hydrophilic‐hydrophobic properties were synthesized (Figure 1). Complex **1** has been previously reported to capture the seven‐coordinate aqua ligand at a low oxidation state.^[20]^ The other three pocket‐shape catalysts **2**–**4** and structural Ref. [**5]** ^[21]^ were synthesized following similar procedures (cf. Supporting Information). In short, [Ru(bda)(DMSO)_2_] and the axial ligand were dissolved in methanol, which was refluxed for over 4 h under N_2_. The desired catalysts were isolated via column chromatography and characterized by nuclear magnetic resonance (NMR) and high‐resolution mass spectrometry (HRMS) (Figure S1–S16). Through modification on the linker of pocket ligands, differences in hydrophobicity/hydrophilicity around the catalytic site were maximized while minimizing changes in the primary coordination environment. Therefore, the same intrinsic catalytic activities for complexes **1**–**4** were expected and any observed change in catalytic activity should thus be a result of the altered local microenvironment.

The spin densities and hydrophobicity of the oxygen atom of the Ru^V^(O) were calculated accordingly, which are important for its reactivity to water (Table S2).^[22]^ After incorporating different distal ligands, the hydrophobicity of the oxo in Ru^V^(O) for catalysts **1**–**4** was maintained during the 100 ns molecular dynamics (MD) simulations as shown in Table S3. The average H‐bonds formed around other oxygens were similar to that of classic Ru‐bda‐type catalysts.^[23]^ Similar spin densities and hydrophobicity of the oxo in Ru^V^(O) supported that modifications on outer environments have an insignificant impact on their intrinsic reactivities. The average H‐bonds formed around distal ligands of catalysts **2** and **4** were negligible, which confirmed the hydrophobic nature of aliphatic linkers. In addition, by looking at crystal structures, we previously showed that the distal ligand can affect the surrounding water environment,^[20]^ therefore the influence of hydrophobic ligand on the formed water network near the catalytic sites were investigated. The model of **2** was established based on the crystal structure of **1** at Ru^III^ state^[20]^ by only replacing the O atoms with CH_2_ to minimize the variables. The interactions between Ru and water altered from un‐bonding mode (catalyst **1**) to bonding mode (catalyst **2**) with the distance between approaching water and Ru decreasing from 3.53 Å to 2.66 Å in gas phase and from 3.62 Å to 2.83 Å with PCM solvation model (Figure S42), which indicates that the hydrophobic ligand has the less attractive force to the water molecules of the network and favors the coordination of water molecule. However, the overall influence of distal ligands is tough to evaluate considering the higher degree of freedom of longer distal ligands and the explicit water environment. Catalysts **3** and **4** were not studied here due to the large flexibility of distal ligands as mentioned above.

**Figure 2:**
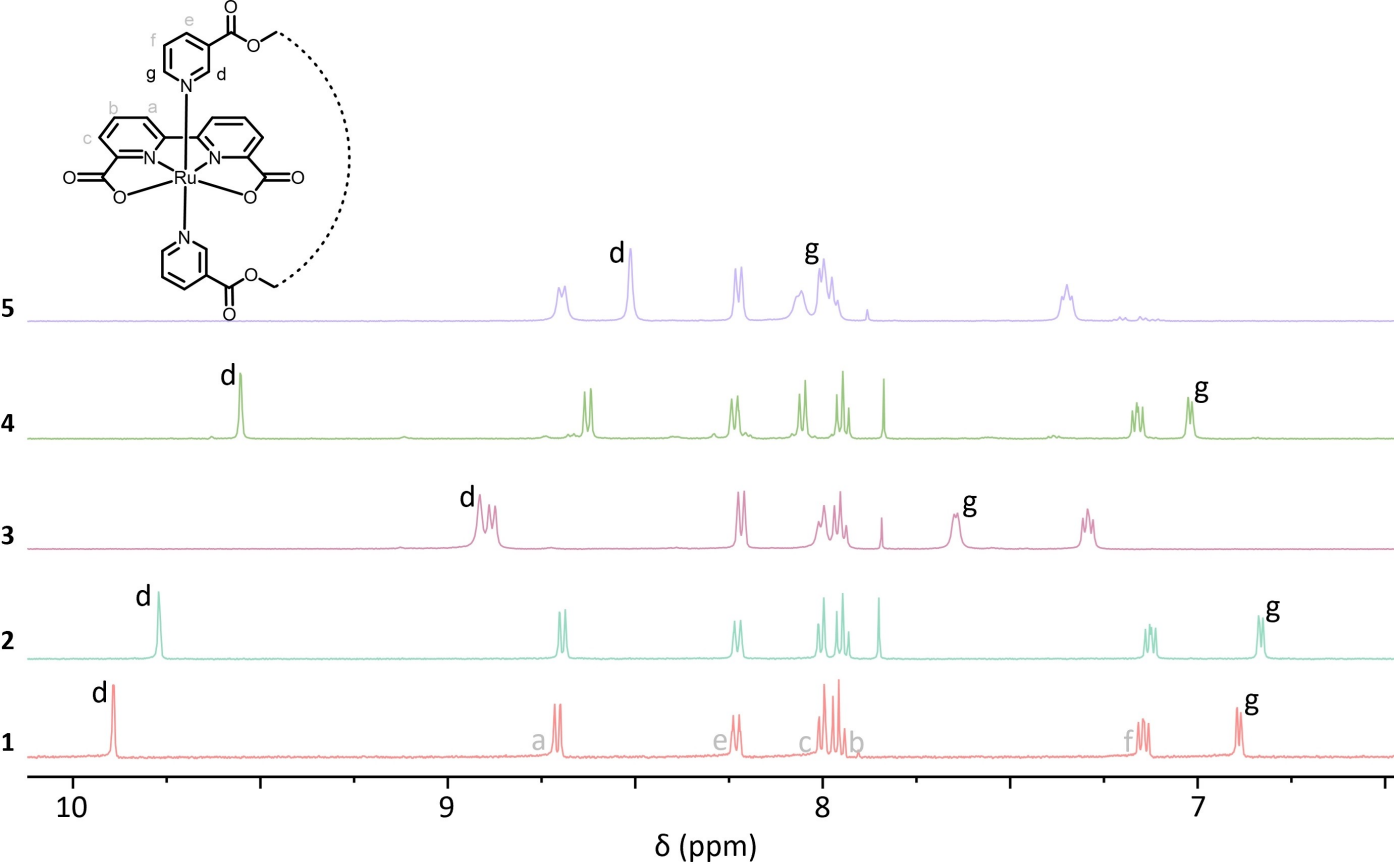
^1^H NMR spectra of complex **1**–**5** in CD_3_OD

The variation in the pocket ligands could have a considerable influence on catalyst conformations. Attaining structural and dynamic information is thus critical for understanding how such designs function in O−O bond formation. ^1^H NMR spectra of complexes **1**–**5** were measured in CD_3_OD, and small amounts of CDCl_3_ were added to improve the solubilities. The previous study showed that the axial ligand of Ref. [5] could rotate freely (flexible conformation), while the small hydrophilic pocket ligand of complex **1** is limited to left‐to‐right switching in front of the catalytic site (locked conformation).^[20]^ In the locked conformation, protons H_g_ and H_d_ are differently affected by the ring current of the bda‐ligand, leading to the downfield shift for H_d_ and upfield shift for H_g_ (Figure 2). Complex **2** and **4** with the hydrophobic pocket ligands exhibited similar tendencies in chemical shifts, therefore the locked conformations could be envisaged. Switching from the long aliphatic to the long glycolic linker in the pocket ligand resulted in the decreased energy differences between the corresponding back‐ and front‐conformation (Figure S17), which suggested complex **3** with the large hydrophilic pocket ligand was much more flexible than others. Experimentally, the smaller chemical shift differences of H_g_ and H_d_ also confirmed that the pocket ligand of **3** was prone to rotate rather freely in solution. In addition, the replacement of the solvent from hydrophilic CD_3_OD to hydrophobic CDCl_3_ maintained the trends in chemical shifts (Figure S18), suggesting that the catalyst conformations do not depend strongly on solvent hydrophobicity.

NMR spectra at various temperatures for complex **4** and Ref. [5] were also measured to validate the proposed locked conformations (Figure S19 and S20). For reference complex **5**, H_g_ gradually shifted upfield and H_d_ shifted downfield as the temperature decreased, finally reaching a spectrum that was similar to that of complex **3** at room temperature. This result suggests that ligand rotation of **5** was somehow limited at lower temperatures. In contrast, the corresponding chemical shifts of complex **4** that already possessed the locked conformation at room temperature were hardly affected by lowering the temperature.

**Figure 3:**
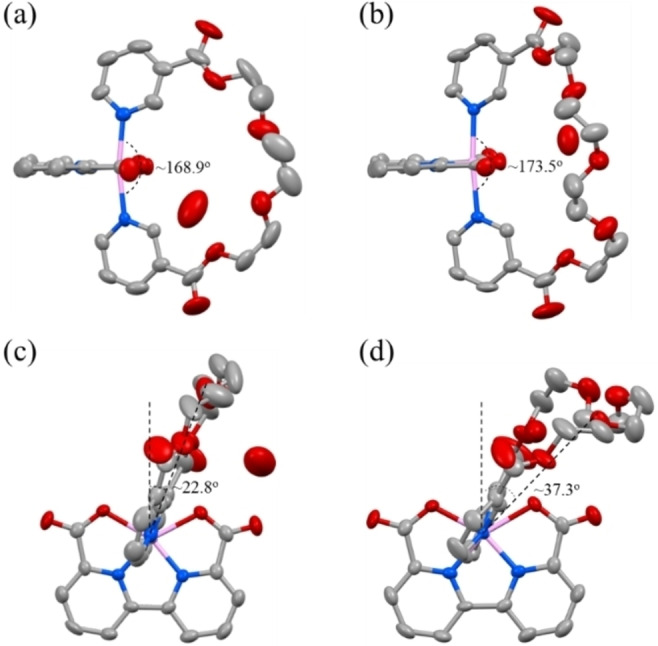
Single crystal structures of complex **1** ⋅ **H_2_O** (**a** and **c**)^[20]^ and **3** ⋅ **H_2_O** (**b** and **d**) with thermal ellipsoids at 50 % probability. Hydrogen atoms are omitted for clarity. Deposition Number 2056826 (for **3** ⋅ **H2O**) contains the supplementary crystallographic data for this paper. These data are provided free of charge by the joint Cambridge Crystallographic Data Centre and Fachinformationszentrum Karlsruhe Access Structures service.

To our delight, the single‐crystal X‐ray structures of **1** ⋅ **H_2_O** and **3** ⋅ **H_2_O** have been successfully obtained serving as models of locked and flexible conformations (Figure 3) of this series catalysts. The Ru centers of **1** ⋅ **H_2_O** and **3** ⋅ **H_2_O** feature distorted octahedral coordination configurations with the N_axial_–Ru–N_axial_ angles of 168.9° and 173.5° respectively, indicating that the longer linker in complex **3** is relatively less rigid than that of in **1**. The pocket ligands of **1** ⋅ **H_2_O** and **3** ⋅ **H_2_O** have rotated away from the vertical axis, giving the offset angles of 22.8° and 37.3° respectively. Both NMR and crystal data support that complex **3** with the larger hydrophilic ligand displays a relatively high rotational flexibility.

**Ce^IV^‐driven water oxidation**: The catalytic performances of water oxidation catalysts **1**–**4** were evaluated using ammonium cerium(IV) nitrate as a sacrificial oxidant in pH 1.0 aqueous solutions. For the catalysts with locked conformation (**1**, **2** and **4**), first‐order relationships between [catalyst] and oxygen evolution rate were obtained, as the presence of distal ligands in front of the catalytic site hinders the radical coupling pathway (I2M, Figure S21 and Table 1). In contrast, for catalyst **3**, in which the distal ligand is rotationally flexible, the reaction order in catalyst concentration is 1.29, suggesting that both mechanisms of water nucleophilic attack (WNA) and I2M are likely to be involved. We previously reported that complex **5** catalyzed water oxidation via I2M pathway with TOF of 166.6 s^−1^ in Ce(IV) solution, where the fast kinetic originates from the off‐set interactions between two asymmetric catalyst units.^[21]^ Therefore, we only compare the performance of catalysts with similar mechanism in the following discussions.

**Table 1:** Catalytic and kinetic data for complexes **1**–**5**.

Catalyst	TOF [s^−1^]^[a]^	Reaction order^[b]^	TOF [s^−1^]^[c]^	E ^III/II^ [V vs. NHE]^[d]^	E ^IV/III^ [V vs. NHE]^[d]^	E ^V/IV^ [V vs. NHE]^[d]^	KIE^[e]^
1	1.3	0.96	0.03	0.73	/	1.18	1.16
2	2.3	0.99	0.09	0.73	0.95	1.14	1.07
3	5.7	1.29	0.28	0.74	0.99	1.17	1.71
4	34.1	1.01	0.31	0.77	0.95	1.14	1.31
5	166.6^[21]^	2^[21]^	0.68	0.77	0.99	1.17	1.66

[a] TOF stands for turnover frequency, values for **1–4** extracted from Ce^IV^‐driven water oxidation activities at pH 1, [cat]=0.16 mM; [b] values of reaction order in catalysts for **1–4** extracted from Ce^IV^‐driven water oxidation activities at pH 1. [c] TOF values extracted from CV according to equation 1; [d] potential measured in 0.1 M NaH_2_PO_4_ aqueous solution with 30 % CF_3_CH_2_OH; [e] KIE stands for kinetic isotope effect, values obtained electrochemically in 0.1 M NaH_2_PO_4_ aqueous solution with 30 % CF_3_CH_2_OH.

**Figure 4:**
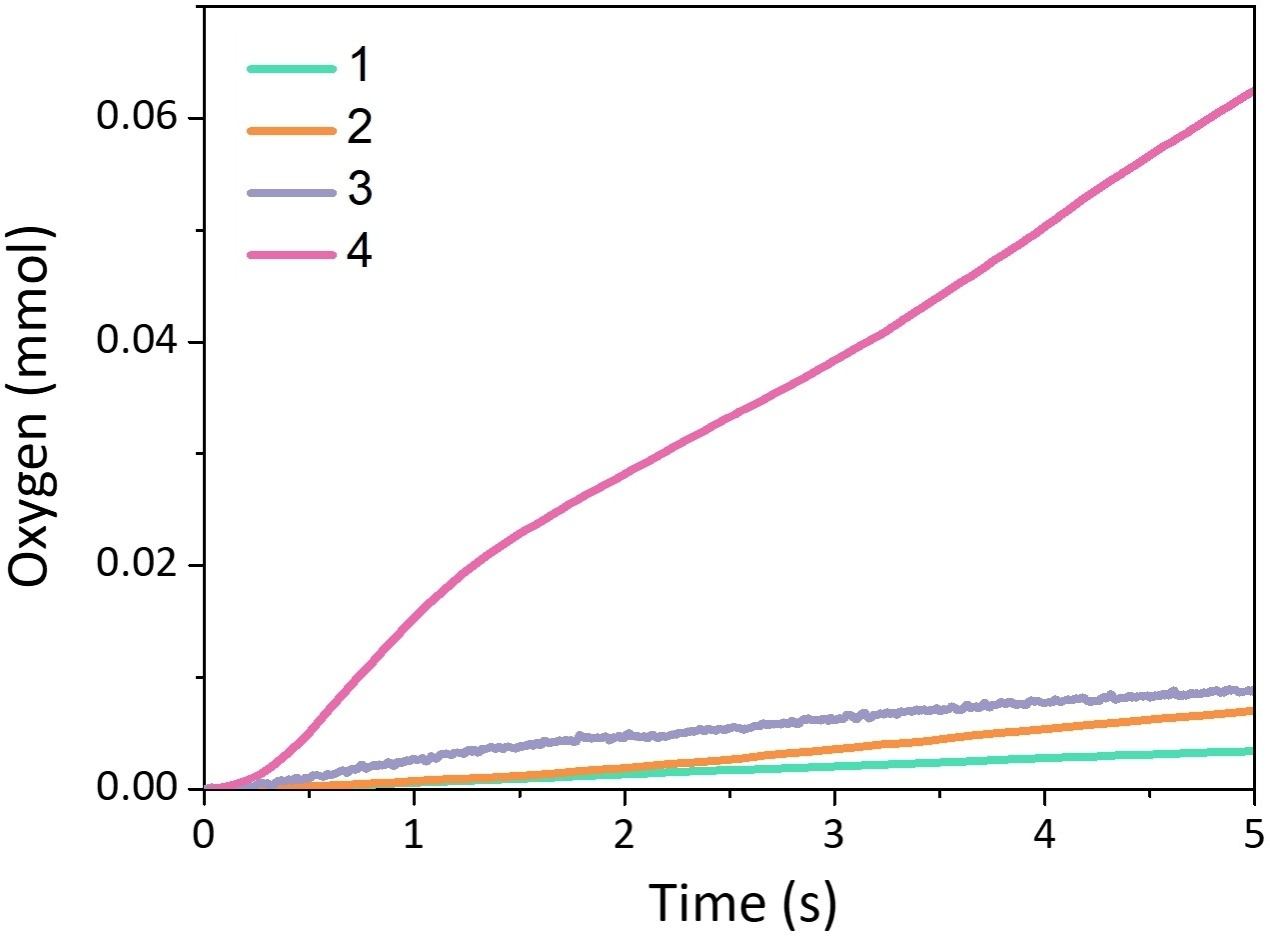
The initial phase of oxygen evolution vs. time for water oxidation catalysts **1**–**4** in 1 : 10 CF_3_CH_2_OH/water (pH 1, acid: trifluoromethane sulfonic acid), [cat]=0.16 mM, [Ce^IV^]=0.12 M.

Catalysts with a hydrophobic outer sphere outperformed their hydrophilic analogs (Figure 4 and Table 1), indicating the promotional effects of hydrophobic outer spheres. Especially, catalyst **4** demonstrated a high catalytic activity with a TOF of 34.1 s^−1^ with a WNA pathway. To the best of our knowledge, this is among the highest activities reported so far for a single‐site Ru‐based catalyst operating via a WNA mechanism in Ce^IV^‐driven water oxidation.[^1^, ^8^] Note that high TOF values exceeding 100 s^−1^ have also been achieved via cooperative water oxidation catalysis in a series of trinuclear ruthenium macrocycles, but the comparable performances per ruthenium are envisaged.[^11^, ^24^] The 6‐fold rate increase of **4** in comparison to its hydrophilic analog **3** also indicated that interfacial properties of the hydrophobic distal ligand appear to contribute to faster water oxidation, however, the changes in steric accessibility of [Ru^V^(O)^+^] to water for WNA cannot be excluded.

Owing to the flexibility of longer distal ligands in the corresponding optimized models, accurate computations of energy barriers via either WNA or I2M are difficult to achieve. Consequently, we only took catalyst **1** as an example to compare its DFT calculated energy barrier via both WNA and I2M pathway to that of classic Ru‐bda‐type catalysts without the distal ligand. The energy profile and transition state structure of the I2M mechanism were displayed in Figure S43 and S44 with a Gibbs free energy barrier of 13.4 kcal/mol. This value is much higher than other Ru‐bda analogs such as [Ru(bda)pic_2_] and [Ru(bda)isoq_2_] (pic=picoline, isoq=isoquinoline), which all have activation energies far below 10.0 kcal/mol.^[25]^ The calculated energy barrier via the WNA mechanism is 18.2 kcal/mol that is also lower than the published values of 26.9 and 32.7 kcal/mol for classic Ru‐bda catalysts.^[26]^ Although the energy barrier of the I2M pathway is still lower than that of WNA for catalyst **1**, the O−O bond formation for the I2M mechanism includes not only activation free energy, but collision frequency and tendency to form the pre‐active Ru^V^(O) dimer before radical coupling.^[27]^ All the contributions could increase the free energy consumption of O−O bond formation for the I2M mechanism. Considering that minor rotation of the distal ligands would cause weak interactions between two catalysts (I2M) or between catalyst and water molecules (WNA), quantitative comparisons of energy barriers among catalysts **1**–**4** are hard to obtain, whereas we believe that the microenvironments around the Ru catalytic site are influenced by altering the properties of distal ligands.

**Stability**: Since catalyst **4** exhibited much higher activity than that of **1**–**3**, we conducted experiments to exclude the possibility of structural evolution of catalyst **4** during catalysis. The HRMS spectra of catalyst **4** were recorded after the addition of 50 eq. of ammonium cerium(IV) nitrate (Figure S22–24). The signal at m/z=742.1603 was observed that is tentatively assigned to the mixed peaks of [C_35_H_36_N_4_O_8_Ru^III^]^+^ and [C_35_H_36_N_4_O_8_Ru^II^+H^+^]^+^. Another peak at m/z=371.5832 is assigned to the doubly charged [C_35_H_36_N_4_O_8_Ru^III^+H^+^]^2+^. We also observed an acetonitrile adduct peak at m/z=392.0966, in which the acetonitrile ligand is likely from the mobile phase of the instrument. Those results suggested the catalyst was stable during catalysis. Besides, we plotted the slopes of the oxygen evolution curve every one second (Figure S25), and similar slopes during the whole catalysis process suggested there was no sudden mechanism switching to I2M.

**Electrochemical studies**: Electrochemical and kinetic studies were performed to diagnose the concrete roles of the hydrophobic outer sphere. Electrocatalytic properties of catalysts **1**–**4** were investigated in 0.1 M NaH_2_PO_4_ aqueous solution with 30 % of CF_3_CH_2_OH. The cyclic voltammograms (CVs) at different scan rates are shown in Figure S26–S35. The diffusion‐controlled electrochemical processes were observed for all catalysts **1**–**4**, evidenced by the linear relationships between peak currents of Ru^III/II^ and the square root of scan rates. According to equation 1 (Supporting Information), the TOF values of catalysts **1**–**4** were calculated to be 0.03, 0.09, 0.28 and 0.31 s^−1^ respectively (Table 1). The relatively high activity of catalysts bearing hydrophobic distal ligands in electrochemical water oxidation thus further supported the promotional effect of hydrophobicity.

**Figure 5:**
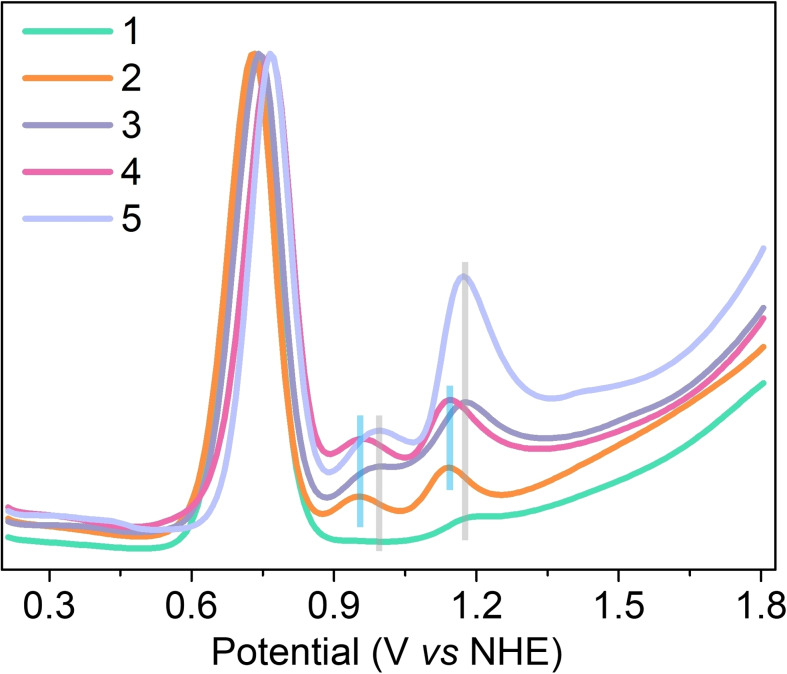
DPVs of complexes **1**–**5** in 0.1 M NaH_2_PO_4_ aqueous solution containing 30 % CF_3_CH_2_OH, [cat]=0.7 mM; The y‐axis was normalized to the same Ru^III/II^ current; grey bars: hydrophilic catalysts, blue bars: hydrophobic catalysts.

**Figure 6:**
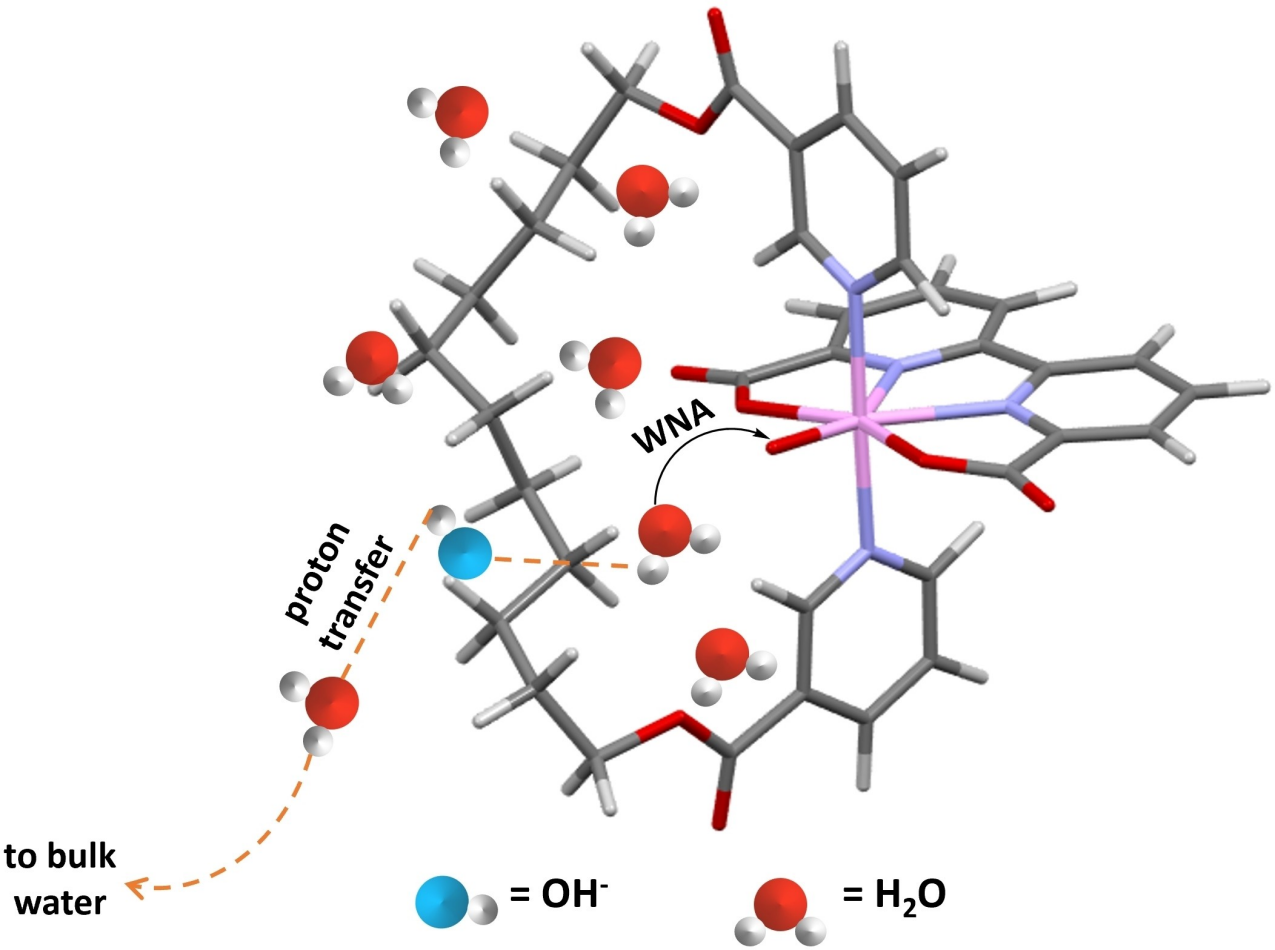
Proposed H‐bonds network around the catalytic site.

The redox potentials were then analyzed to investigate the impact of the hydrophobic microenvironments via differential pulse voltammograms (DPVs, Figure 5), and the corresponding values were summarized in Table 1. The high‐valent intermediates such as [Ru^IV^(OH)^+^] and [Ru^V^(O)^+^] play a vital role in triggering O−O bond formation.^[28]^ Thanks to the hydrophobic microenvironments created by distal ligands, a cathodic shift of 30–40 mV was observed for the Ru^V/IV^ peaks of hydrophobic catalysts, indicating that the hydrophobic microenvironment could stabilize the high‐valent intermediates in some way. Redox potentials for catalysts **1**–**4** have also been calculated by DFT as shown in Table S4. The calculated potentials match well with those measured in the electrochemical experiment. Although the calculated potentials of E^IV/III^ and E^V/IV^ for catalysts **2** and **4** are mostly lower than those for catalysts **1** and **3** respectively, the differences are still minor and within the deviation of the chosen computational methods. Besides, the potentials of Ru^IV/III^ were also shifted negatively. The hydrogen bond network at the hydrophobic microenvironment differs substantially from bulk water, as such the OH^−^ ions are prone to accumulate at the hydrophobic surface.[^18b^, ^18d^] Possibly the basic and negatively charged interface contributes to stabilizing the positively charged [Ru^IV^(OH)^+^] and [Ru^V^(O)^+^] intermediate (Figure 6).

The redox potentials of Ru^III/II^ were good indicators to reflect the electronic properties of axial ligands.^[29]^ As shown in Table 1, the electro‐withdrawing ability of the axial ligands is **4**>**3**>**2**=**1**. Thus, the lower potentials of Ru^IV/III^ and Ru^V/IV^ for catalysts **2** and **4** are unlikely to be caused by electronic effects of axial ligands because electron‐withdrawing ligands would reduce the electron density on the catalytic center and result in a positive shift of potentials.^[30]^

**Kinetic isotope effect**: Kinetic isotope effects (KIEs) are often used to determine the proton transfer kinetics.^[31]^ Therefore, we tested the catalytic performances of complexes **1**–**5** in both water and heavy water to get insights into the proton transfer kinetics (Figure S36–S40). The KIE values were calculated according to equation 2 (cf. Supporting Information) and summarized in Table 1. Contrary to the Ce^IV^‐driven water oxidation, the chemistry near the surface of electrodes would disfavor the radical coupling pathway.^[29a]^ Accordingly, a KIE value of 1.66 was detected for complex **5** that proceeds through an I2M mechanism under Ce^IV^‐driven water oxidation,^[21]^ suggesting a single‐site catalytic behavior (partially) occurred in this case. A similar phenomenon has also been observed by the Meyer group.^[32]^ The single‐site catalytic behaviors in close proximity to the electrodes enable us to compare and analyze proton transfer behaviors for catalysts **1**–**4** without considering differences in the reaction mechanism.

Catalysts **1** and **2** gave small KIE values of 1.16 and 1.07 respectively, suggesting that the corresponding proton and electron transfer processes in rate‐determining steps (RDS) are somehow decoupled.^[33]^ Decoupling the movement of protons and electrons has also been observed in the trinuclear Ru‐bda‐type catalyst with a rigid macrocycle,^[33a]^ which leads to energetic disadvantages and may explain the lower activities over their analogs with larger distal ligands. In addition, the shorter distance between the catalytic site and proton acceptor contributed significantly to the increased reaction rate, which would accordingly result in the decreased KIEs.[^12^, ^34^] As shown in Table 1, the smaller KIEs for hydrophobic catalysts **4** than that of hydrophilic catalysts **3** further supported the hypothesis that the enhanced OH^−^/OD^−^ density at the surface of water‐hydrophobic distal ligands could accelerate the proton transfer during catalysis.

**Base‐assisted water oxidation**: The accumulation of OH^−^ ions around the hydrophobic distal ligand could be further proved by determining the reaction order in additional buffer concentration. When a proton transfer process is involved in the RDS, the concentrated buffer solution (proton acceptor) could decrease the reaction barrier and promote the reaction kinetics. In contrast, contributions from the external buffer solution would be suppressed if the proton acceptors such as carboxylate groups and OH^−^ are preorganized in the vicinity of the catalytic sites.^[12]^ Since decoupled electron transfers are likely involved in the RDS of catalysts **1** and **2**, here catalysts **3** and **4** were used as examples to determine reaction orders in the external buffer's concentration (Figure S41). According to equation 3 in Supporting Information, the reaction orders were calculated to be 0.82 and 0.36 for **3** and **4** respectively, suggesting that the performance of hydrophobic catalysts **4** is less dependent on external proton acceptors. Collectively, KIE and base‐assisted water oxidation results indicated the hydrophobic microenvironments are contributing to the faster proton transfer process.

## Conclusion

This work provides a new viewpoint for tuning the water oxidation activities via hydrophobic outer‐sphere interactions. Experimental evidence shows that the hydrophobic microenvironment could stabilize the [Ru^IV^(OH)^+^] and [Ru^V^(O)^+^] intermediates and promote proton transfer during catalysis. Calculations reproduce the redox potentials with trivial deviations and verified that catalysts **1** and **3** possess more hydrophilic distal ligands while catalysts **2** and **4** own more hydrophobic ones. The spin density distributions and hydrophobicity on the oxo of all catalysts remain unchanged after including distal ligands, thus keeping the intrinsic catalytic property. However, the formed water networks near the catalytic sites are perturbed by changing from hydrophilic to hydrophobic distal ligands, and the less flexibility of distal ligands of catalysts **1**, **2** and **4** render the I2M mechanism less competitive compared to catalyst **3**. A high TOF of 34.1 s^−1^ was obtained for catalyst **4** by the larger hydrophobic distal ligand, which is among the most active catalysts under Ce^IV^‐driven conditions proceeding through a WNA mechanism. The introduction of hydrophobic outer‐sphere interactions is therefore envisaged as an effective strategy to tune other related proton‐coupled electron transfer (PCET) reactions.

## Author Contributions

Tianqi Liu and Licheng Sun conceived the project. Tianqi Liu performed the most of experiments and wrote the manuscript. Ge Li performed the calculation works and wrote the calculation part. Tianqi Liu and Ge Li contributed equally to this work. All authors discussed the results and commented on the manuscript at all stages.

## Conflict of interest

The authors declare no conflict of interest.

1

## Supporting information

As a service to our authors and readers, this journal provides supporting information supplied by the authors. Such materials are peer reviewed and may be re‐organized for online delivery, but are not copy‐edited or typeset. Technical support issues arising from supporting information (other than missing files) should be addressed to the authors.

Supporting Information

## Data Availability

The data that support the findings of this study are available in the supplementary material of this article.
