# Supplementary material for: Promoting Proton Transfer and Stabilizing Intermediates in Catalytic Water Oxidation via Hydrophobic Outer Sphere Interactions
Source: Chemistry. 2022 Mar 24;28(24):e202104562. doi: 10.1002/chem.202104562 (PMC9314586; doi:10.1002/chem.202104562)
Supplement: Supplementary file 1 — Supporting Information [file CHEM-28-0-s001.pdf]

Chemistry–A European Journal

Supporting Information

Promoting Proton Transfer and Stabilizing Intermediates in Catalytic Water Oxidation via Hydrophobic Outer Sphere Interactions

Tianqi Liu, Ge Li, Nannan Shen, Linqin Wang, Brian J. J. Timmer, Alexander Kravchenko, Shengyang Zhou, Ying Gao, Yi Yang, Hao Yang, Bo Xu, Biaobiao Zhang, Mårten S. G. Ahlquist, and Licheng Sun*

General Procedures

All chemicals were purchased from commercial suppliers and used as received. All the solvents were purchased from Fisher Scientific Sweden and used as received. High resolution mass spectrometry was performed at the Dalian University of Technology and Westlake University (UPLC-HRTOF, Synapt-G2-Si, Waters). ^{1}H NMR and ^{13}C NMR spectra of the compounds were recorded with either a Bruker Ascend 400 or a Bruker Avance DMX 500 NMR spectrometer. Electrochemistry measurements were carried out with a CHI660c potentiostat, with glassy carbon as working electrode, Pt wire as auxiliary electrode and measured versus Ag/AgCl/Sat. KCl as reference electrode. All potentials reported herein are converted to their corresponding values versus NHE. Kinetics studies were carried out with an Omega PXM409 pressure transducer. Typically, different amounts of catalyst solutions were injected into a 22.8 mL of sealed pressure tube equipped with a GC septum connected pressure transducer, containing Ce^{IV} solution. After injection, the total liquid volume was 3.1 mL with Ce^{IV} concentrations of 0.12 M. Full consumption of the Ce^{IV} could be observed by eye for all catalysts, and full conversion into the desired product O_2 was checked and confirmed by gas chromatography (GC2014, Shimadzu). TOF values were calculated according to the oxygen evolution rate at the 5-20 s time scale for catalysts **1**-**3,** and 1-4 s time scale for catalyst **4**. Note that we used the same amount of Ce^{IV} to drive the oxygen evolution reactions for all catalysts, which means the TON observed is CAN-limited and cannot represent the stabilities in a longer time scale.

TOF values calculation from electrochemical experiments

Randles–Ševčík equation describes the effect of scan rate on the peak current (i_p) for reversible electrochemical couples.

$$i_p = 0.4463\, n_p F A [cat] \sqrt{\frac{n_p F v D}{RT}}$$

where $\boldsymbol{n_p}$ is number of electrons transferred in the redox event ($\boldsymbol{n_p}$ = 1 for the $Ru^{III/II}$ redox couple), ***F*** is Faraday constant in C mol^{-1}, ***A*** is electrode area in cm^2, **[*cat*]** is the catalyst concentration in mol cm^{-3}, ***v*** is scan rate in V/s, ***D*** is diffusion coefficient in cm^2 s^{-1}, ***R*** is gas constant in J K^{-1} mol^{-1} and ***T*** is temperature in K.

At the steady-state experimental conditions, the scan-rate is independent on the catalytic current (i_{cat}). In this case, i_{cat} could be expressed by following equation

$$i_{cat} = n_{cat}FA[cat]\sqrt{Dk_{cat}}$$

where $\boldsymbol{n_{cat}}$ is 4 for water oxidation reaction.

Then the TOF value could be calculated according to **equation (1)**:

$$\frac{i_{cat}}{i_p} = 1.437\sqrt{\frac{k_{cat}}{v}}$$

KIE values calculation from electrochemical experiments

Based on above equations, kinetic deuterium isotope effect was defined as **equation (2)**:

$$KIE = \frac{k_{cat,H_2O}}{k_{cat,D_2O}} = \frac{\left({i_{cat}}/{i_p}\right)^2_{H_2O}}{\left({i_{cat}}/{i_p}\right)^2_{D_2O}}$$

I_{cat} used in this work was measured at 1.8 V vs NHE.

Determination of the reaction order for buffer

equation (3):

$$\rho_{(buffer)} = \frac{\partial \ln k_{cat}}{\partial \ln(buffer)} = \frac{\partial \ln\left({i_{cat}}/{i_p}\right)^2}{\partial \ln(buffer)}$$

Computational details:

Catalysts **1-4** at different oxidation states were optimized by density function theory (DFT) using the Jaguar 10.3 program package by Schrödinger, LLC.(Int. J. Quantum Chem. 2013, 113 (18), 2110-2142.) The functional and basis set for conformational optimizations were B3LYP-D3[x-x] (J. Chem. Phys. 1993, 98 (7), 5648-6. Phys. Rev. B 1988, 37 (2), 785. J. Chem. Phys. 2010, 132 (15), 154104. Phys. Chem. Chem. Phys. 2011, 13 (14), 6670-6688.) and LACVP**(J. Chem. Phys., 1985, 82, 299-310), respectively. Coordinates of the optimized structures of catalysts **1-4** at II, III, IV, V oxidation states are provided in Supporting Information. Frequency calculations with LACVP** were performed on optimized or TS geometries of catalysts **1-4** to verify that the geometries correspond to the energetic minima or first-order saddle points and obtain the thermochemical properties. Based on the optimized or TS geometries, single-point calculations with a larger basis set LACV3P**++(The LACV3P basis set is a triple-zeta contraction

of the LACVP basis set developed and tested at Schrödinger, Inc.) were performed. The solvation energies were estimated by adapting the Poisson Boltzmann Finite element method (PBF) (J. Am. Chem. Soc. 1994, 116 (26), 11875-11882. J. Phys. Chem. 1996, 100 (28), 11775-11788.) implemented in Jaguar 10.3. The Gibbs free energies are calculated as the following equation G = E(B3LYP-D3/LACV3P^{**++}) + G_{solv} + ZPE + ΔH_{298} – T*ΔS_{298} + 1.9 kcal/mol (a concentration correction to the solvation free energy when changing from 1 M(g) to 1 M(aq)). Gibbs free energies of the low spin states of all species have been compared to those in higher spin states, and all species are most stable in their low spin states. Structures of catalyst **1** and **2** of Ru^{III} complex with three water molecules were optimized with the Gaussian 16 program suite both in gas phase and with Polarizable Continuum Model (PCM) solvation model. (Gaussian 16 Rev. C.01, Wallingford, CT, 2016. Chem. Rev. 2005, 105, 8, 2999–3094. Coordination chemistry reviews. 2017, 346, 206-215.)

After obtaining the Gibbs free energy of every sub-reaction, the standard oxidative potentials were calculated based on the Nernst equation under standard conditions (at 1 atm and 25℃):

$$E^0 = -\frac{\Delta G^0}{nF}$$

where ΔG^0 is the reactive Gibbs free energy under standard conditions; n is the number of electrons transferred in each sub-reaction; F is the Faraday constant, 96485 C/mol; E^0 is the standard oxidative potentials for the corresponding reaction. Standard hydrogen electrode (SHE) is used as the reference with an absolute electrode potential of 4.28 V. (J. Phys. Chem. A. 1998, 102 (40), 7787-7794.) The standard Gibbs free energy of proton in aqueous solution (1M) used is -270.28 kcal/mol. (J. Phys. Chem. A. 1998, 102 (40), 7787-7794.) The Gibbs free energy of proton was then calibrated by the following equation according to the pH value (pH=4.7) in the experiment:

$$G = G^0 - 2.303RT * pH$$

After geometry optimizations at the DFT level a procedure used in our recent publication has been used to prepare topology files of catalysts **1-4** for molecular dynamics (MD) simulations. (Angew. Chem. Int. Ed., 2017, 56, 6962-6965) Force field parameters of the Ru^V(O)bda

fragment for the new complexes were adopted from previous work (Angew. Chem. Int. Ed., 2017, 56, 6962-6965). The bonded and van der Waals parameters for different cyclic ligands were complemented with standard OPLS-AA (all atom optimized molecular potential for liquid simulation) parameters. (J. Am. Chem. Soc., 1996, 118(45): 11225-11236) Atomic partial charge parameters were computed in an iterative way to obtain average electrostatic potential (ESP) charges by taking 20 conformations from short MD calculations with an initial charge distribution. By calculating the ESP charges of the different conformations and taking the average of those charges we reassigned the atomic charges to the new force field.

MD calculations were conducted with GROMACS 2019.6 software package. (SoftwareX 2015, 1-2, 19-25.) A cubic box of an initial size of 5*5*5 nm^3 was used to accommodate $Ru^V(O)$ complexes of catalysts **1-4**, a chloride ion to neutralize the system and SPC/E (J. Chem. Phys., 1983, 79(2): 926-935.) water molecules. Firstly, energy minimization of 100000 steps was done with the Verlet (Comput. Phys. Comm., 2013, 184(12): 2641-2650.) cutoff scheme and Particle Mesh Ewald (J. Chem. Phys., 1995, 103(19): 8577-8593.) method employed to acquire accurate evaluation of the long-range electrostatic interactions beyond the cutoff. Secondly, 100 ps simulation in the canonical NVT ensemble was performed to heat the system to 300 K with v-rescale (J. Chem. Phys., 2007, 126(1): 014101.) thermostat. Thereafter, 100 ps simulation in the isothermal isobaric NPT ensemble was conducted with 1 bar pressure, v-rescale thermostat and Parrinello-Rahman (J. Appl. Phys., 1981, 52(12): 7182-7190.) barostat. Lastly, 100ns calculation in the NPT ensemble was done for further analyses. During the MD calculations in the NVT and NPT ensemble, the Linear Constraint Solver (LINCS) (J. Comput. Chem., 1997, 18(12): 1463-1472.) algorithm was used to constrain all the bond lengths. For the whole simulations, 1fs timestep was used. Analyses of H-bonds were accomplished by Visual Molecular Dynamics (VMD) (J. Mol. Graph., 1996, 14(1): 33-38.).

Single-crystal growth and measurement:

Single crystal of **3·H_2O** was obtained by slow diffusion of diethyl ether into a methanolic solution of **3** at room temperature. The diffraction data of **3** was measured at 293 K by using Mo Kα radiation (λ = 0.71073 Å) on a Bruker D8 Venture single crystal X-ray diffractometer equipped with a kappa geometry goniometer. The dataset was reduced, and absorption

correction was applied in APEX3 suite. The crystal structure was solved by direct methods and refined by full-matrix least-squares on F^2 using the SHELX-2018 program package (Sheldrick, G. M. SHELXT-integrated space-group and crystal-structure determination. Acta Crystallogr. 2015, A 71, 3–8.). All non-hydrogen atoms were refined anisotropically defined and hydrogen atoms were placed in calculated positions by means of the “riding” model. A summary of the crystallographic data, the data collection parameters, and the refinement parameters are given in Table S1.

Table S1 Summary of the crystallographic data for **3·H$_2$O**

	**3·H$_2$O** (CCDC NO. 2056826)
Empirical formula	$C_{32}H_{32}N_4O_{12}Ru$
Formula weight	765.68
Crystal system	Orthorhombic
Space group	$P2_12_12_1$
a/Å	8.8429(8)
b/Å	17.6798(17)
c/Å	20.449(2)
V/Å^3	3197.0(5)
Z	4
T/K	293
λ/Å	0.71073
F(000)	1568
ρ_{calcd}/g cm^{-3}	1.591
μ/mm^{-1}	0.563
Measured refls.	97923
Independent refls.	7114
No. of parameters	424
R_{int}	0.1176
^{a}R indices [$I > 2\sigma(I)$]; R_1, wR_2	0.0471, 0.0750
GOF	1.063

^{a} $R_1 = \sum \| |F_o| - |F_c| \| / \sum |F_o|$, $wR_2 = [\sum w(F_o^2 - F_c^2)^2 / \sum w(F_o^2)^2]^{1/2}$

Synthesis:

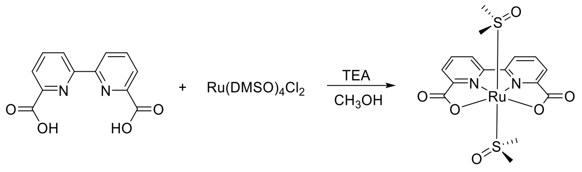

Ru(bda)(DMSO)$_2$

A mixture of 2,2′-bipyridine-6,6′-dicarboxylic acid (H2bda) (2.44 g, 10 mmol), $Ru(DMSO)_4Cl_2$ (4.84 g, 10 mmol) and 8 mL of triethylamine in methanol (200 mL) was heated at 80 °C over 4 hours under N_2. The product was obtained as a brown powder after filtration and washing with methanol, 3.4 g (yield: 68 %). ^{1}H NMR (400 MHz, DMSO-d$_6$) δ 8.65 (d, J = 7.6 Hz, 2H), 8.16 (t, J = 7.9 Hz, 2H), 8.04 (d, J = 7.6 Hz, 2H), 2.54 (s, 12H).

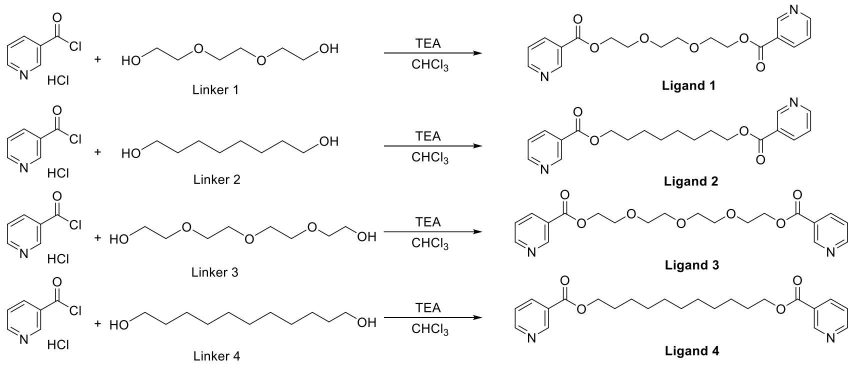

Nicotinoyl chloride hydrochloride (1 g, 5.62 mmol), triethylene glycol (0.42 g, 2.81 mmol) and 2.5 mL of triethylamine were dissolved in 25 mL of chloroform. After stirring for 6 h at room temperature the reaction mixture was then washed with water (20 mL) three times, dried over sodium sulfate, filtered and concentrated in vacuo. The crude product was purified by column chromatography (SiO_2, dichloromethane/methanol 20:1) to afford 0.91 g of **Ligand 1** (yield: 90%) as yellowish oil. ^{1}H NMR (400 MHz, $CDCl_3$) δ 9.23 (s, 2H), 8.77 (d, J = 3.3 Hz 2H), 8.29 (d, J = 7.9 Hz, 2H), 7.44 – 7.32 (m, 2H), 4.49 (m, 4H), 3.84 (m, 4H), 3.72 (s, 4H). **Ligand 2**-**4** were synthesized following a similar procedure. **Ligand 2**: ^{1}H NMR (500 MHz, CD_3OD) δ 9.10 (s, 2H), 8.73 (d, J = 4.9, 2H), 8.37 (t, J = 8.0, 2H), 7.55 (dd, J = 7.7, 5.2 Hz, 2H), 4.36 (t, J = 6.6 Hz, 4H), 1.85 – 1.75 (m, 4H), 1.53 – 1.38 (m, 8H). **Ligand 3**: ^{1}H NMR (500 MHz, DMSO-*d*6) δ 9.08 (s, 2H), 8.82 (d, J = 4.8 Hz, 2H), 8.28 (d, J = f8.0 Hz, 2H), 7.57 (dd, J = 7.9, 4.9 Hz, 2H), 4.44 – 4.36 (m, 4H), 3.77 – 3.70 (m, 4H), 3.56 (m, 8H). **Ligand 4**: ^{1}H NMR (500 MHz, CD_3OD) δ 9.13 (s, 2H), 8.74 (dd, J = 4.9, 1.7 Hz, 2H), 8.39 (dt, J = 8.0, 1.9 Hz, 2H), 7.57 (dd, J = 8.0, 5.0 Hz, 2H), 4.37 (t, J = 6.6 Hz, 4H), 1.82 – 1.75 (m, 4H), 1.50 – 1.28 (m, 14H).

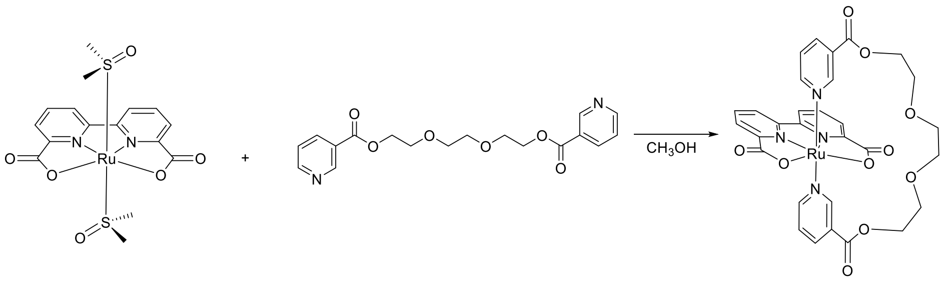

A mixture of Ru(bda)(DMSO)$_2$ (500 mg, 1.0 mmol) and **Ligand 1** (360 mg, 1.0 mmol) in methanol (40 mL) was refluxed over 4 hours under N_2. The crude product was purified by column chromatography (SiO_2, dichloromethane/methanol 10:1) to afford 155 mg of **1** (yield: 22%) as red powder. ^{1}H NMR (500 MHz, CD_3OD) δ 9.89 (d, J = 1.9 Hz, 2H), 8.70 (dd, J = 7.9, 1.3 Hz, 2H), 8.23 (dt, J = 8.0, 1.6 Hz, 2H), 8.01 – 7.93 (m, 4H), 7.14 (dd, J = 8.0, 5.6 Hz, 2H), 6.88 (dd, J = 5.6, 1.4 Hz, 2H), 4.56 – 4.50 (m, 4H), 4.11 (s, 4H), 3.99 – 3.95 (m, 4H). ^{13}C NMR (101 MHz, CD_3OD) δ 173.70, 163.22, 159.44, 157.18, 156.26, 152.25, 137.18, 132.02, 127.19, 125.75, 125.06, 124.97, 71.87, 68.71, 66.60. HR-MS (calcd for $C_{30}H_{27}N_4O_{10}Ru(II)^+$ [**1** + H^+]$^+$: 705.0779; found: 705.0776).

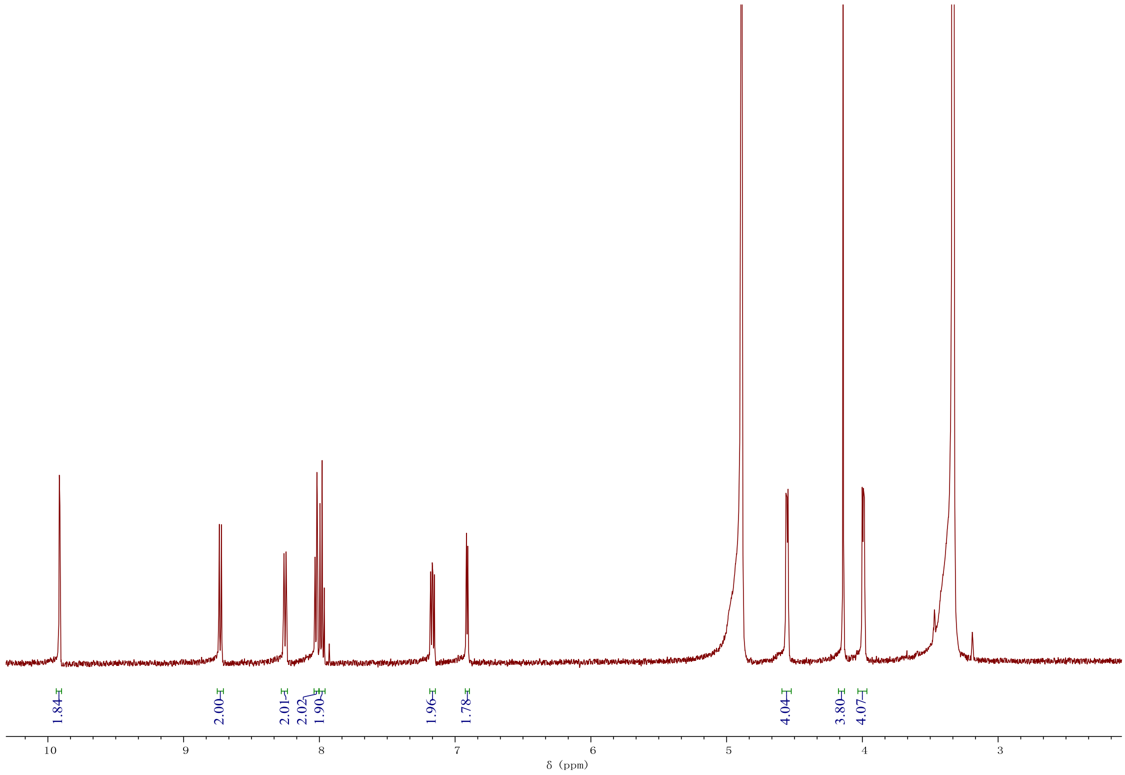

Figure S1 ^{1}H spectrum of **1** in CD_3OD

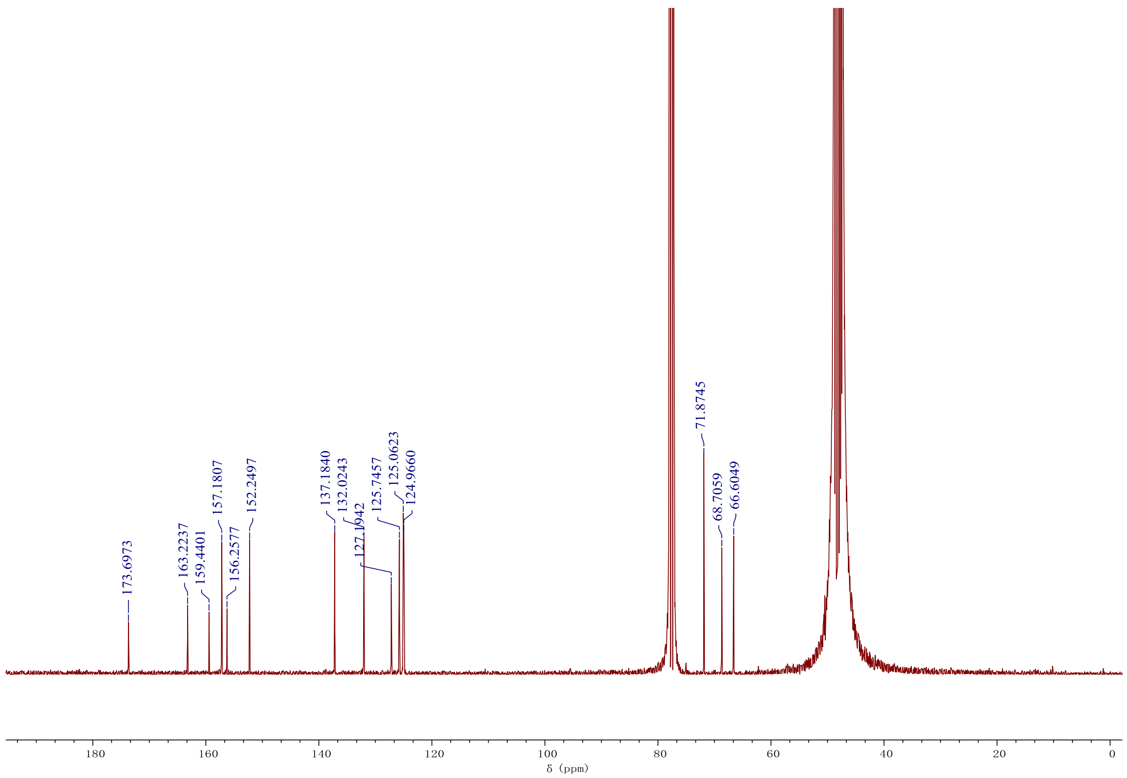

Figure S2 ^{13}C NMR spectrum of **1** in CD_3OD

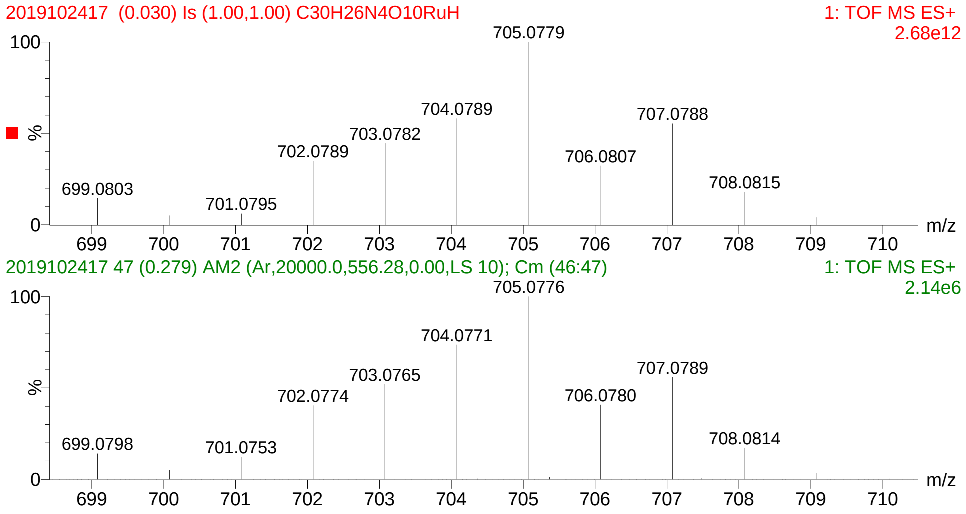

Figure S3 HRMS of **1** (lower) and calculated mass spectrum (upper).

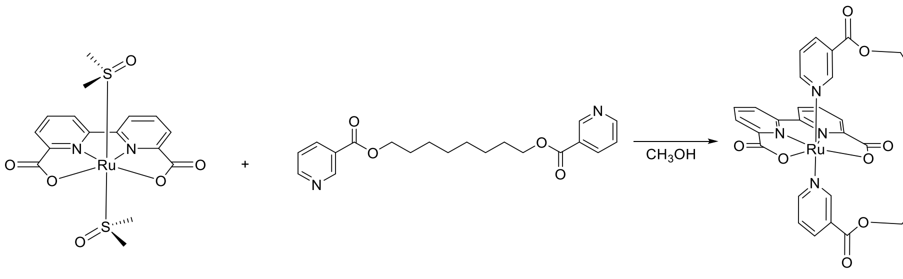

Ru(bda)(DMSO)$_2$ L2 2

2 was synthesized following a similar procedure as reported here for **1** with yield of 23%. ^{1}H NMR (500 MHz, CD_3OD) δ 9.68 (s, 2H), 8.59 (d, J = 7.9 Hz, 2H), 8.13 (d, J = 7.9 Hz, 2H), 7.91 (d, J = 7.3 Hz, 2H), 7.85 (t, J = 7.9 Hz, 2H), 7.03 (dd, J = 7.7, 5.8 Hz, 2H), 6.73 (d, J = 5.1 Hz, 2H), 4.34 (s, 4H), 1.76 (s, 8H), 1.55 (s, 4H). The extra peaks a 0.88 and 1.29 ppm can be assigned to the grease from the column. Other small peaks may be from the solvent, but we cannot assign them at the moment.^{13}C NMR (126 MHz, CD_3OD) δ 175.26, 165.03, 161.04, 157.94, 157.55, 153.51, 138.83, 133.56, 129.05, 127.16, 126.69, 126.51, 68.24, 31.27, 30.57, 28.06. HR-MS (calcd for $C_{32}H_{31}N_4O_8Ru(II)^+$ [**2** + H^+]$^+$: 701.1194; found: 701.1197).

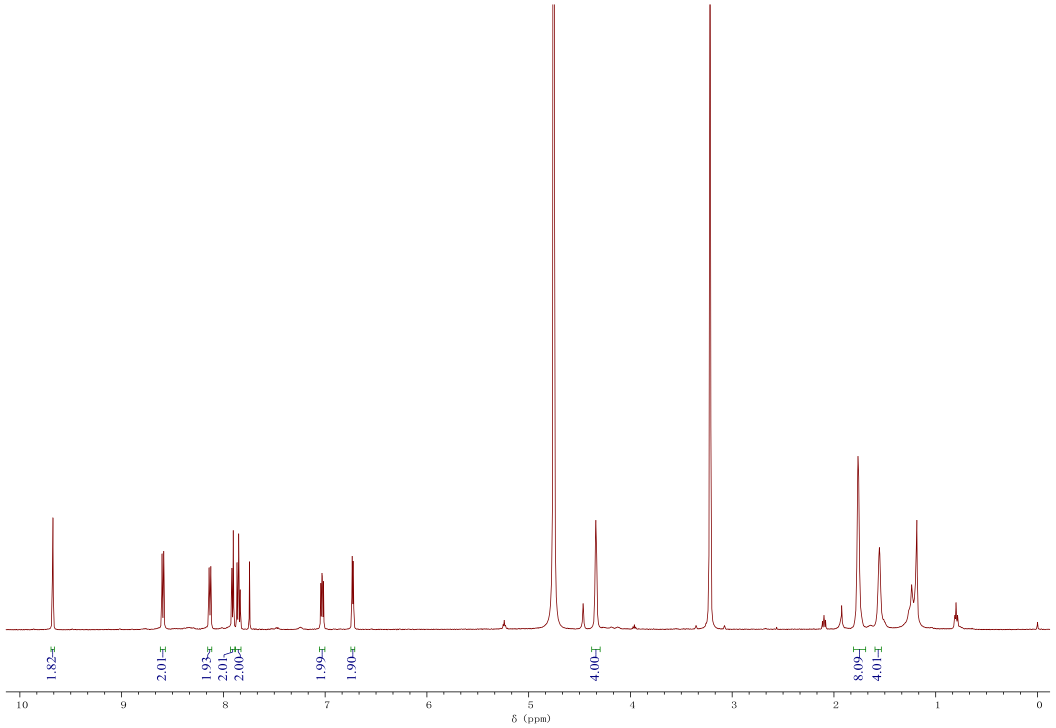

Figure S4 ^{1}H spectrum of **2** in CD_3OD

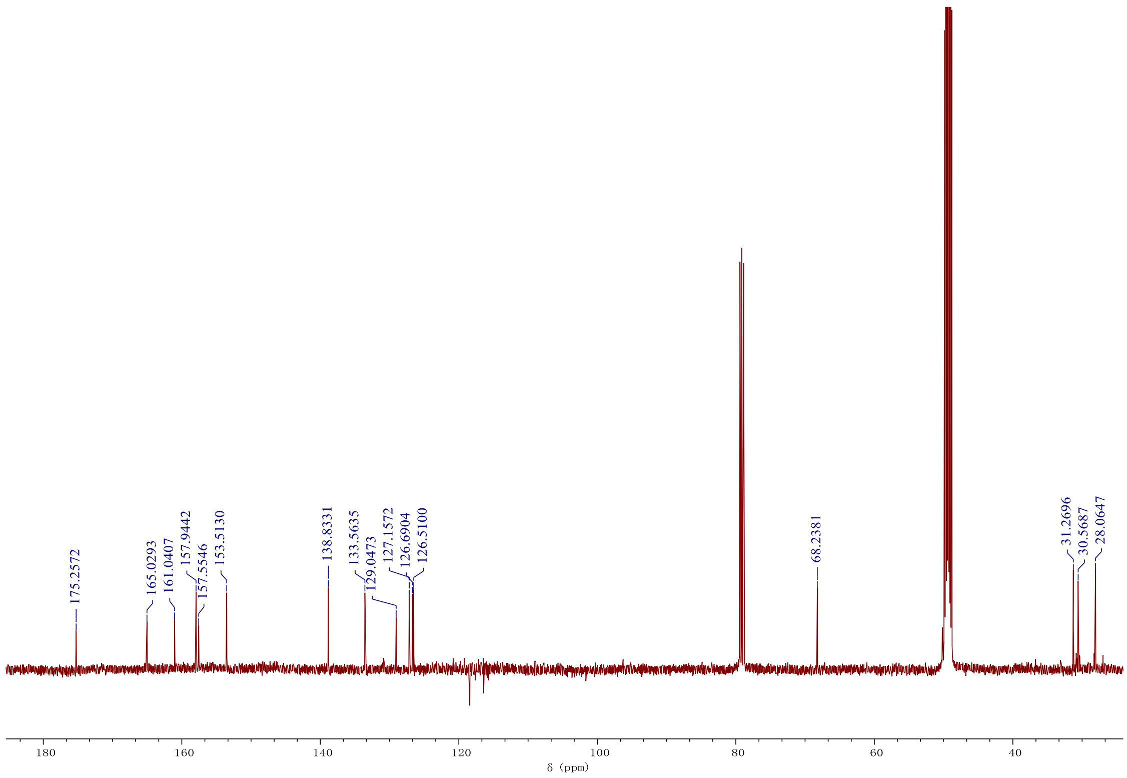

Figure S5 ^{13}C spectrum of **2** in CD_3OD

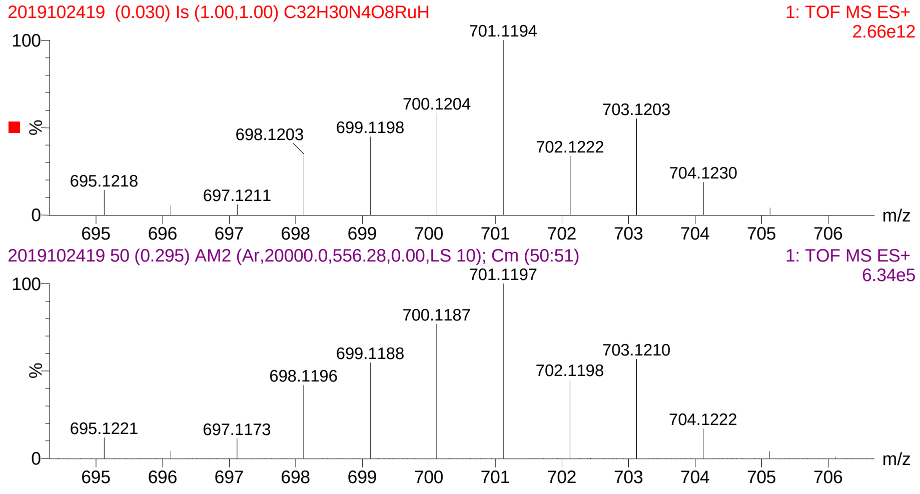

Figure S6 HRMS of **2** (lower) and calculated mass spectrum (upper).

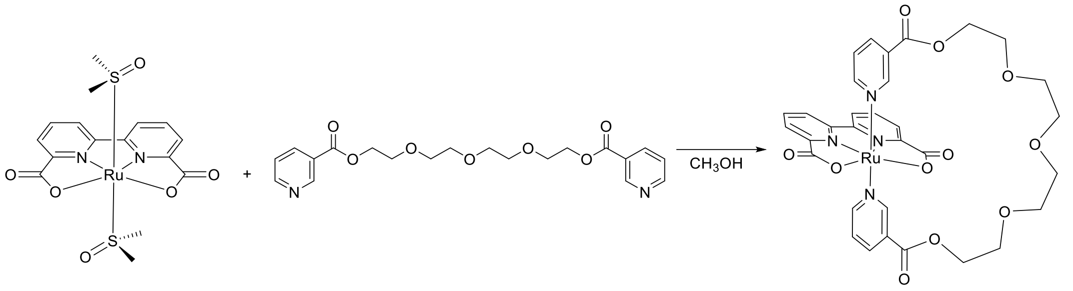

3 was synthesized following a similar procedure as reported here for **1** with yield of 23%. ^{1}H NMR (500 MHz, CD_3OD) δ 8.94 (s, 2H), 8.90 (d, J = 7.9 Hz, 2H), 8.24 (d, J = 7.9 Hz, 2H), 8.03 (d, J = 7.3 Hz, 2H), 7.98 (t, J = 7.8 Hz, 2H), 7.67 (d, J = 4.8 Hz, 2H), 7.35 – 7.28 (m, 2H), 4.48 – 4.41 (m, 4H), 3.93 – 3.99 (m, 8H), 3.89 – 3.83 (m, 4H). ^{13}C NMR (126 MHz, CD_3OD) δ 175.01, 164.46, 161.45, 157.51, 156.22, 154.94, 138.86, 133.84, 128.86, 127.61, 127.18, 126.48, 73.03, 72.56, 70.61, 67.16. HR-MS (calcd for $C_{32}H_{31}N_4O_{11}Ru(II)^+$ [**3** + H^+]$^+$: 749.1042; found: 749.1038; $C_{32}H_{30}N_4O_{11}NaRu$ (II)$^+$ [**3** + Na^+]$^+$ found: 771.0856).

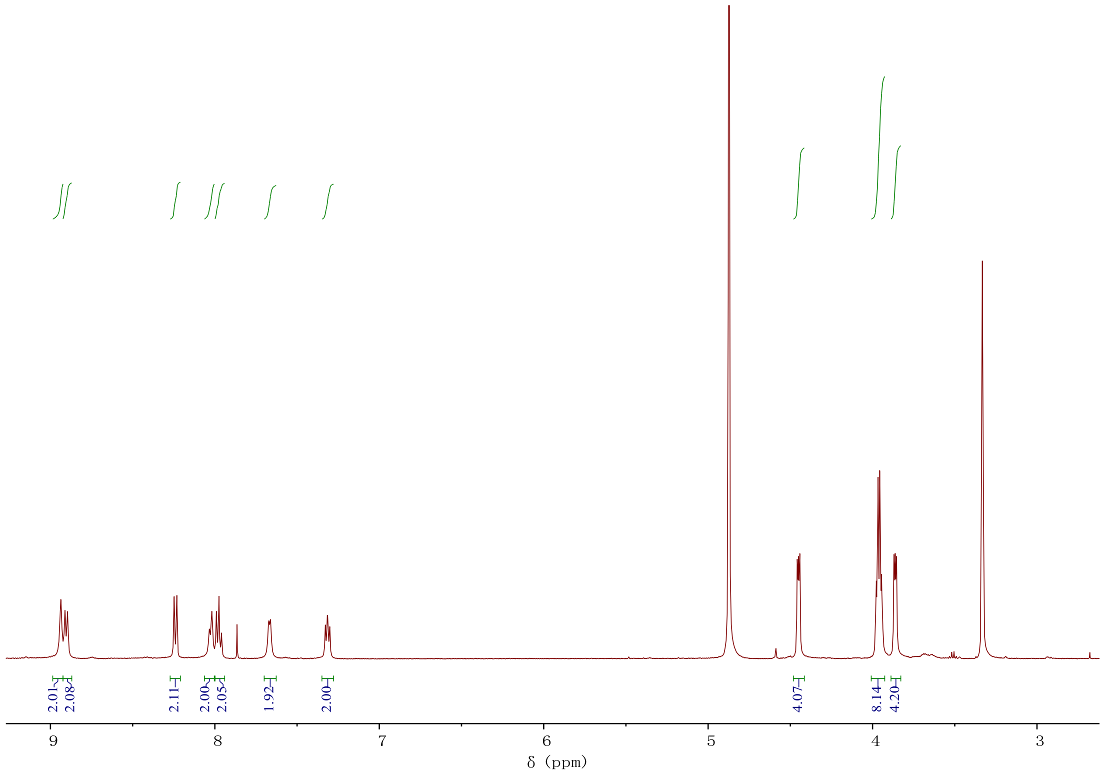

Figure S7 ^{1}H spectrum of **3** in CD_3OD

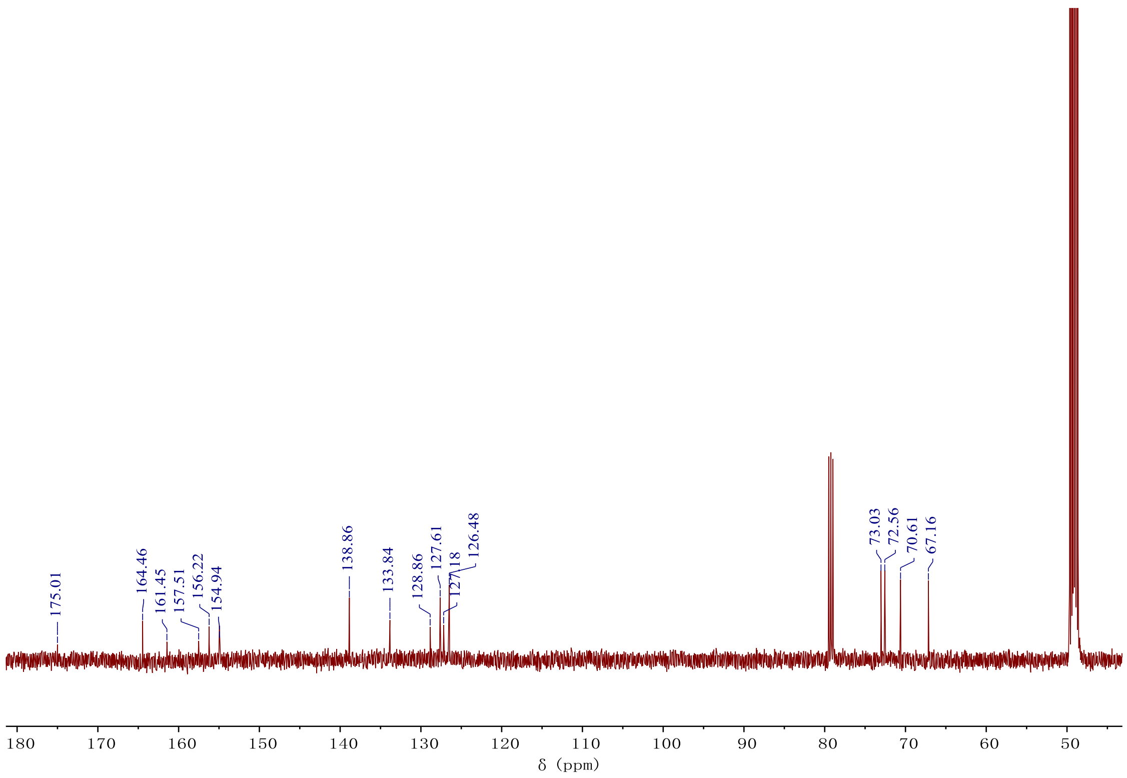

Figure S8 ^{13}C spectrum of **3** in CD_3OD

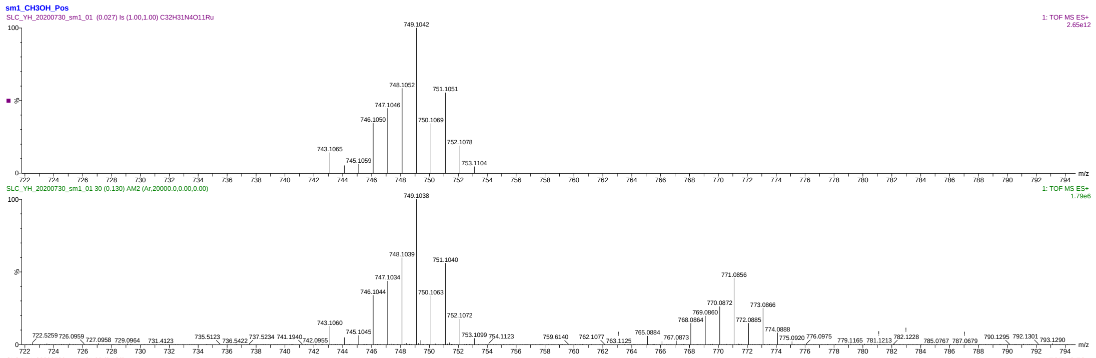

Figure S9 HRMS of **3** (lower) and calculated mass spectrum (upper).

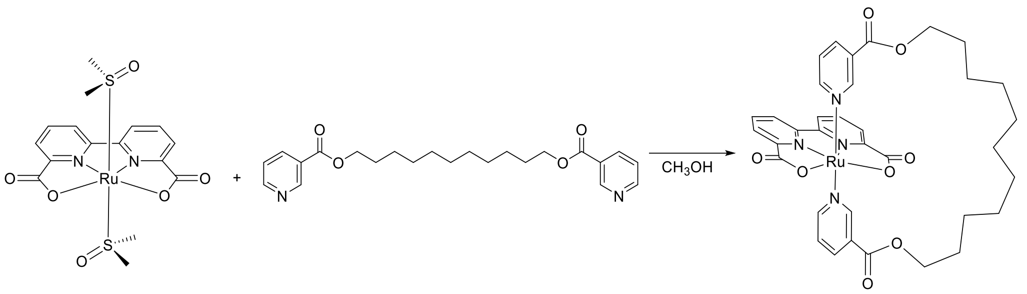

Ru(bda)(DMSO)2 L4 4

4 was synthesized following a similar procedure as reported here for 1. **4** (yield 13%): ^{1}H NMR (400 MHz, $CDCl_3$) δ 9.64 (s, 2H), 8.25 (d, J = 7.9 Hz, 2H), 8.16 (d, J = 8.0 Hz, 2H), 8.12 (d, J = 7.7 Hz, 2H), 7.76 (t, J = 7.8 Hz, 2H), 6.94 – 6.87 (m, 2H), 6.64 (d, J = 5.4 Hz, 2H), 4.36 (t, J = 6.8 Hz, 4H), 1.97 – 1.88 (m, 4H), 1.54 (m, J = 11.4 Hz, 14H). ^{1}H NMR (500 MHz, CD_3OD) δ 9.55 (s, 2H), 8.63 (d, J = 7.4 Hz, 2H), 8.24 (dt, J = 8.0, 1.5 Hz, 2H), 8.05 (d, J = 7.7 Hz, 2H), 7.95 (t, J = 7.9 Hz, 2H), 7.16 (dd, J = 7.8, 5.8 Hz, 2H), 7.02 (d, J = 5.3 Hz, 2H), 4.38 (t, J = 6.2 Hz, 4H), 1.89 (dt, J = 14.3, 6.1 Hz, 4H), 1.61 – 1.47 (m, 14H). The extra peaks a 0.88 and 1.29 ppm can be assigned to the grease from the column. Singlet peak around 2.63 ppm can be assigned to DMSO. Other small peaks may be from the solvent, but we cannot assign them at the moment. ^{13}C NMR (126 MHz, $CDCl_3$) δ 172.88, 163.73, 159.14, 158.07, 156.77, 151.36, 137.67, 130.72, 128.14, 125.90, 124.28, 123.30, 66.88, 28.67, 28.53, 28.48, 28.18, 25.55. HR-MS (calcd for $C_{35}H_{37}N_4O_8Ru(II)^+$ [**4** + H^+]$^+$: 743.1664; found: 743.1663)

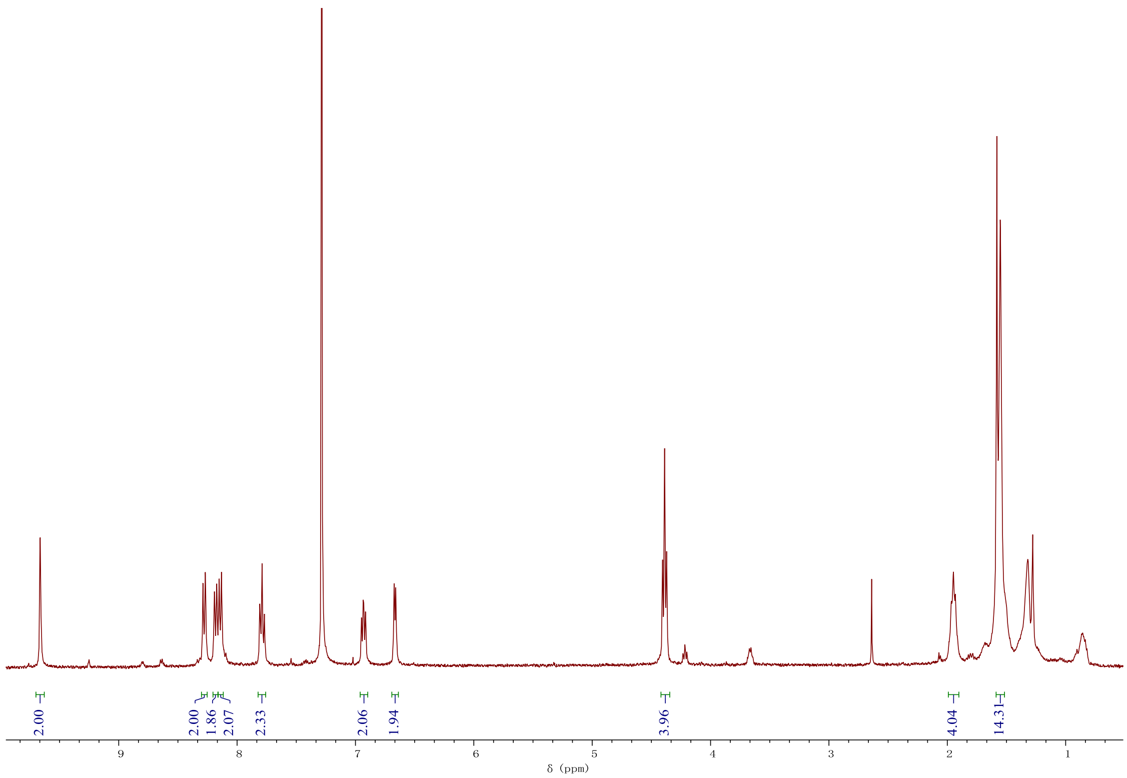

Figure S10 ^{1}H spectrum of **4** in $CDCl_3$.

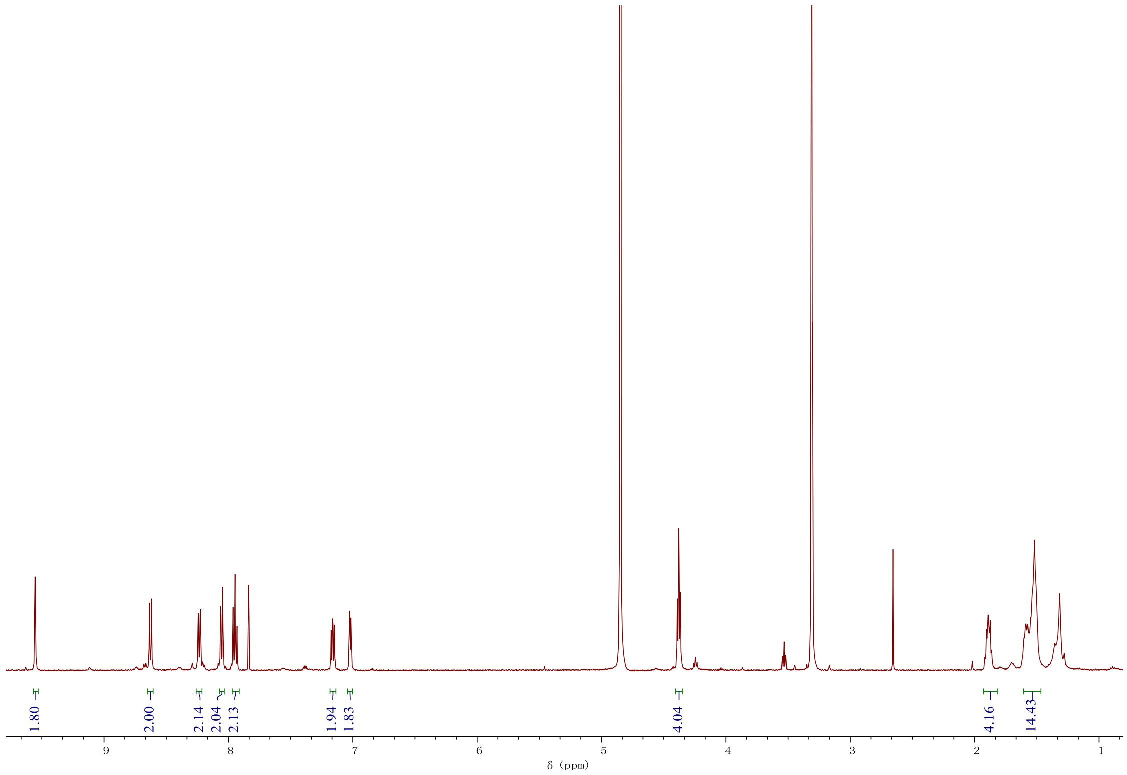

Figure S11 ^{1}H spectrum of **4** in CD_3OD.

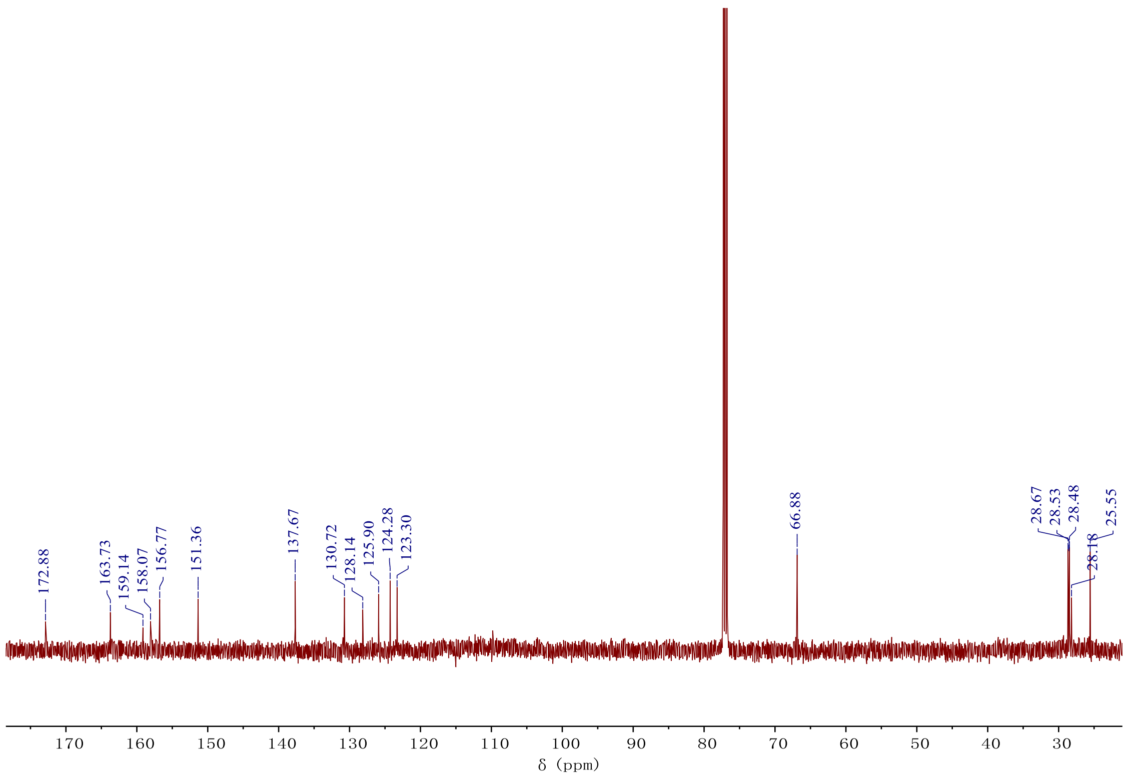

Figure S12 ^{13}C spectrum of **4** in $CDCl_3$

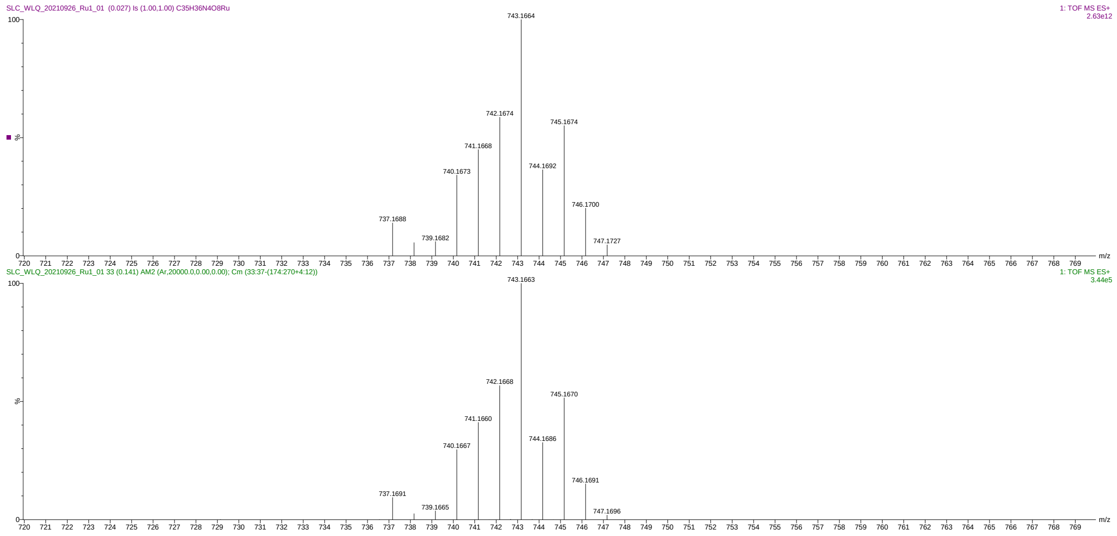

Figure S13 HRMS of **4** (lower) and calculated mass spectrum (upper).

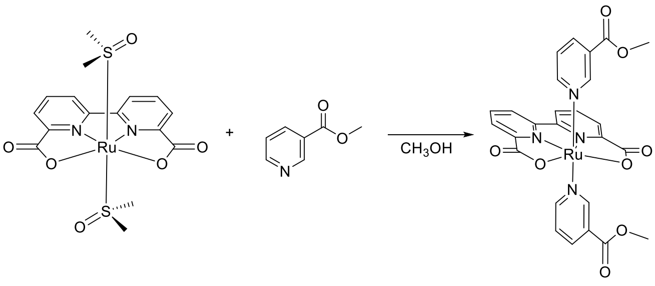

Ru(bda)(DMSO)$_2$ 5

5 was synthesized following a similar procedure as reported here for **1**. ^{1}H NMR (500 MHz, CD_3OD) δ 8.70 (d, *J* = 8.0 Hz, 2H), 8.51 (s, 2H), 8.22 (d, *J* = 7.9 Hz, 2H), 8.06 (d, *J* = 6.0 Hz, 2H), 8.03 – 7.95 (m, 4H), 7.38 – 7.32 (m, 2H), 3.87 (s, 6H). ^{13}C NMR (126 MHz, CD_3OD) δ 175.01, 164.97, 161.31, 157.55, 156.74, 154.41, 138.70, 134.07, 129.04, 127.40, 126.68, 126.28, 53.34. HR-MS (calcd for $C_{26}H_{21}N_4O_8Ru(II)^+$ [**5** + H^+]$^+$: 619.0410; found: 619.0411)

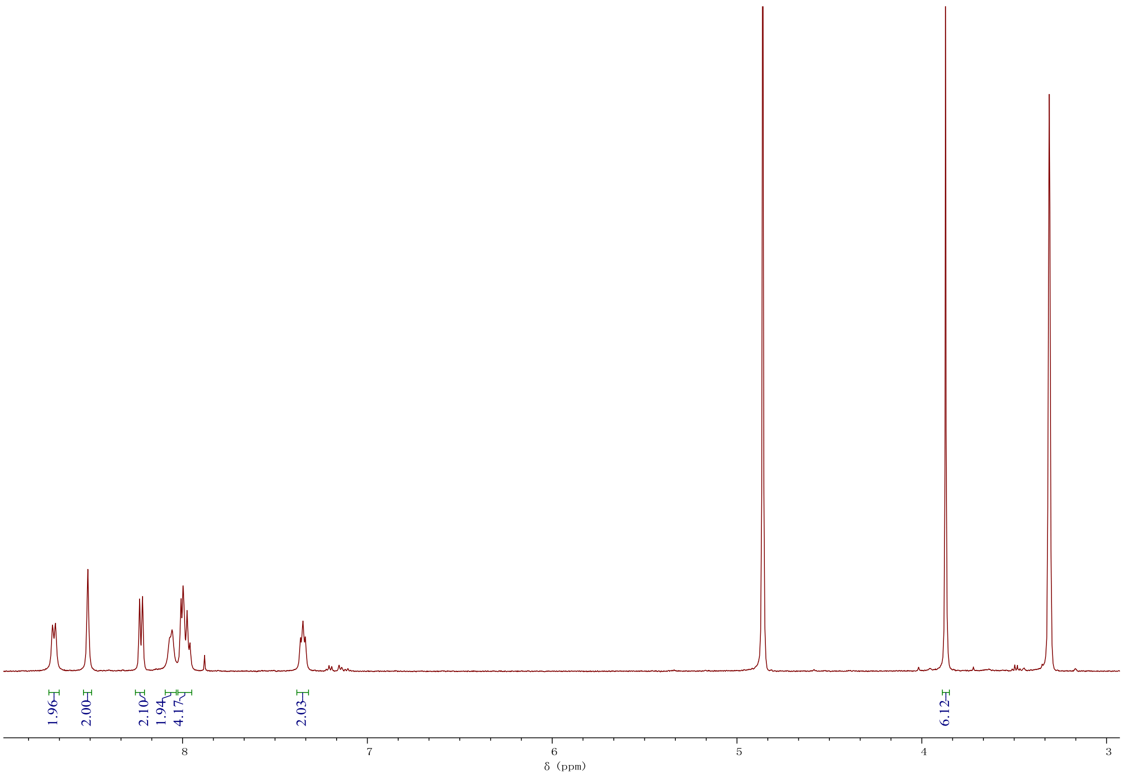

Figure S14 ^{1}H spectrum of **5** in CD_3OD

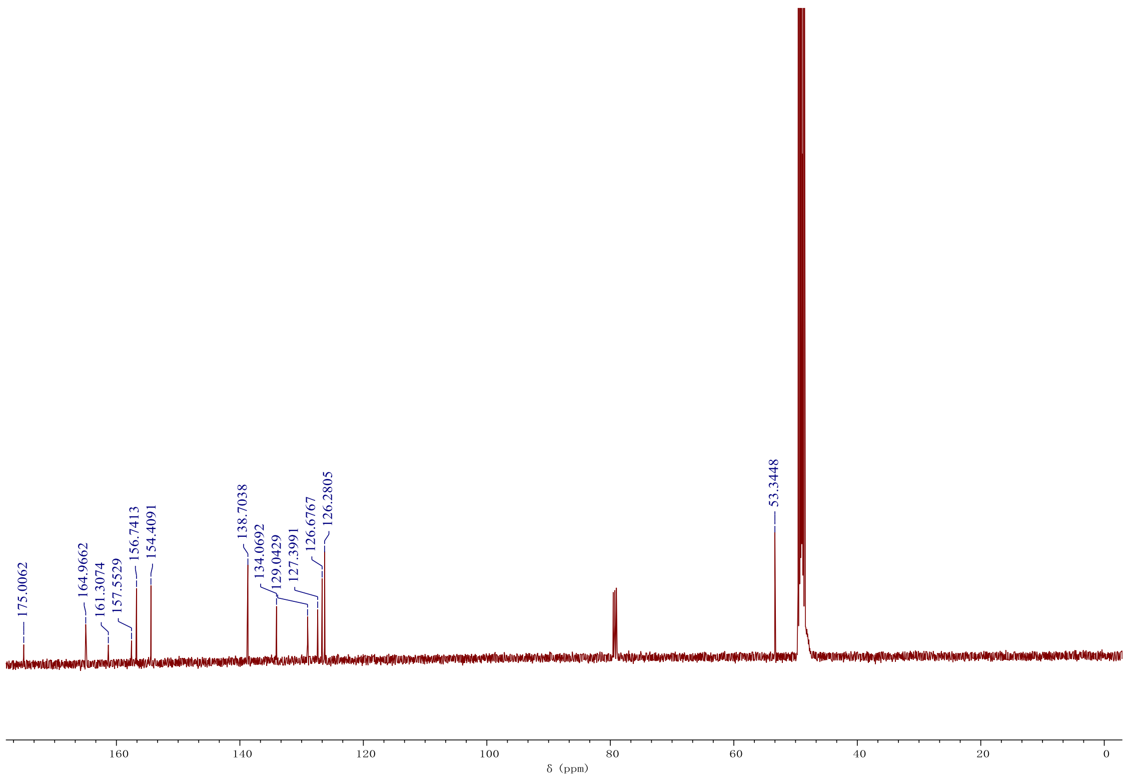

Figure S15 ^{13}C spectrum of **5** in CD_3OD

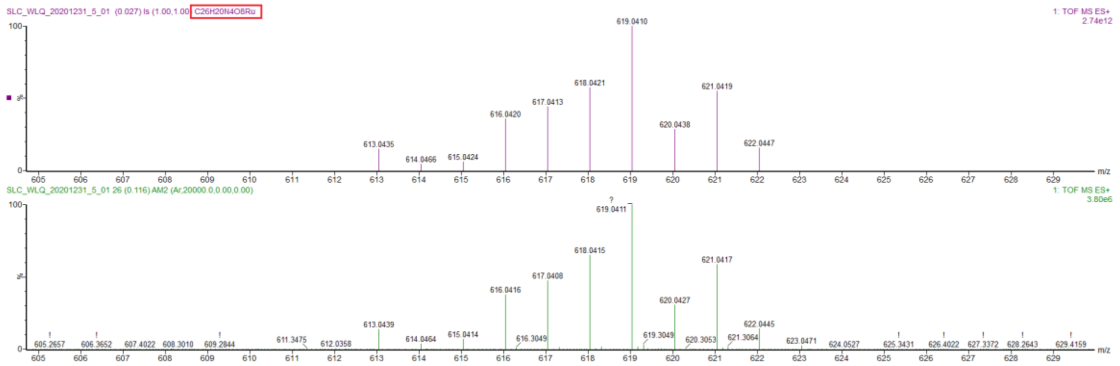

Figure S16 HRMS of **5** (lower) and calculated mass spectrum (upper).

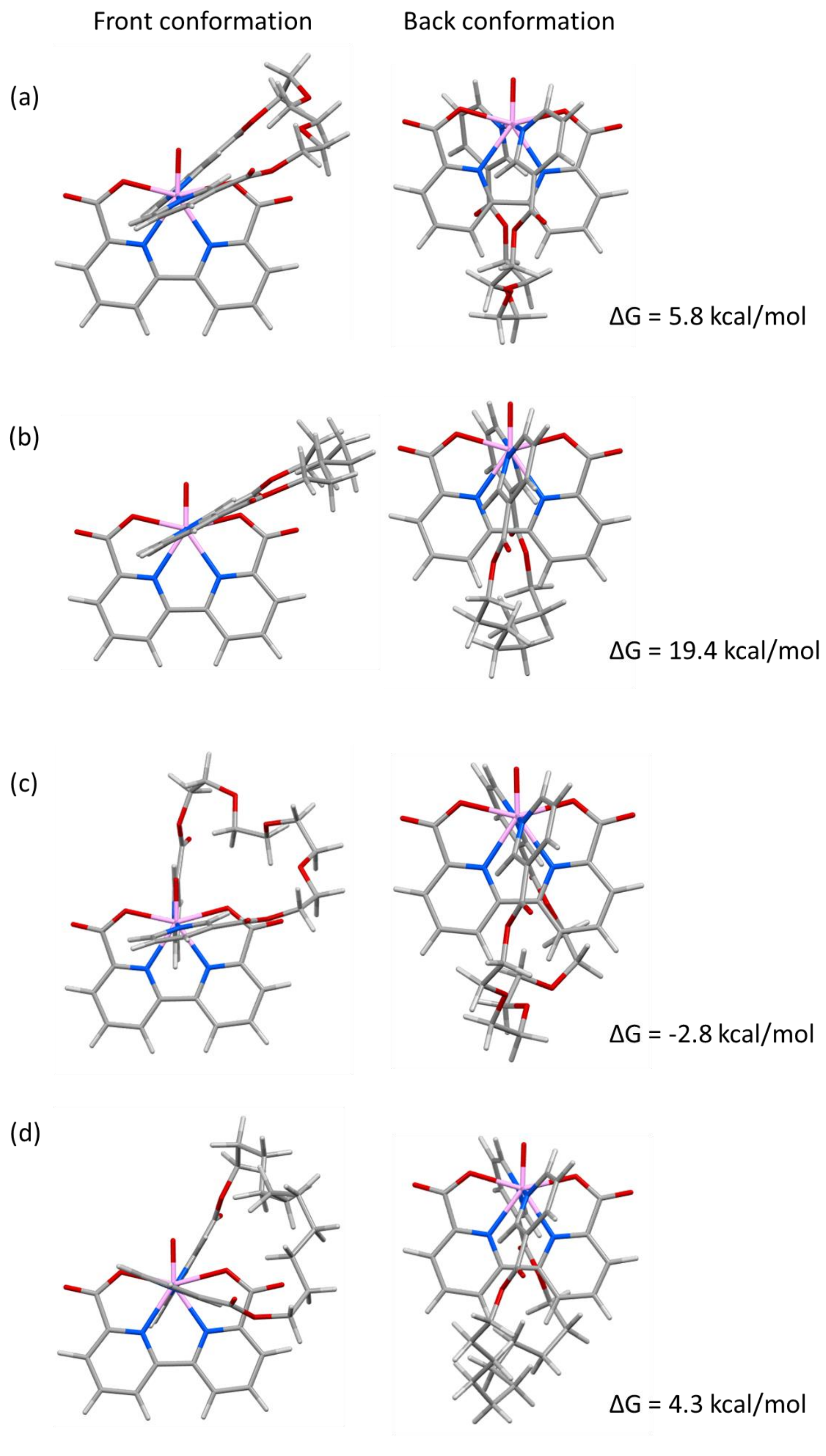

Figure S17 Optimized structures of complex **1**(O)$^+$ - **4**(O)$^+$ at Ru^V states. ΔG is the energy difference between the corresponding "back" and "front" conformation.

Table S2 Spin density on Ru and O atoms for catalysts **1**-**4**

Catalyst	Spin density on Ru	Spin density on O
1	0.34291	0.72990
2	0.34467	0.72575
3	0.34511	0.72506
4	0.34464	0.72544

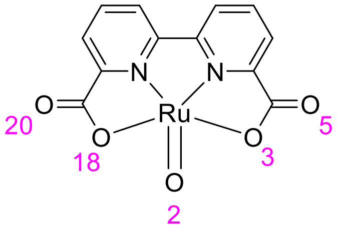

Table S3 Average H-bonds formed between different parts of the catalyst and water during 100 ns MD simulations

Catalyst	Distal ligang	O2	O3	O5	O18	O20
**1**	0.898	0.014	0.132	0.588	0.028	0.576
**2**	0.000	0.028	0.020	0.613	0.126	0.553
**3**	0.749	0.026	0.050	0.508	0.087	0.504
**4**	0.002	0.027	0.110	0.474	0.098	0.527

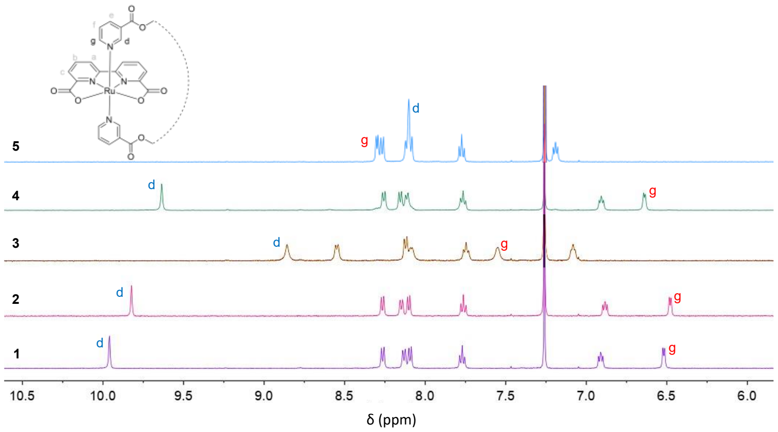

Figure S18 ^{1}H NMR spectra of complex **1**-**5** in $CDCl_3$.

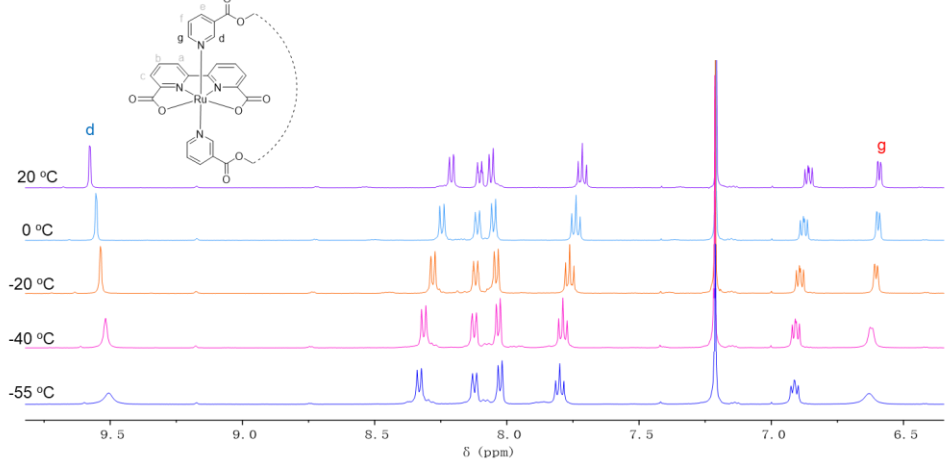

Figure S19 VT ^{1}H NMR spectra of **4** in $CDCl_3$.

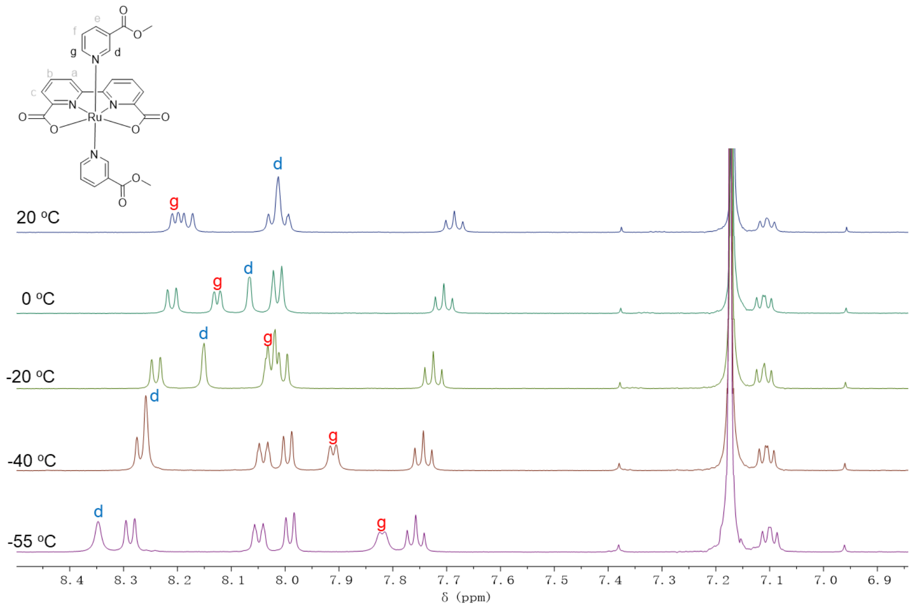

Figure S20 VT ^{1}H NMR spectra of **5** in $CDCl_3$.

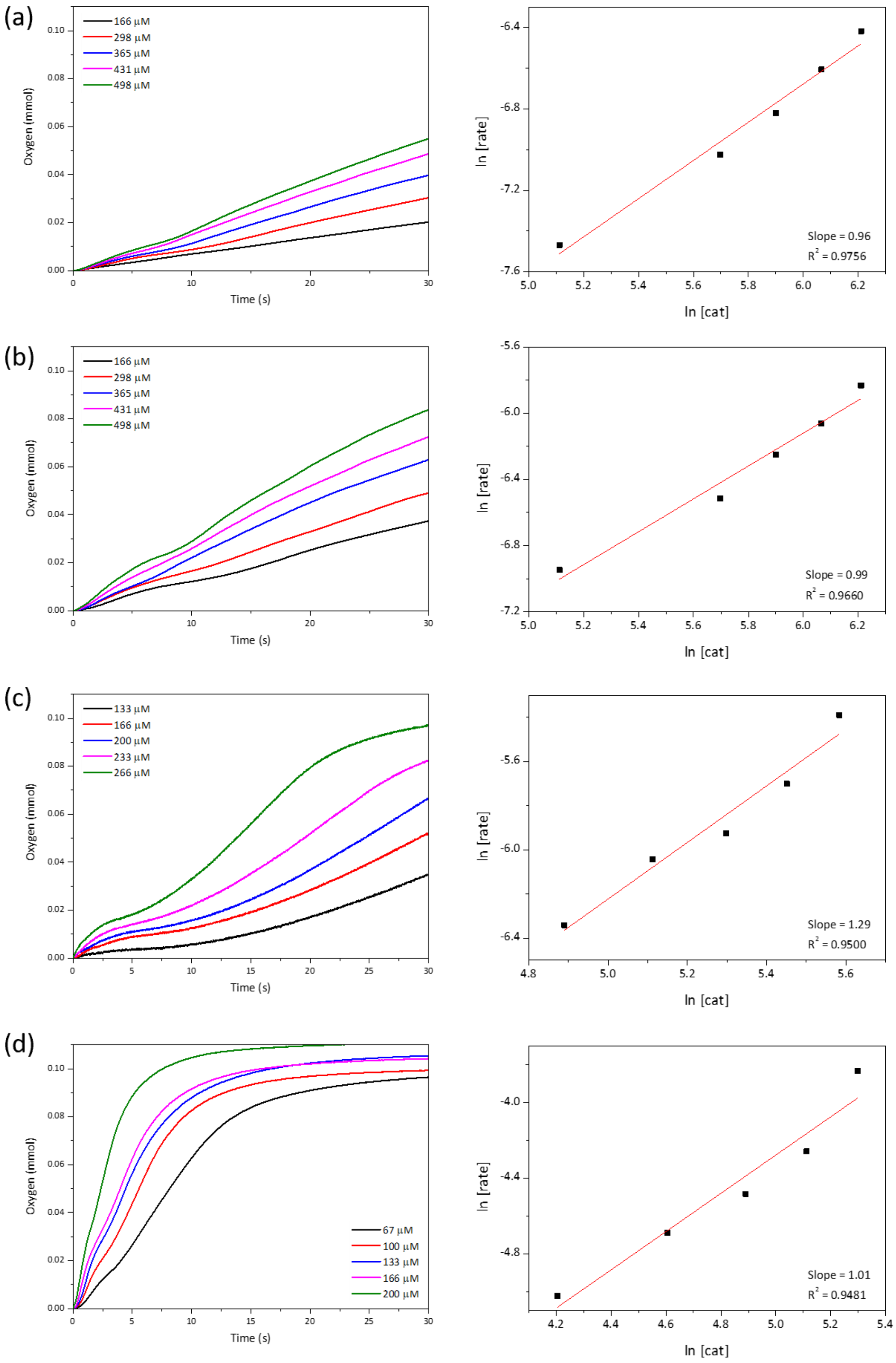

Figure S21 a) Initial phase of oxygen evolution vs time at various concentrations of catalysts **1**-**4** (a-d) in 1:10 CF_3CH_2OH/water (pH 1, acid: trifluoromethane sulfonic acid), $[Ce^{IV}]$ = 0.12 M; and corresponding reaction order determinations.

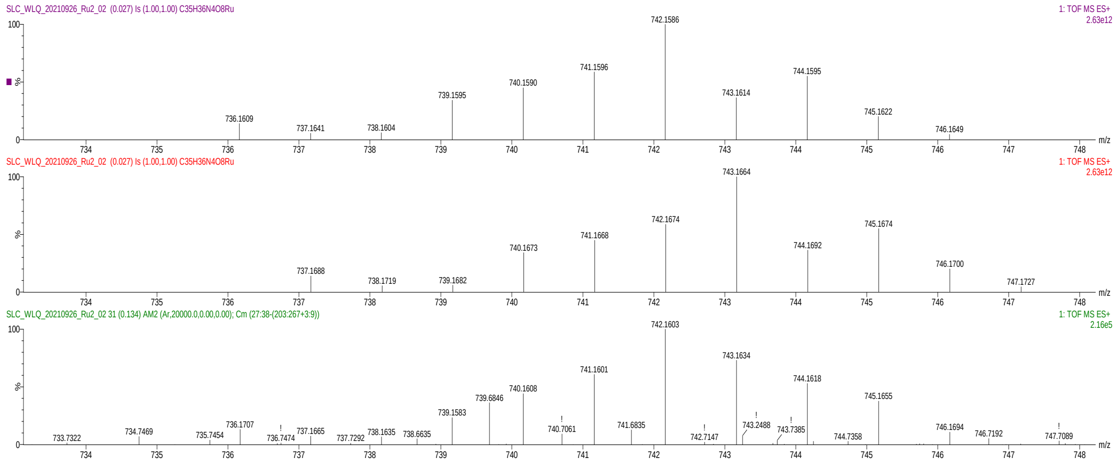

Figure S22 Observed mass spectra of Ru specie after addition of Ce^{IV} to catalyst **4** (lower), and calculated mass spectrum of $[C_{35}H_{36}N_4O_8Ru^{III}]^+$ (upper) and $[C_{35}H_{36}N_4O_8Ru^{II}+H^+]^+$ (middle).

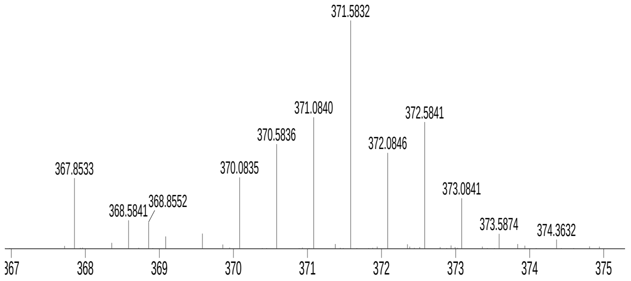

Figure S23 Observed mass spectra of Ru specie after addition of Ce^{IV} to catalyst **4**, which is tentatively assigned to$[C_{35}H_{36}N_4O_8Ru^{III}+H^+]^{2+}$.

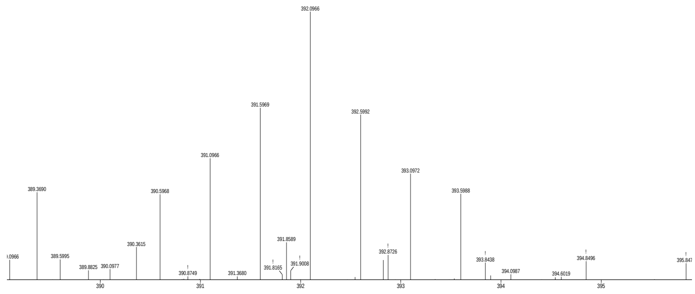

Figure S24 Observed mass spectra of Ru specie after addition of Ce^{IV} to catalyst **4**, which is tentatively assigned to$[C_{35}H_{36}N_4O_8Ru^{III}+CH_3CN+H^+]^{2+}$.

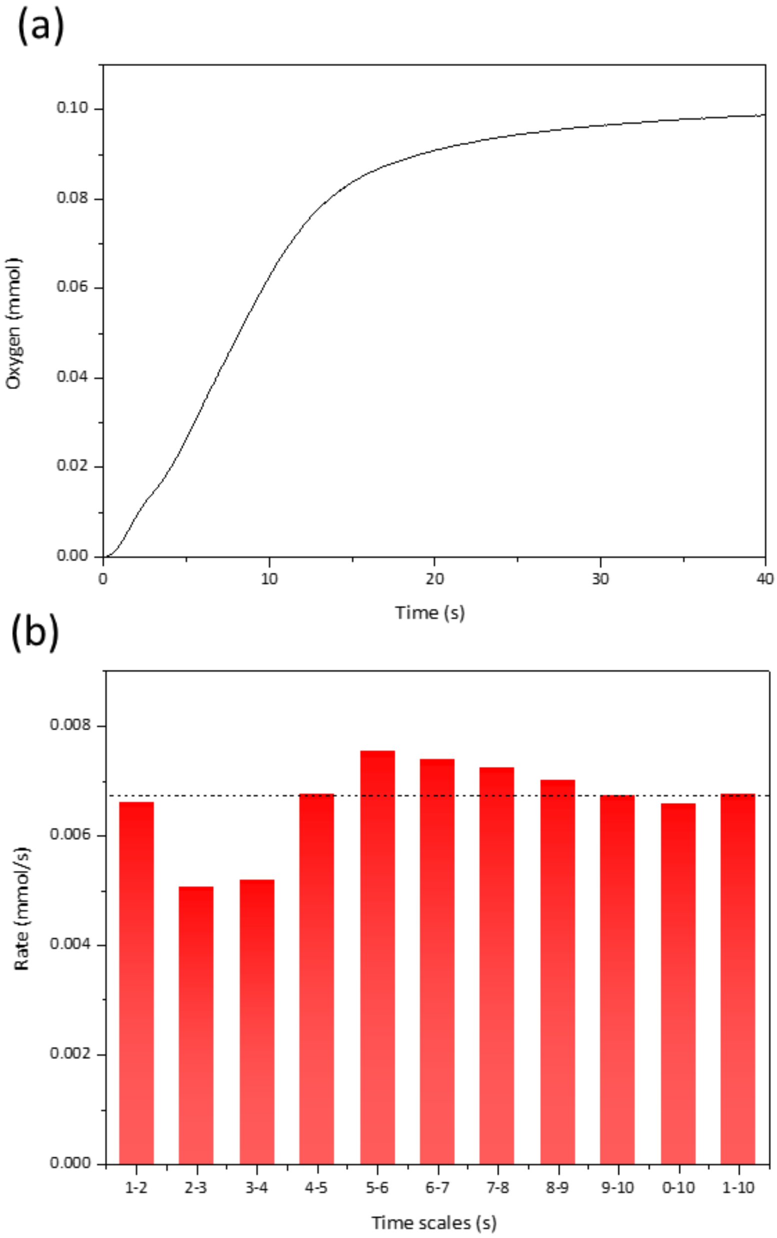

Figure S25 (a) Oxygen evolution curve of 4 vs time, [cat] = 67 µM; (b) oxygen evolution rate at various time scales.

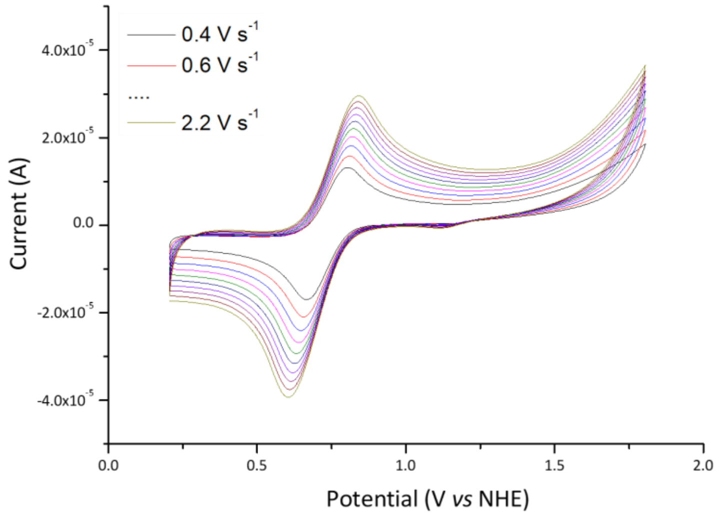

Figure S26 Background subtracted CVs of complex **1** in 0.1 M NaH_2PO_4 aqueous solution containing 30% CF_3CH_2OH at different scan rates from 0.4 to 2.2 V s^{-1}, [cat]= 0.7 mM.

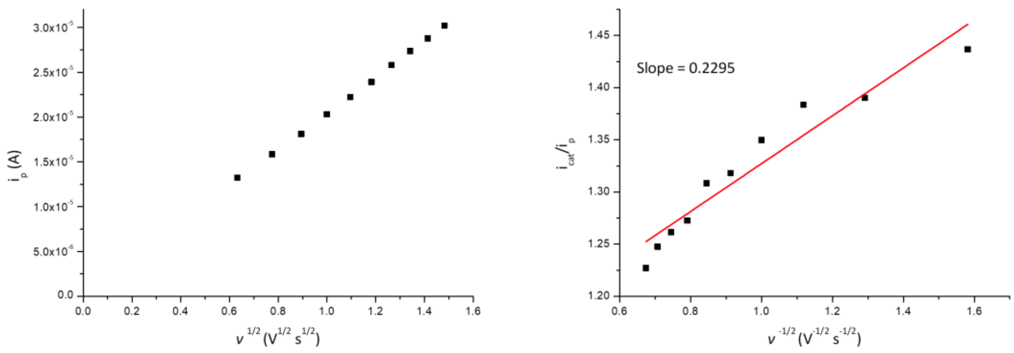

Figure S27 Plot of the background subtracted peak current ($Ru^{III/II}$ couple) for complex **1** *vs.* the square root of scan rate (left); plot of the background subtracted i_{cat}/i_p for complex **1** *vs.* $v^{-1/2}$ (right), I_{cat} measured at 1.8 V.

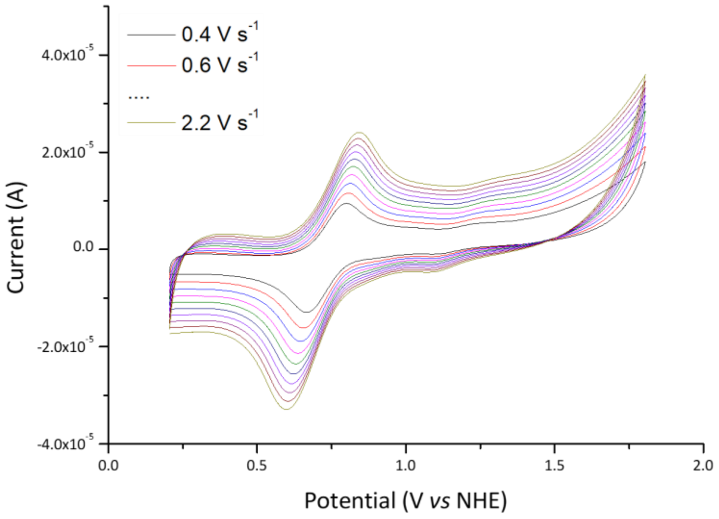

Figure S28 Background subtracted CVs of complex **2** in 0.1 M NaH_2PO_4 aqueous solution containing 30% CF_3CH_2OH at different scan rates from 0.4 to 2.2 V s^{-1}, [cat]= 0.7 mM.

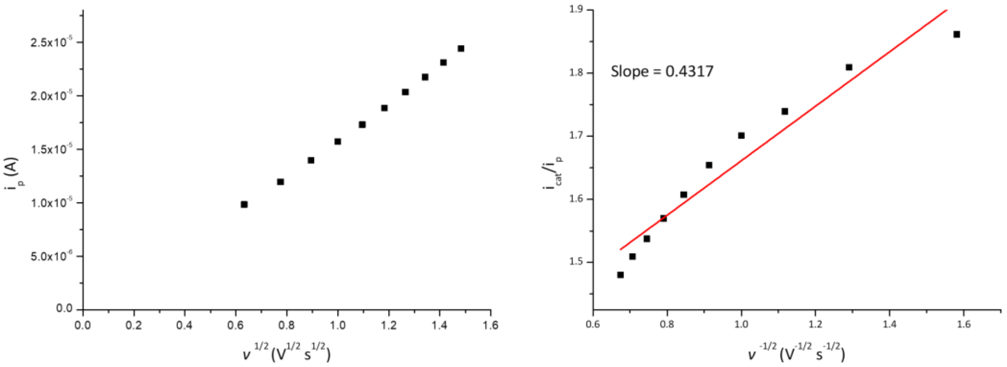

Figure S29 Plot of the background subtracted peak current ($Ru^{III/II}$ couple) for complex **2** *vs.* the square root of scan rate (left); plot of the background subtracted i_{cat}/i_p for complex **2** *vs.* v $^{-1/2}$ (right), I_{cat} measured at 1.8 V.

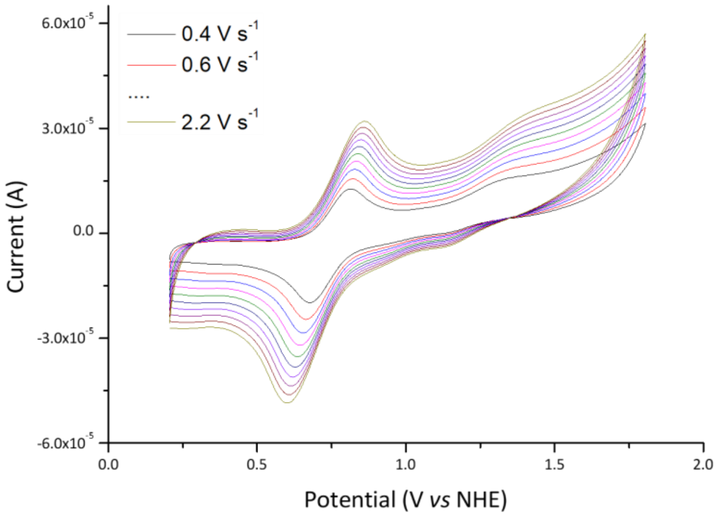

Figure S30 Background subtracted CVs of complex **3** in 0.1 M NaH_2PO_4 aqueous solution containing 30% CF_3CH_2OH at different scan rates from 0.4 to 2.2 V s^{-1}, [cat]= 0.7 mM.

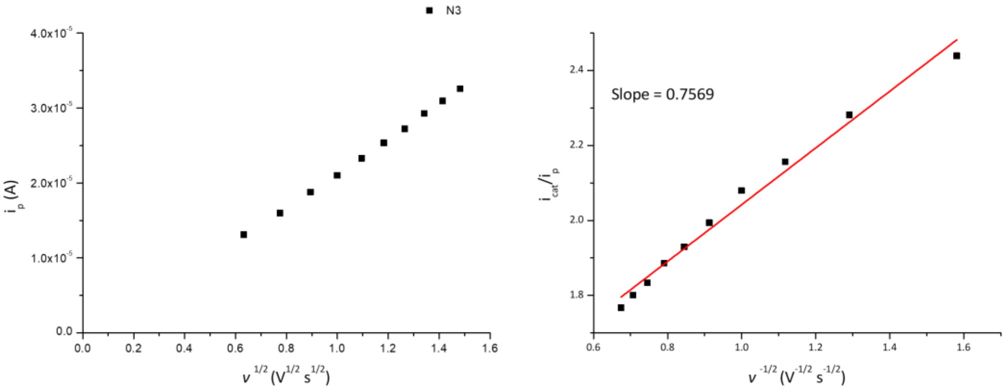

Figure S31 Plot of the background subtracted peak current ($Ru^{III/II}$ couple) for complex **3** *vs.* the square root of scan rate (left); plot of the background subtracted i_{cat}/i_p for complex **3** *vs.* $v^{-1/2}$ (right), I_{cat} measured at 1.8 V.

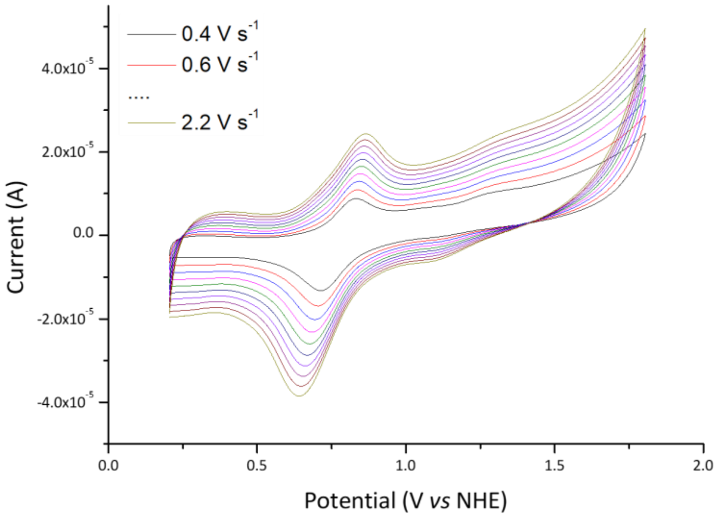

Figure S32 Background subtracted CVs of complex **4** in 0.1 M NaH_2PO_4 aqueous solution containing 30% CF_3CH_2OH at different scan rates from 0.4 to 2.2 V s^{-1}, [cat]= 0.7 mM.

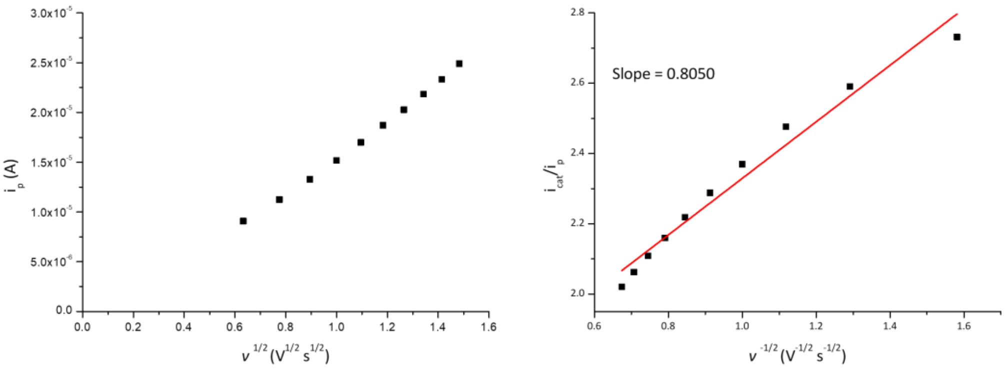

Figure S33 Plot of the background subtracted peak current ($Ru^{III/II}$ couple) for complex **4** *vs.* the square root of scan rate (left); plot of the background subtracted i_{cat}/i_p for complex **4** *vs.* $v^{-1/2}$ (right), I_{cat} measured at 1.8 V.

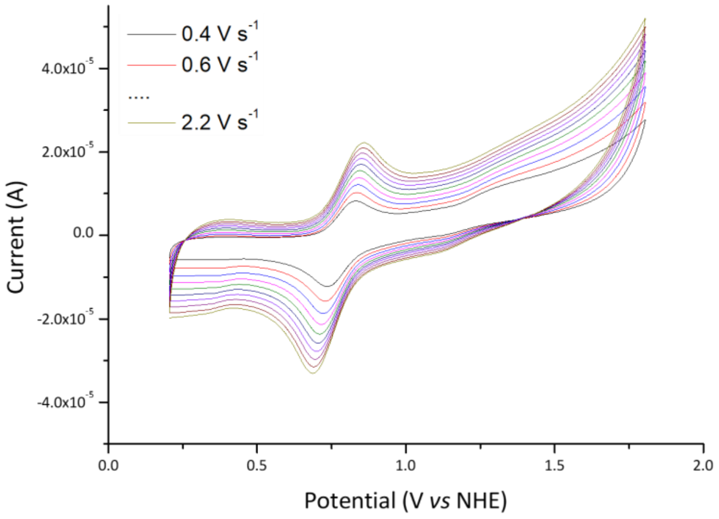

Figure S34 Background subtracted CVs of complex **5** in 0.1 M NaH_2PO_4 aqueous solution containing 30% CF_3CH_2OH at different scan rates from 0.4 to 2.2 V s^{-1}, [cat]= 0.7 mM.

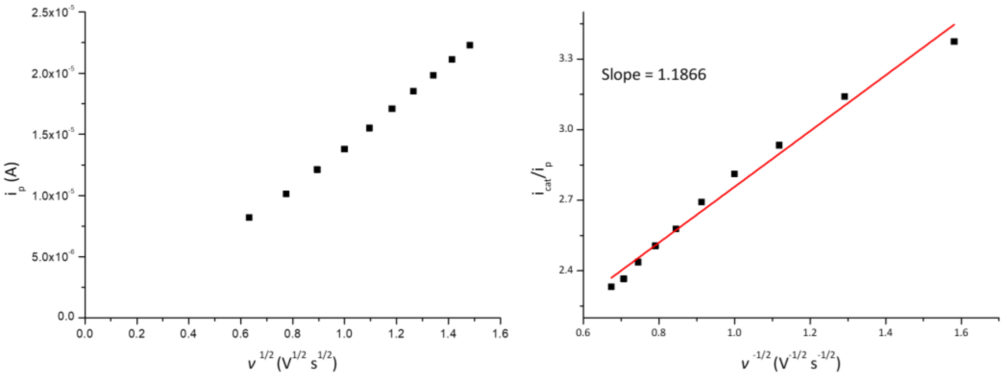

Figure S35 Plot of the background subtracted peak current ($Ru^{III/II}$ couple) for complex **5** *vs.* the square root of scan rate (left); plot of the background subtracted i_{cat}/i_p for complex **45** *vs.* $v^{-1/2}$ (right), I_{cat} measured at 1.8 V.

Table S4 Calculated redox potentials for complex **1-4** vs NHE

Catalyst	$E^{III/II}$	$E^{IV/III}$	$E^{V/IV}$
**1**	0.76	1.16	1.35
**2**	0.91	0.97	1.38
**3**	0.78	1.11	1.44
**4**	0.80	1.08	1.40

Table S5 Measured redox potentials for complex **1-4** vs NHE.

Catalyst	$E^{III/II}$ (V)	$E^{IV/III}$ (V)	$E^{V/IV}$ (V)
**1**	0.73	/	1.18
**2**	0.73	0.95	1.14
**3**	0.74	0.99	1.17
**4**	0.77	0.95	1.14

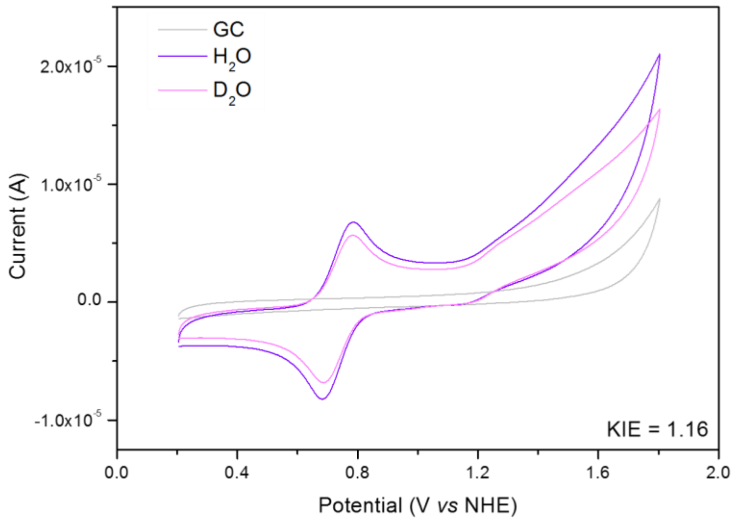

Figure S36 CVs of complex **1** at a scan rate of 0.1 V s^{-1} in 0.1 M NaH_2PO_4 aqueous solution (H_2O and D_2O) containing 30% CF3CH2OH; [cat]= 0.7 mM.

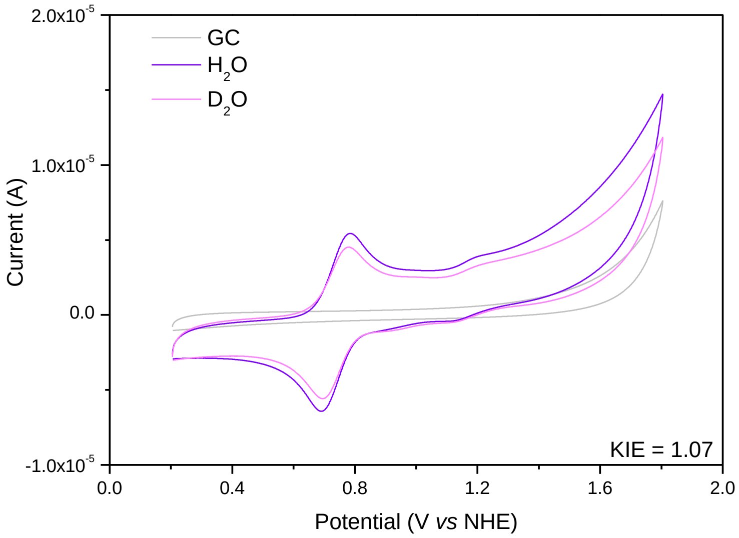

Figure S37 CVs of complex **2** at a scan rate of 0.1 V s^{-1} in 0.1 M NaH_2PO_4 aqueous solution (H_2O and D_2O) containing 30% CF_3CH_2OH; [cat]= 0.7 mM.

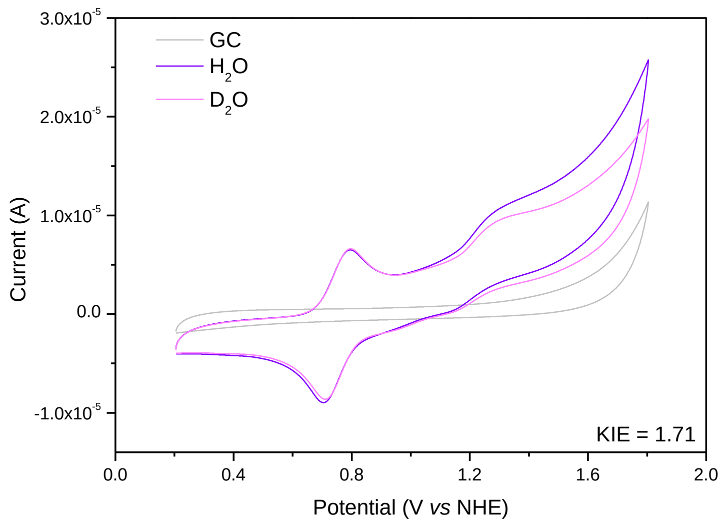

Figure S38 CVs of complex **3** at a scan rate of 0.1 V s^{-1} in 0.1 M NaH_2PO_4 aqueous solution (H_2O and D_2O) containing 30% CF_3CH_2OH; [cat]= 0.7 mM.

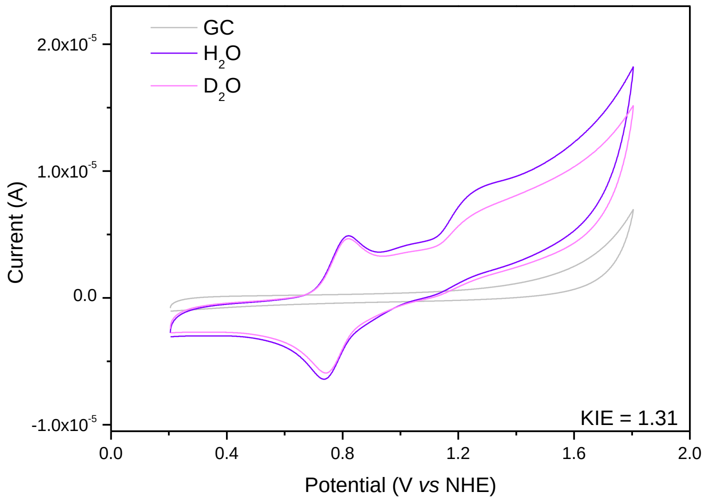

Figure S39 CVs of complex **4** at a scan rate of 0.1 V s^{-1} in 0.1 M NaH_2PO_4 aqueous solution (H_2O and D_2O) containing 30% CF_3CH_2OH; [cat]= 0.7 mM.

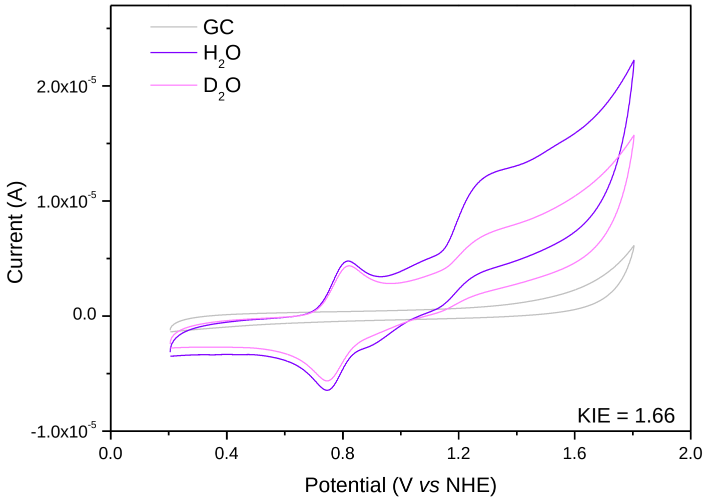

Figure S40 CVs of complex **5** at a scan rate of 0.1 V s^{-1} in 0.1 M NaH_2PO_4 aqueous solution (H_2O and D_2O) containing 30% CF_3CH_2OH; [cat]= 0.7 mM.

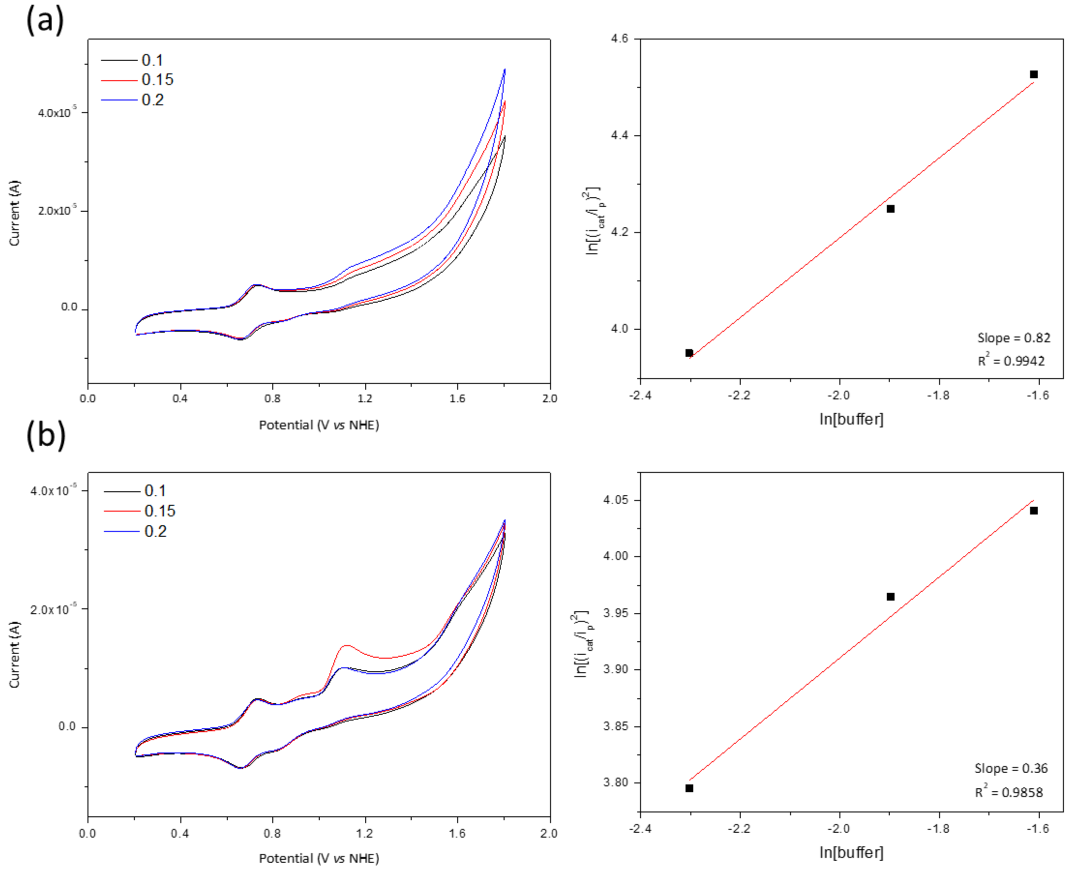

Figure S41 CVs of 0.7 mM (a) **3** and (b) **4** in $H_2PO_4^-/HPO_4^{2-}$ buffers buffer solution (pH 7.1) containing 30% CF_3CH_2OH and the corresponding reaction orders in [$H_2PO_4^-/HPO_4^{2-}$]; buffer concentrations are 0.1 M, 0.15 M and 0.2 M, ionic strength kept at 0.5 M with $NaClO_4$.

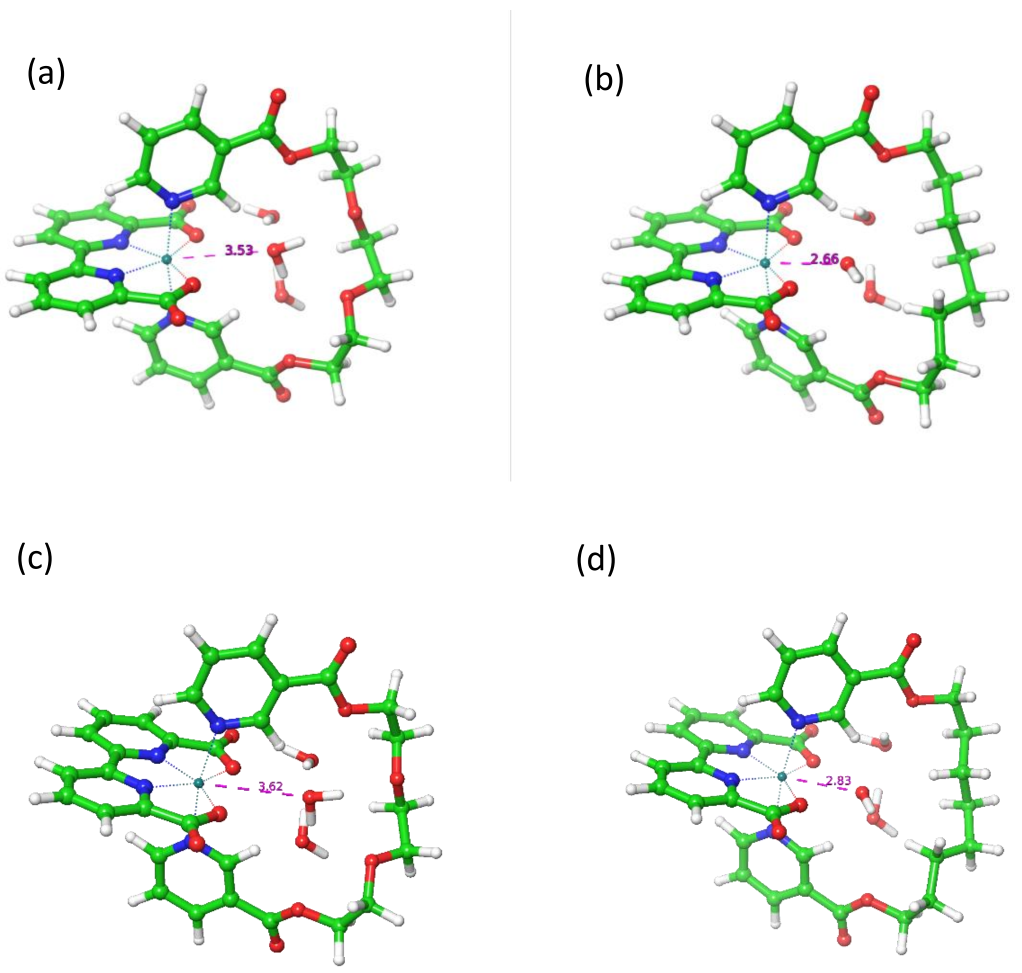

Figure S42 Optimized structure of catalyst **1** of Ru^{III} complex in (a) gas phase and with (c) PCM solvation model, catalyst **2** of Ru^{III} complex in (b) gas phase and with (d) PCM solvation model by replacing the two O atoms on the distal ligand to CH_2.

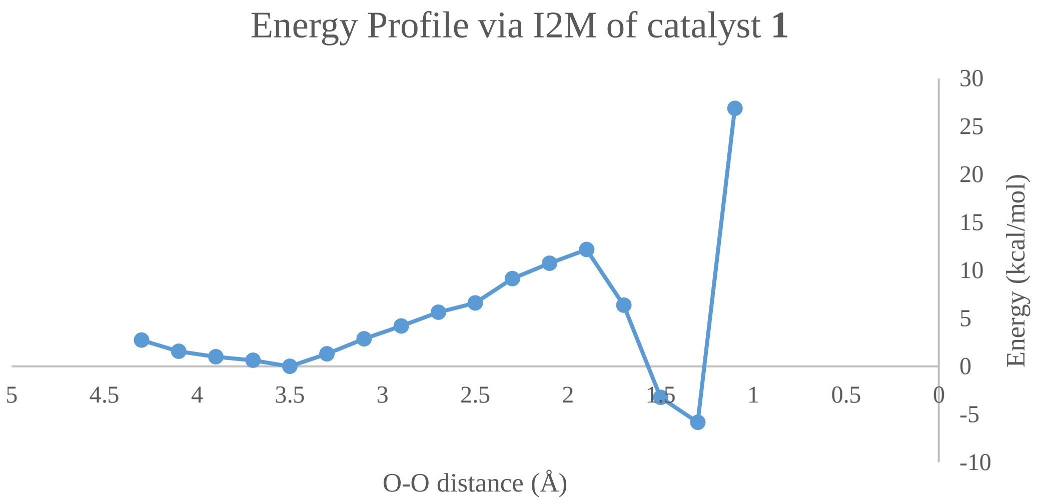

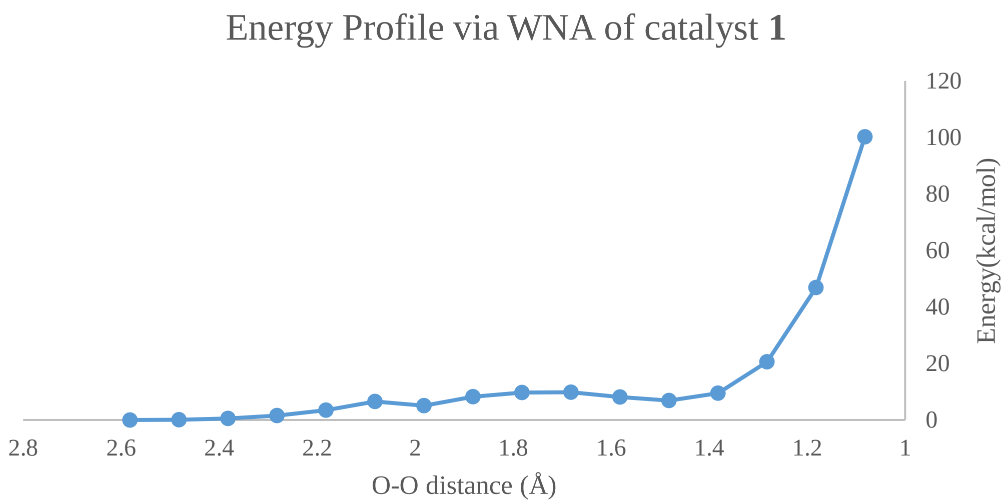

Figure S43 Energy profile of catalyst **1** via I2M and WNA mechanism

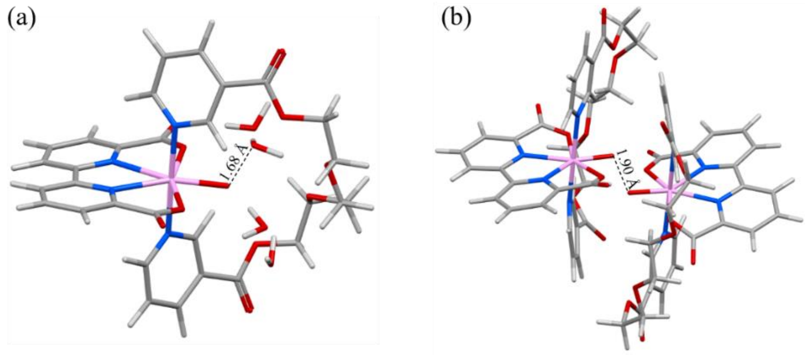

Figure S44 Transition state structure of catalyst **1** via (a) WNA and (b) I2M mechanism. Color code: Ru pink, O red, N blue, C grey and H white.

Catalyst 1 at Ru^{II} oxidation state

E(B3LYP-D3/LACV3P**++) (a.u.) = -2224.802030

Solvation energy (kcal mol^{-1}) = -34.023

Zero-point energy (kcal mol^{-1}) = 337.976

ΔH_{298} (kcal mol^{-1}) = 24.989

ΔS_{298} (cal K^{-1} mol^{-1}) = 238.228

Cartesian coordinates

Ru1	-1.1284820175000	-0.1124805170000	-0.1728093296000
O2	1.9794095012000	5.5887849945000	-1.1849554371000
O3	2.2390198038000	3.9319464860000	0.3524081807000
O4	4.1200335469000	1.9249124199000	-0.1515879551000
O5	4.7360155861000	-1.5597921515000	-0.7869319811000
O6	2.7102615531000	-3.5335912079000	-1.0280711022000
O7	2.4124547168000	-5.6227098030000	-0.1962409050000
O8	0.9846081830000	0.0314564443000	0.5288580957000
O9	2.1117903942000	0.3864737121000	2.4663544304000
O10	-1.6867260369000	-0.5614812056000	-2.2478496828000
O11	-3.5772262565000	-0.9524612621000	-3.4465812801000
N12	-1.0664825241000	1.9433913030000	-0.7145874453000
N13	-0.8900839766000	-2.1748065210000	0.1513532243000
N14	-3.0878409270000	-0.1778670762000	-0.0484437636000
N15	-1.3358522345000	0.2526355895000	1.7588958738000
C16	-3.8095513967000	-0.4806898849000	-1.1382674568000
C17	-5.1952681384000	-0.5600039354000	-1.0448555494000
H18	-5.7570073889000	-0.8035854924000	-1.9398317990000
C19	-5.7995203121000	-0.3229110708000	0.1957682650000
H20	-6.8792634239000	-0.3783370982000	0.2950820443000
C21	-5.0210834230000	-0.0177030513000	1.3184471568000
H22	-5.4865643491000	0.1594858111000	2.2824662864000
C23	-3.6334315580000	0.0513463831000	1.1846839973000
C24	-2.6180810962000	0.3048501419000	2.2304586058000
C25	-2.8222850870000	0.5497199666000	3.5897665996000
H26	-3.8285876680000	0.6003753104000	3.9921049835000
C27	-1.7114879768000	0.7252996512000	4.4223665828000
H28	-1.8609115412000	0.9186934727000	5.4801158003000
C29	-0.4148120014000	0.6453950828000	3.9009121096000
H30	0.4734922027000	0.7640249129000	4.5111941882000
C31	-0.2568634319000	0.3998813421000	2.5414050391000
C32	-2.9651224381000	-0.6947455211000	-2.4132334080000
C33	1.0809980649000	0.2595090347000	1.8033096130000
C34	-2.0501625412000	2.5754522879000	-1.3891932174000
H35	-2.9479463077000	2.0021060773000	-1.5837471510000
C36	-1.9252582686000	3.8881086603000	-1.8363263311000
H37	-2.7435670933000	4.3431492000000	-2.3840640917000

C38	-0.7465705476000	4.5869945523000	-1.5850199503000
H39	-0.5930919095000	5.6052728482000	-1.9279196028000
C40	0.2651375537000	3.9380278620000	-0.8759051073000
C41	0.0719973016000	2.6196695193000	-0.4711819495000
H42	0.8650907240000	2.0701049590000	0.0137987208000
C43	1.5777824992000	4.6007835960000	-0.6057705234000
C44	3.6544317828000	4.1465479098000	0.5232158142000
H45	3.8101522034000	4.9075703557000	1.2956244410000
H46	4.0847873242000	4.4987419271000	-0.4180167509000
C47	4.2373778161000	2.8106442062000	0.9421033989000
H48	5.2940297422000	2.9595286155000	1.2294285244000
H49	3.6942828903000	2.4228335365000	1.8170064495000
C50	4.5207657699000	0.5952129659000	0.1647090152000
H51	3.9797170160000	0.2262566084000	1.0439316371000
H52	5.6052409587000	0.5511349466000	0.3610515697000
C53	4.1937176698000	-0.2675207348000	-1.0440282370000
H54	3.1031571752000	-0.3124040233000	-1.1732656780000
H55	4.6398628463000	0.1813230643000	-1.9454639186000
C56	4.6502294077000	-2.4757249108000	-1.8608042588000
H57	5.6472540466000	-2.6539859690000	-2.2944326160000
H58	3.9993623730000	-2.1005888464000	-2.6629704294000
C59	4.0899721443000	-3.7918327375000	-1.3547518209000
H60	4.6166019517000	-4.1304597740000	-0.4576410211000
H61	4.1452420297000	-4.5784736560000	-2.1157044359000
C62	2.0065830732000	-4.5010167628000	-0.4236499220000
C63	0.6466092268000	-4.0065138482000	-0.0484745428000
C64	-0.2844211876000	-4.8758838430000	0.5243148225000
H65	-0.0172292343000	-5.9173598713000	0.6711228590000
C66	-1.5265249992000	-4.3672465953000	0.8942848126000
H67	-2.2848667461000	-5.0022018860000	1.3397122069000
C68	-1.7923811640000	-3.0154371552000	0.6955422051000
H69	-2.7448168446000	-2.5808744511000	0.9776454772000
C70	0.3065539718000	-2.6674300139000	-0.2224382704000
H71	1.0118134126000	-1.9594240959000	-0.6327065495000

Catalyst 1 at Ru^{III} oxidation state

E(B3LYP-D3/LACV3P**++) (a.u.) = -2301.058151

Solvation energy (kcal mol^{-1}) = -62.437

Zero-point energy (kcal mol^{-1}) = 355.255

ΔH_{298} (kcal mol^{-1}) = 26.468

ΔS_{298} (cal K^{-1} mol^{-1}) = 248.500

Cartesian coordinates

Ru1	-1.0061418235000	0.0082236811000	-0.0013080970000
O2	2.0706904029000	5.4362609905000	-1.4150802794000
O3	2.2826404475000	3.9148568462000	0.2701353218000
O4	3.7290470560000	1.5360622264000	-0.4697646671000
O5	4.8437165203000	-1.7845176101000	-1.3216690373000
O6	2.7506778069000	-3.5934853041000	-1.3597295865000
O7	2.4765929335000	-5.5937226666000	-0.3273715152000
O8	0.8341724788000	0.2709101882000	1.0670329758000
O9	1.7074149391000	0.8105563960000	3.0780843664000
O10	-1.3758293015000	-0.3605762888000	-2.0354755393000
O11	-3.0064095083000	-0.8894133667000	-3.5160375120000
N12	-1.1514044309000	2.0974656857000	-0.4512440046000
N13	-0.6659097386000	-2.0544183408000	0.2827977414000
N14	-3.0235400293000	-0.2370242444000	-0.0629025343000
N15	-1.5817848395000	0.2870016439000	1.9479617603000
C16	-3.5906524104000	-0.5523014208000	-1.2358859100000
C17	-4.9648875516000	-0.7459884689000	-1.3262613595000
H18	-5.3854359679000	-1.0003929474000	-2.2931297206000
C19	-5.7367167561000	-0.5984749940000	-0.1710531222000
H20	-6.8119306766000	-0.7413392441000	-0.2108359755000
C21	-5.1274893255000	-0.2642580166000	1.0429913813000
H22	-5.7177148520000	-0.1463783587000	1.9447304861000
C23	-3.7438695622000	-0.0854792197000	1.0797120920000
C24	-2.9056554096000	0.2319557757000	2.2471891332000
C25	-3.3245158145000	0.4458274733000	3.5606982381000
H26	-4.3778188656000	0.4063466271000	3.8133159462000
C27	-2.3654836028000	0.7119363233000	4.5429818600000
H28	-2.6785635851000	0.8833231938000	5.5678817245000
C29	-1.0117941231000	0.7533783529000	4.2040607374000
H30	-0.2261971531000	0.9517869975000	4.9248970830000
C31	-0.6505466837000	0.5306861891000	2.8793157316000
C32	-2.6075232317000	-0.6278729665000	-2.3968114262000
C33	0.7704843405000	0.5440785607000	2.3439078563000
C34	-2.1227149122000	2.6820894326000	-1.1834131210000
H35	-3.0248400372000	2.1054472877000	-1.3489261723000
C36	-1.9722115299000	3.9523380846000	-1.7334836782000
H37	-2.7805692753000	4.3787604983000	-2.3173569301000

C38	-0.7642524723000	4.6314845511000	-1.5686421053000
H39	-0.5760509223000	5.5927269582000	-2.0367663800000
C40	0.2286440057000	4.0306992961000	-0.7949639303000
C41	-0.0168329750000	2.7809905490000	-0.2362647865000
H42	0.7419851755000	2.2858933586000	0.3479113792000
C43	1.6183851161000	4.5811626552000	-0.6895103745000
C44	3.7227603132000	3.8351813763000	0.2397953982000
H45	4.1294557598000	4.5355691232000	0.9755232201000
H46	4.0848520231000	4.1069574206000	-0.7553597110000
C47	4.0806343813000	2.4087440121000	0.6062365751000
H48	5.1652537350000	2.3599683798000	0.7865856831000
H49	3.5613152285000	2.1100426899000	1.5285437325000
C50	4.1675001106000	0.1909981521000	-0.2375604419000
H51	3.4307256206000	-0.3486451611000	0.3711672499000
H52	5.1268798537000	0.1843734282000	0.2964270234000
C53	4.3538860535000	-0.4835511300000	-1.5903428224000
H54	3.3923693176000	-0.5257586675000	-2.1199714192000
H55	5.0583622002000	0.1024928401000	-2.2005895317000
C56	4.7083460772000	-2.7265908021000	-2.3722148576000
H57	5.6906836253000	-2.9763496891000	-2.7981543625000
H58	4.0795310251000	-2.3348350234000	-3.1835375957000
C59	4.0756444305000	-3.9800756912000	-1.8046419282000
H60	4.6390606422000	-4.3589195222000	-0.9478596963000
H61	3.9912475534000	-4.7751013210000	-2.5526113129000
C62	2.0963579881000	-4.4728345412000	-0.5929284857000
C63	0.8144917945000	-3.9008670703000	-0.0650919946000
C64	-0.0344776078000	-4.7349782810000	0.6650300726000
H65	0.2459986013000	-5.7735494212000	0.8085496182000
C66	-1.2091960616000	-4.2055056213000	1.1921536272000
H67	-1.8986567567000	-4.8173671481000	1.7634802600000
C68	-1.4882602574000	-2.8614684674000	0.9840456182000
H69	-2.3858035904000	-2.4094421436000	1.3884212452000
C70	0.4680070269000	-2.5610649070000	-0.2411577581000
H71	1.0958766742000	-1.8720605772000	-0.7934276270000
O72	1.1463901590000	0.2843919691000	-1.4874799545000
H73	1.8403064160000	0.8913736491000	-1.1695781440000
H74	0.8871911931000	0.5411425052000	-2.3817885302000

Catalyst 1 at Ru^{IV} oxidation state

E(B3LYP-D3/LACV3P**++) (a.u.) = -2300.406641

Solvation energy (kcal mol^{-1}) = -64.201

Zero-point energy (kcal mol^{-1}) = 348.402

ΔH_{298} (kcal mol^{-1}) = 25.812

ΔS_{298} (cal K^{-1} mol^{-1}) = 239.933

Cartesian coordinates

Ru1	-0.9093091345000	-0.0876603811000	-0.3337443164000
O2	2.1715085202000	5.6295709234000	-0.9637724697000
O3	2.3800860059000	3.9590143478000	0.5752332513000
O4	3.9619919458000	1.8208035545000	-0.0982197570000
O5	4.1761529696000	-1.6722858241000	-0.9336703021000
O6	2.6042575964000	-3.8786748359000	-1.4923627602000
O7	2.6490053458000	-5.5297019586000	0.0769567024000
O8	0.8543910309000	-0.0162436622000	0.8202023695000
O9	1.8910698752000	0.7113528895000	2.6840833185000
O10	-1.7651536659000	-0.2473165230000	-2.2376787885000
O11	-3.4554563737000	-1.0862687753000	-3.4765166611000
N12	-0.8666720229000	1.9991839202000	-0.6991782997000
N13	-0.7280137587000	-2.1985988346000	0.0005349229000
N14	-3.0543866683000	-0.2839420515000	-0.0904735405000
N15	-1.4606505570000	0.3984516300000	1.7138741678000
C16	-3.7698622756000	-0.6778137537000	-1.1516440566000
C17	-5.1411751999000	-0.8980088756000	-1.0815536817000
H18	-5.6508439535000	-1.2233781578000	-1.9822796923000
C19	-5.7905648250000	-0.6655301124000	0.1297099612000
H20	-6.8606361895000	-0.8214907345000	0.2233185946000
C21	-5.0522729249000	-0.2067103121000	1.2222730207000
H22	-5.5369278339000	0.0042839584000	2.1684603044000
C23	-3.6748881726000	-0.0240461245000	1.0862561109000
C24	-2.7515928382000	0.4224285571000	2.1214289576000
C25	-3.0888351297000	0.8209326927000	3.4162638238000
H26	-4.1272122095000	0.8414294281000	3.7263086869000
C27	-2.0735572246000	1.1895104712000	4.3003008788000
H28	-2.3180667470000	1.5106598200000	5.3078006832000
C29	-0.7475252649000	1.1157901688000	3.8790915974000
H30	0.0975562484000	1.3495586774000	4.5174913359000
C31	-0.4860105868000	0.7035939595000	2.5770428574000
C32	-2.9713882446000	-0.7255192631000	-2.4232936169000
C33	0.8906827569000	0.4774307304000	2.0285737668000
C34	-1.9070450820000	2.6845111800000	-1.2182319385000
H35	-2.8025692826000	2.1140629975000	-1.4306406795000
C36	-1.8230249097000	4.0441963935000	-1.4961481052000
H37	-2.6770451324000	4.5552727229000	-1.9272252943000

C38	-0.6327932083000	4.7222773838000	-1.2283070392000
H39	-0.5137004054000	5.7793619804000	-1.4457691383000
C40	0.4293269780000	4.0109693591000	-0.6715110448000
C41	0.2857678143000	2.6442853036000	-0.4419298915000
H42	1.1208680882000	2.0533511170000	-0.0875101807000
C43	1.7542580488000	4.6529676822000	-0.3840164393000
C44	3.8207584762000	4.0468434730000	0.6966132070000
H45	4.0687821489000	4.7426392281000	1.5035287104000
H46	4.2374859246000	4.4182158570000	-0.2430465806000
C47	4.2884076686000	2.6393497387000	1.0110225926000
H48	5.3761697093000	2.6541746778000	1.1923218857000
H49	3.7912053584000	2.2754097386000	1.9218086213000
C50	4.2289097315000	0.4396091592000	0.1314450641000
H51	3.5438928618000	0.0330414030000	0.8849809321000
H52	5.2617670171000	0.2929192716000	0.4834977289000
C53	4.0377519003000	-0.2809333219000	-1.1936129808000
H54	3.0363976239000	-0.0625646324000	-1.5937141582000
H55	4.7892153335000	0.0686733178000	-1.9177481397000
C56	4.3822789204000	-2.4563633013000	-2.0929706382000
H57	5.4288424916000	-2.4028912578000	-2.4347606811000
H58	3.7384024549000	-2.1189175108000	-2.9201155872000
C59	4.0272730638000	-3.8899702374000	-1.7481836179000
H60	4.5544005033000	-4.2388132556000	-0.8567007562000
H61	4.2336831234000	-4.5680944287000	-2.5816207555000
C62	2.1071467035000	-4.6025140254000	-0.4818709527000
C63	0.7563921144000	-4.0754804158000	-0.0985918031000
C64	-0.1513812310000	-4.8606561282000	0.6087889273000
H65	0.1068119323000	-5.8850888089000	0.8585385652000
C66	-1.3708619911000	-4.2936891448000	0.9789234770000
H67	-2.1185147269000	-4.8657597565000	1.5175614296000
C68	-1.6222722318000	-2.9647826746000	0.6615669921000
H69	-2.5537660321000	-2.4940103750000	0.9489314850000
C70	0.4400652385000	-2.7469547984000	-0.3777805816000
H71	1.1384073418000	-2.1030710643000	-0.8927008855000
O72	0.5666231086000	-0.2359440696000	-1.5674716416000
H73	0.4288537937000	0.3991372046000	-2.2888630975000

Catalyst 1 at Ru^V oxidation state

E(B3LYP-D3/LACV3P**++) (a.u.) = -2299.744762

Solvation energy (kcal mol^{-1}) = -64.807

Zero-point energy (kcal mol^{-1}) = 341.109

ΔH_{298} (kcal mol^{-1}) = 25.762

ΔS_{298} (cal K^{-1} mol^{-1}) = 242.811

Cartesian coordinates

Ru1	-1.0317985901000	-0.0997099496000	-0.6994082456000
O2	1.7368151357000	5.8591247736000	-0.6607525435000
O3	2.2947214074000	3.8083508318000	0.1564923484000
O4	4.1873892995000	1.8247591805000	-0.0493079608000
O5	5.2078965485000	-1.6202921619000	-0.1984191095000
O6	2.9717184893000	-3.5478728236000	-0.4661227549000
O7	2.3920649944000	-5.6819077746000	0.0581930526000
O8	0.9233044475000	0.0360111372000	0.2317640092000
O9	2.2259220446000	0.2749827583000	2.0557962073000
O10	-2.2979913660000	-0.3381153193000	-2.3844301455000
O11	-4.3346324412000	-0.6548821403000	-3.2948879490000
N12	-1.0938435386000	2.0227108694000	-0.8913634207000
N13	-0.8458781291000	-2.2100788767000	-0.4271081084000
N14	-3.2061347357000	-0.1205603991000	-0.0205832676000
N15	-1.2458119896000	0.1790977581000	1.5559792886000
C16	-4.1418347805000	-0.3193431911000	-0.9551892565000
C17	-5.5013284209000	-0.3715279908000	-0.6602491614000
H18	-6.1935278952000	-0.5403620304000	-1.4778745761000
C19	-5.8963850371000	-0.2020971941000	0.6638412426000
H20	-6.9456976724000	-0.2348775326000	0.9399980820000
C21	-4.9221745162000	0.0115124920000	1.6401328804000
H22	-5.2052248443000	0.1445497841000	2.6777493850000
C23	-3.5768335441000	0.0456493718000	1.2689720571000
C24	-2.4474190468000	0.2357291565000	2.1748224727000
C25	-2.5449965417000	0.4447730541000	3.5513625301000
H26	-3.5170729479000	0.4912761363000	4.0280620122000
C27	-1.3811068235000	0.5908810996000	4.3064056486000
H28	-1.4410177194000	0.7537477622000	5.3778590179000
C29	-0.1508596614000	0.5177754810000	3.6626620497000
H30	0.8010827371000	0.6099452339000	4.1736403239000
C31	-0.1334130278000	0.3112173535000	2.2857546403000
C32	-3.5941046865000	-0.4634221342000	-2.3478907741000
C33	1.1402115354000	0.2047235927000	1.5042858562000
C34	-2.1902063499000	2.7331980440000	-1.2276553156000
H35	-3.0907913454000	2.1721430303000	-1.4386860845000
C36	-2.1607046981000	4.1207185066000	-1.3151557249000
H37	-3.0614811909000	4.6554613838000	-1.5956253868000

C38	-0.9703536070000	4.7979352757000	-1.0463585212000
H39	-0.8983290966000	5.8798094541000	-1.1026504079000
C40	0.1575351252000	4.0526669918000	-0.7035378447000
C41	0.0641014593000	2.6650108488000	-0.6596676773000
H42	0.9336607782000	2.0579345788000	-0.4484247483000
C43	1.4775170699000	4.7039168174000	-0.4095911571000
C44	3.6981294846000	4.1257654749000	0.3004642247000
H45	3.8144271061000	4.9858015732000	0.9672015559000
H46	4.1017985260000	4.3787490016000	-0.6842705077000
C47	4.3538963540000	2.8841941514000	0.8693067397000
H48	5.4200264493000	3.1077997842000	1.0435708758000
H49	3.8979439502000	2.6312558684000	1.8400157349000
C50	4.8584568421000	0.6380252532000	0.3576088135000
H51	4.6043234371000	0.3795177950000	1.3927250168000
H52	5.9500252985000	0.7631152474000	0.2831083150000
C53	4.4173132090000	-0.4930252952000	-0.5608747324000
H54	3.3442352689000	-0.6811424074000	-0.4265961944000
H55	4.5910006523000	-0.2053220844000	-1.6092283915000
C56	5.1558083600000	-2.7367535521000	-1.0645311918000
H57	6.1844342308000	-3.0837587672000	-1.2323730261000
H58	4.7276923333000	-2.4766566128000	-2.0430340690000
C59	4.3825059631000	-3.8964685381000	-0.4633466770000
H60	4.6916012740000	-4.0787812671000	0.5700449510000
H61	4.5210490120000	-4.8148356836000	-1.0424689611000
C62	2.1055466258000	-4.5307917114000	-0.1897794301000
C63	0.6910553375000	-4.0309637663000	-0.2145299693000
C64	-0.3658816572000	-4.9130334513000	0.0164740312000
H65	-0.1495179974000	-5.9607932746000	0.2003890063000
C66	-1.6683147855000	-4.4166146168000	-0.0008261263000
H67	-2.5214275163000	-5.0667451282000	0.1592262848000
C68	-1.8731558997000	-3.0602927544000	-0.2253476329000
H69	-2.8693559003000	-2.6379448390000	-0.2441513250000
C70	0.4131186714000	-2.6879843941000	-0.4431934457000
H71	1.2062976709000	-1.9768840866000	-0.6219463620000
O72	0.0815325493000	-0.1849224163000	-2.0190796465000

Catalyst 2 at Ru^{II} oxidation state

E(B3LYP-D3/LACV3P**++) (a.u.) = -2153.000832

Solvation energy (kcal mol^{-1}) = -31.225

Zero-point energy (kcal mol^{-1}) = 368.484

ΔH_{298} (kcal mol^{-1}) = 25.287

ΔS_{298} (cal K^{-1} mol^{-1}) = 238.549

Cartesian coordinates

Ru1	1.2148238159000	0.0990994837000	-0.3392101110000
O2	-2.0848356568000	5.8536598131000	-0.4221859723000
O3	-2.7185648002000	3.6855850427000	-0.6531769995000
O4	-2.3417346434000	-3.7933909852000	-0.3053703657000
O5	-1.5987107540000	-5.8744530648000	-0.8315845030000
O6	2.1840432676000	0.3078047163000	-2.3046780588000
O7	4.2857017339000	0.4799659379000	-3.1581460477000
O8	-1.0487107362000	-0.0112583814000	0.0215664761000
O9	-2.4758525847000	-0.2220334626000	1.7709373952000
N10	1.0240267025000	2.2146999822000	-0.2945037923000
N11	1.2277843940000	-2.0088909649000	-0.6354115651000
N12	1.0461757315000	-0.0787609953000	1.6364387085000
N13	3.1201150549000	0.1315966077000	0.1461560590000
C14	-0.1518087264000	-0.1715231082000	2.2326396368000
C15	-0.2368542562000	-0.2990436834000	3.6156948959000
H16	-1.2211484149000	-0.3726880441000	4.0642488898000
C17	0.9442407687000	-0.3269354121000	4.3634267857000
H18	0.9042218081000	-0.4255738091000	5.4438181627000
C19	2.1840518206000	-0.2282365453000	3.7233556094000
H20	3.1051808811000	-0.2489172676000	4.2961039150000
C21	2.2220685919000	-0.1024903091000	2.3340075992000
C22	3.4180876981000	0.0165058087000	1.4740843226000
C23	4.7598564455000	0.0267062408000	1.8592665145000
H24	5.0275472602000	-0.0639642085000	2.9067337339000
C25	5.7506098008000	0.1545457654000	0.8799733819000
H26	6.7954632610000	0.1621433940000	1.1749317713000
C27	5.3996322513000	0.2716235559000	-0.4700861854000
H28	6.1294936935000	0.3723536333000	-1.2657568179000
C29	4.0519598131000	0.2565442493000	-0.8132985702000
C30	-1.3502945720000	-0.1296309516000	1.2790585264000
C31	3.4757123055000	0.3627577100000	-2.2410908662000
C32	2.0755022902000	3.0592881756000	-0.2515246525000
H33	3.0567426490000	2.6064498085000	-0.1884276850000
C34	1.9325519004000	4.4420625487000	-0.2906524804000
H35	2.8158150538000	5.0708189026000	-0.2539521786000
C36	0.6562293217000	4.9892911959000	-0.3764226834000
H37	0.4826023019000	6.0602124018000	-0.3990231449000

C38	-0.4333919417000	4.1187773460000	-0.4367433308000
C39	-0.2116164408000	2.7432381244000	-0.3993722216000
H40	-1.0288768049000	2.0358763063000	-0.4459221094000
C41	-1.8216786018000	4.6695595766000	-0.5053763127000
C42	-4.1221325241000	4.0371207685000	-0.6310739637000
H43	-4.2850987475000	4.8875175271000	-1.3005771624000
H44	-4.3732817456000	4.3542764911000	0.3884608652000
C45	-4.9097759573000	2.8049700630000	-1.0468975289000
H46	-5.9728171489000	3.0823709400000	-1.0079175321000
H47	-4.6877646298000	2.5749360703000	-2.0969583377000
C48	-5.7253496897000	0.4828083042000	-0.4191527434000
H49	-5.7727607635000	0.2494532576000	-1.4935054543000
H50	-6.7064457881000	0.9089944819000	-0.1647994721000
C51	-5.5234192795000	-0.8190164036000	0.3776186200000
H52	-5.1647044078000	-0.5768443543000	1.3858865222000
H53	-6.4981329605000	-1.3138256675000	0.4969484031000
C54	-4.4650633523000	-3.1174326569000	0.5535569419000
H55	-5.4766367024000	-3.4940142919000	0.7617307107000
H56	-3.9978926881000	-2.9048446574000	1.5231600604000
C57	-3.7034836691000	-4.2463298032000	-0.1237235317000
H58	-4.1173952347000	-4.4897457209000	-1.1097777756000
H59	-3.6912657274000	-5.1633497351000	0.4743002501000
C60	-1.4067152294000	-4.6882982086000	-0.6517066202000
C61	-0.0711281653000	-4.0267936420000	-0.7714299031000
C62	1.0698536007000	-4.7728110721000	-1.0701910258000
H63	0.9820561005000	-5.8431565163000	-1.2265459368000
C64	2.2875278058000	-4.1031082699000	-1.1589927879000
H65	3.2044876205000	-4.6308483312000	-1.3986336048000
C66	2.3291706101000	-2.7297296953000	-0.9370840621000
H67	3.2621332070000	-2.1850613818000	-1.0046411454000
C68	0.0476596381000	-2.6539026364000	-0.5720537168000
H69	-0.8182762356000	-2.0405586016000	-0.3707341477000
C70	-4.6599943093000	1.5624800415000	-0.1757284060000
C71	-4.5496203651000	-1.8182961860000	-0.2631211298000
H72	-4.6761310361000	1.8447636071000	0.8860969697000
H73	-3.6574570194000	1.1628064628000	-0.3583689456000
H74	-4.8870471573000	-2.0522806166000	-1.2843260570000
H75	-3.5550217120000	-1.3680610540000	-0.3405622685000

Catalyst 2 at Ru^{III} oxidation state

E(B3LYP-D3/LACV3P**++) (a.u.) = -2229.252760

Solvation energy (kcal mol^{-1}) = -59.467

Zero-point energy (kcal mol^{-1}) = 385.746

ΔH_{298} (kcal mol^{-1}) = 26.723

ΔS_{298} (cal K^{-1} mol^{-1}) = 246.693

Cartesian coordinates

Ru1	1.2605563368000	0.0851217409000	-0.5643486238000
O2	-2.0512296650000	5.8367689660000	-0.2257743752000
O3	-2.7132885983000	3.6727029796000	-0.4632903660000
O4	-2.2921263486000	-3.7767799792000	-0.2588315174000
O5	-1.5461407590000	-5.8715080661000	-0.7343138830000
O6	2.4073465168000	0.2833548521000	-2.2420790463000
O7	4.5408266631000	0.4630158867000	-2.9726652711000
O8	-1.1320529330000	-0.0038571047000	0.0335521131000
O9	-2.5687862853000	-0.2335901949000	1.7725822869000
N10	1.0600246017000	2.2194051800000	-0.5258703688000
N11	1.3055107123000	-2.0327458685000	-0.7277488749000
N12	0.9600073618000	-0.0804540926000	1.5974090193000
N13	3.1192123419000	0.1212992320000	0.2360072463000
C14	-0.2376926766000	-0.1736871631000	2.1913769341000
C15	-0.3656877461000	-0.3000804991000	3.5743606604000
H16	-1.3674896932000	-0.3717408283000	3.9827527967000
C17	0.7831722845000	-0.3275708453000	4.3575887157000
H18	0.7176583762000	-0.4241720744000	5.4365926912000
C19	2.0292826725000	-0.2284747573000	3.7371470027000
H20	2.9384144180000	-0.2468663794000	4.3264169899000
C21	2.0906141736000	-0.1057106796000	2.3493278923000
C22	3.3288240083000	0.0051537988000	1.5673675581000
C23	4.6384191612000	-0.0025275867000	2.0563797612000
H24	4.8195060139000	-0.0966874354000	3.1207022350000
C25	5.7061654536000	0.1085519912000	1.1654623681000
H26	6.7248053828000	0.1010052702000	1.5396532240000
C27	5.4572320644000	0.2262943283000	-0.2041897545000
H28	6.2418025837000	0.3143909275000	-0.9479119320000
C29	4.1390654109000	0.2263377167000	-0.6382858992000
C30	-1.4511190370000	-0.1364002727000	1.2827611424000
C31	3.7145001775000	0.3372418280000	-2.0916970339000
C32	2.1036686717000	3.0645079033000	-0.6587549542000
H33	3.0869095381000	2.6205683483000	-0.7443705418000
C34	1.9475560831000	4.4456760229000	-0.6859783784000
H35	2.8217680571000	5.0790819062000	-0.7891082728000
C36	0.6678937196000	4.9869197841000	-0.5873184993000
H37	0.4886505403000	6.0573457598000	-0.5989534532000

C38	-0.4154267968000	4.1141808886000	-0.4756524571000
C39	-0.1798473793000	2.7427737663000	-0.4428666024000
H40	-0.9959834419000	2.0429081376000	-0.3340537437000
C41	-1.8108467808000	4.6573410625000	-0.3717662666000
C42	-4.1180013710000	4.0283662752000	-0.3048580304000
H43	-4.3116536245000	4.9390733155000	-0.8781722807000
H44	-4.2831346011000	4.2481622218000	0.7560838944000
C45	-4.9543104730000	2.8506195153000	-0.7768452049000
H46	-6.0050968164000	3.1458726945000	-0.6535381079000
H47	-4.8000848141000	2.7087300853000	-1.8544980961000
C48	-5.7585180646000	0.4747117885000	-0.3352332464000
H49	-5.7965892757000	0.2936651383000	-1.4197106084000
H50	-6.7391056124000	0.8922123004000	-0.0696314474000
C51	-5.5738032354000	-0.8624540953000	0.4055162877000
H52	-5.2513184847000	-0.6629230939000	1.4352150912000
H53	-6.5485043076000	-1.3640381175000	0.4750995470000
C54	-4.4483765987000	-3.1260261571000	0.5823424888000
H55	-5.4468979990000	-3.5306482253000	0.7967882398000
H56	-3.9899106227000	-2.8910923420000	1.5510200541000
C57	-3.6601699193000	-4.2436740700000	-0.0797502377000
H58	-4.0569534371000	-4.5033090658000	-1.0677287336000
H59	-3.6359474686000	-5.1533802272000	0.5273195078000
C60	-1.3620979519000	-4.6828243658000	-0.5847228976000
C61	-0.0182746248000	-4.0337003614000	-0.7409408964000
C62	1.1097492690000	-4.8000220095000	-1.0365276036000
H63	1.0067192577000	-5.8745302128000	-1.1513161505000
C64	2.3347832647000	-4.1507675880000	-1.1810607985000
H65	3.2381200308000	-4.7008309445000	-1.4209598995000
C66	2.3960457900000	-2.7700108026000	-1.0241048020000
H67	3.3317639128000	-2.2385591034000	-1.1429278552000
C68	0.1198701644000	-2.6573920962000	-0.6005750333000
H69	-0.7379216975000	-2.0356958659000	-0.3944196286000
C70	-4.6914340253000	1.5362279136000	-0.0249923887000
C71	-4.5806771211000	-1.8374726735000	-0.2447843947000
H72	-4.6834066683000	1.7267137160000	1.0568343511000
H73	-3.6918592020000	1.1571945480000	-0.2627582696000
H74	-4.9253256739000	-2.0867397600000	-1.2593025500000
H75	-3.5984144766000	-1.3634524196000	-0.3412910619000
O76	-0.2138747831000	-0.0604686387000	-2.2335673163000
H77	-0.0237776554000	0.5628438063000	-2.9484779422000
H78	-1.0814708479000	0.1271538434000	-1.8296672736000

Catalyst 2 at Ru^{IV} oxidation state

E(B3LYP-D3/LACV3P**++) (a.u.) = -2228.604805

Solvation energy (kcal mol^{-1}) = -61.663

Zero-point energy (kcal mol^{-1}) = 378.801

ΔH_{298} (kcal mol^{-1}) = 26.237

ΔS_{298} (cal K^{-1} mol^{-1}) = 244.070

Cartesian coordinates

Ru1	1.1554950282000	0.0739293305000	-0.5569542746000
O2	-2.1646086834000	5.8089244698000	-0.2364362789000
O3	-2.8282731258000	3.6582962948000	-0.5634587368000
O4	-2.3782995138000	-3.8038490112000	-0.3622630936000
O5	-1.6250660315000	-5.8918946787000	-0.8606183492000
O6	2.4636344392000	0.3973349008000	-2.1672470316000
O7	4.5640902049000	0.4396799070000	-2.9891343823000
O8	-0.8743008064000	-0.0632783529000	0.1088106084000
O9	-2.3830340090000	-0.0856950177000	1.7822296656000
N10	0.9359307837000	2.1897054440000	-0.5133234654000
N11	1.2157765964000	-2.0529408960000	-0.7201962599000
N12	1.1207808697000	0.0104097037000	1.6451829391000
N13	3.1980885817000	0.0582252107000	0.2170898573000
C14	-0.0570558070000	-0.0243417827000	2.2801430604000
C15	-0.1643067026000	-0.0757998835000	3.6655617840000
H16	-1.1593370844000	-0.0958754581000	4.0964348352000
C17	1.0036513365000	-0.1116653881000	4.4214368995000
H18	0.9650419599000	-0.1536366893000	5.5051812621000
C19	2.2323037003000	-0.1062895522000	3.7623939568000
H20	3.1570205316000	-0.1488789221000	4.3253497307000
C21	2.2635476922000	-0.0457465218000	2.3679348172000
C22	3.4640364252000	-0.0347127128000	1.5414150175000
C23	4.7805780576000	-0.0976756726000	2.0031647685000
H24	4.9783456560000	-0.1772949747000	3.0657720336000
C25	5.8326111050000	-0.0551060216000	1.0887030760000
H26	6.8594631584000	-0.1083405906000	1.4362494759000
C27	5.5437017439000	0.0713538513000	-0.2681737197000
H28	6.3018547651000	0.1348239904000	-1.0413495979000
C29	4.2108728609000	0.1282296133000	-0.6582367954000
C30	-1.2386727684000	-0.0531731180000	1.3630944995000
C31	3.7658290113000	0.3269866377000	-2.0788749570000
C32	1.9933755991000	3.0279019945000	-0.5007269149000
H33	2.9750098487000	2.5738635885000	-0.5234772866000
C34	1.8433782420000	4.4081282260000	-0.4614624539000
H35	2.7235033972000	5.0417155567000	-0.4503925753000
C36	0.5592094145000	4.9487862082000	-0.4356092538000
H37	0.3824186212000	6.0187176868000	-0.3875729857000

C38	-0.5327576131000	4.0812687108000	-0.4739039710000
C39	-0.3076511082000	2.7073352084000	-0.5216706178000
H40	-1.1282336308000	2.0036772985000	-0.5555492848000
C41	-1.9270464476000	4.6331341157000	-0.4149034012000
C42	-4.2334469639000	4.0131847253000	-0.4199701777000
H43	-4.4347416220000	4.8916767670000	-1.0396352460000
H44	-4.3987468010000	4.2875168201000	0.6281801037000
C45	-5.0556618074000	2.8039795037000	-0.8295573496000
H46	-6.1098886476000	3.0853576610000	-0.7041921542000
H47	-4.9115186481000	2.6164921576000	-1.9011623892000
C48	-5.8272153974000	0.4501237669000	-0.2686693897000
H49	-5.9169926142000	0.2574147619000	-1.3477294366000
H50	-6.7972456091000	0.8610035213000	0.0421933643000
C51	-5.5951063049000	-0.8805508671000	0.4683688549000
H52	-5.2539567315000	-0.6775487240000	1.4924405082000
H53	-6.5591542628000	-1.3988656414000	0.5605811728000
C54	-4.5177466515000	-3.1790510625000	0.5261013626000
H55	-5.5293677036000	-3.5710410627000	0.6975521177000
H56	-4.0686714058000	-3.0301154148000	1.5165153678000
C57	-3.7487950996000	-4.2652513103000	-0.2073780704000
H58	-4.1530028811000	-4.4533099671000	-1.2084776549000
H59	-3.7356216251000	-5.2122535798000	0.3399346734000
C60	-1.4453392958000	-4.7051879484000	-0.6912850885000
C61	-0.0993202892000	-4.0529447373000	-0.7994188665000
C62	1.0474748684000	-4.8233605847000	-0.9889979343000
H63	0.9546568320000	-5.9008953480000	-1.0826945405000
C64	2.2803195409000	-4.1752394085000	-1.0524790055000
H65	3.2003700848000	-4.7275173338000	-1.2103021906000
C66	2.3279323993000	-2.7935153006000	-0.9096639717000
H67	3.2711147249000	-2.2651800403000	-0.9534427350000
C68	0.0219148985000	-2.6708257553000	-0.6937710937000
H69	-0.8492888507000	-2.0423365529000	-0.5870833127000
C70	-4.7580418474000	1.5261593367000	-0.0279279337000
C71	-4.6027560104000	-1.8344237719000	-0.2138006219000
H72	-4.7211121340000	1.7612865625000	1.0449338935000
H73	-3.7636659365000	1.1441101534000	-0.2832491846000
H74	-4.9251835102000	-2.0118141686000	-1.2501559395000
H75	-3.6093598321000	-1.3751942322000	-0.2610293544000
O76	0.0818940817000	-0.0478646534000	-2.1206476594000
H77	0.1001459967000	0.8031557068000	-2.5903852860000

Catalyst 2 at Ru^{V} oxidation state

E(B3LYP-D3/LACV3P**++) (a.u.) = -2227.945247

Solvation energy (kcal mol^{-1}) = -60.999

Zero-point energy (kcal mol^{-1}) = 371.570

ΔH_{298} (kcal mol^{-1}) = 26.015

ΔS_{298} (cal K^{-1} mol^{-1}) = 243.514

Cartesian coordinates

Ru1	1.1298319057000	0.0633476002000	-0.7704515454000
O2	-2.0624971752000	5.8077753289000	-0.0846274640000
O3	-2.7382986055000	3.6685570855000	-0.4642300800000
O4	-2.3753112570000	-3.8373904286000	-0.3176947688000
O5	-1.5821617405000	-5.9531364941000	-0.5971554164000
O6	2.5766496362000	0.2038169891000	-2.3123815668000
O7	4.7128375014000	0.3552508369000	-3.0128457877000
O8	-0.9290838745000	-0.0451931354000	-0.0518394497000
O9	-2.4201411224000	-0.2613175807000	1.6255145348000
N10	1.0052734161000	2.1908772789000	-0.6231867640000
N11	1.1841539289000	-2.0698337027000	-0.8548918346000
N12	1.0850136385000	-0.0804840016000	1.5090849425000
N13	3.2161544984000	0.0918965986000	0.1447081719000
C14	-0.1018844020000	-0.1700767983000	2.1193986125000
C15	-0.2385217773000	-0.2672896412000	3.5017203058000
H16	-1.2414337513000	-0.3379114652000	3.9079159129000
C17	0.9111906449000	-0.2680077891000	4.2833525701000
H18	0.8506432591000	-0.3405808141000	5.3646179115000
C19	2.1518301610000	-0.1738189214000	3.6530627108000
H20	3.0645019536000	-0.1724005169000	4.2372388687000
C21	2.2096041498000	-0.0818248565000	2.2614508168000
C22	3.4367521515000	0.0206720494000	1.4759232491000
C23	4.7343240922000	0.0490077648000	1.9911109224000
H24	4.8986999544000	-0.0083861291000	3.0604409841000
C25	5.8167494221000	0.1530411158000	1.1169321389000
H26	6.8299249166000	0.1765006709000	1.5058461328000
C27	5.5755381678000	0.2258793937000	-0.2521679792000
H28	6.3589504573000	0.3083085137000	-0.9974385277000
C29	4.2553228212000	0.1914005786000	-0.6912822278000
C30	-1.2813289963000	-0.1613020465000	1.1964813883000
C31	3.8673641354000	0.2611543998000	-2.1423033923000
C32	2.0756067367000	3.0118359372000	-0.5894138775000
H33	3.0509739221000	2.5515109786000	-0.6678305777000
C34	1.9384567272000	4.3886023456000	-0.4610861963000
H35	2.8247996703000	5.0131063202000	-0.4356034309000
C36	0.6606090210000	4.9381451962000	-0.3658451987000
H37	0.4976046169000	6.0048298803000	-0.2468553861000

C38	-0.4421266635000	4.0857564606000	-0.4315993665000
C39	-0.2324488365000	2.7174502370000	-0.5736715871000
H40	-1.0611955639000	2.0266720047000	-0.6375095591000
C41	-1.8333300469000	4.6385552188000	-0.3098441273000
C42	-4.1448186047000	4.0142436594000	-0.3089455579000
H43	-4.3429657655000	4.9234777273000	-0.8835019216000
H44	-4.3160714376000	4.2332243092000	0.7512336635000
C45	-4.9664894458000	2.8284338618000	-0.7868272841000
H46	-6.0210037191000	3.1165112967000	-0.6796786532000
H47	-4.7935726855000	2.6821606170000	-1.8605467007000
C48	-5.7652540512000	0.4517949502000	-0.3481009827000
H49	-5.7844376494000	0.2669999179000	-1.4319428994000
H50	-6.7507655204000	0.8678758479000	-0.0993479310000
C51	-5.5911127225000	-0.8841482304000	0.3987065887000
H52	-5.2650162120000	-0.6847095012000	1.4282336531000
H53	-6.5708520760000	-1.3747244899000	0.4753139228000
C54	-4.5070819557000	-3.1706217924000	0.5651164146000
H55	-5.5128047715000	-3.5577760288000	0.7779361014000
H56	-4.0424187480000	-2.9552624069000	1.5357570984000
C57	-3.7417349515000	-4.2971965452000	-0.1088209935000
H58	-4.1576294410000	-4.5525579866000	-1.0898548286000
H59	-3.7102754260000	-5.2060509652000	0.4987351270000
C60	-1.4289516821000	-4.7519121571000	-0.5532674752000
C61	-0.0946846147000	-4.0881654729000	-0.7364966818000
C62	1.0668958639000	-4.8487334689000	-0.8750869592000
H63	0.9949300667000	-5.9319932015000	-0.8687902177000
C64	2.2857543217000	-4.1867964161000	-1.0210939246000
H65	3.2131878697000	-4.7348081110000	-1.1459710725000
C66	2.3104095983000	-2.7971751363000	-1.0057427820000
H67	3.2361687962000	-2.2504919818000	-1.1206190368000
C68	0.0009575934000	-2.7009679158000	-0.7506860623000
H69	-0.8802579597000	-2.0806133009000	-0.6768854919000
C70	-4.7056951071000	1.5177819542000	-0.0262951152000
C71	-4.6131681074000	-1.8735682052000	-0.2528871883000
H72	-4.7145725787000	1.7129984259000	1.0553561015000
H73	-3.7028315530000	1.1413218391000	-0.2535992896000
H74	-4.9584073968000	-2.1098484949000	-1.2698471415000
H75	-3.6231595123000	-1.4153695610000	-0.3460621979000
O76	0.1651116905000	0.1047754907000	-2.2012429292000

Catalyst 3 at Ru^{II} oxidation state

E(B3LYP-D3/LACV3P**++) (a.u.) = -2378.688953

Solvation energy (kcal mol^{-1}) = -35.636

Zero-point energy (kcal mol^{-1}) = 376.923

ΔH_{298} (kcal mol^{-1}) = 27.026

ΔS_{298} (cal K^{-1} mol^{-1}) = 253.155

Cartesian coordinates

Ru1	-2.4910639864000	-3.6138229637000	-7.4354107334000
O2	-2.3602453076000	-1.1505703370000	-10.8462129644000
O3	-1.6690049447000	-2.6093490811000	-9.2494539065000
O4	-1.2973429227000	-4.9388582690000	-6.1583658615000
O5	-1.4965258049000	-5.9694109546000	-4.1436206990000
O6	2.6245874065000	0.0865855635000	-5.8206183189000
O7	2.3934224605000	-1.9695359467000	-6.7624252183000
O8	3.0110958937000	-2.1557423666000	-9.6875353169000
O9	1.2188117826000	-2.7943634464000	-11.9527602351000
O10	-1.2232389851000	-3.2809364335000	-13.6806613488000
O11	-3.4164090162000	-4.7438778583000	-12.5678575531000
O12	-3.7527156598000	-6.9389214568000	-13.0455782165000
N16	-4.0151499513000	-2.5230288409000	-8.0547122258000
N17	-3.7593064975000	-4.0062390077000	-5.9918795168000
N18	-1.6195270491000	-2.0303343971000	-6.3843225814000
N19	-3.0686013021000	-5.2899073838000	-8.6016856976000
C20	-2.5239180869000	-1.8291880823000	-9.8268728894000
C21	-3.9043322880000	-1.7633717730000	-9.1547338258000
C22	-4.9860026001000	-1.0008430299000	-9.5822724410000
H23	-4.8715243164000	-0.3996183136000	-10.4773070609000
C24	-6.1704568012000	-1.0407960515000	-8.8383607591000
H25	-7.0304076046000	-0.4572489553000	-9.1523069287000
C26	-6.2550865631000	-1.8258209322000	-7.6825103546000
H27	-7.1685900351000	-1.8502487330000	-7.0973840776000
C28	-5.1471589631000	-2.5769221754000	-7.2881691214000
C29	-4.9929580084000	-3.4240387435000	-6.0835554085000
C30	-5.9038034506000	-3.6380613223000	-5.0479723106000
H31	-6.8923859015000	-3.1931065105000	-5.0879425137000
C32	-5.5207323126000	-4.4243358173000	-3.9552239913000
H33	-6.2253242517000	-4.5920988101000	-3.1464707884000
C34	-4.2386195535000	-4.9834785253000	-3.8962835293000
H35	-3.8956897282000	-5.5888019160000	-3.0645253611000
C36	-3.3610658001000	-4.7508480979000	-4.9499397962000
C37	-1.9149649634000	-5.2774001704000	-5.0670196570000
C38	-3.2982822533000	-6.5040265420000	-8.0565657535000
H39	-3.1922372244000	-6.5752707797000	-6.9822755652000
C40	-3.6325800797000	-7.6147056886000	-8.8249582323000

H41	-3.8008245007000	-8.5683766350000	-8.3362927057000
C42	-3.7362803944000	-7.4813376755000	-10.2071478222000
H43	-3.9894883309000	-8.3154246328000	-10.8535758111000
C44	-3.4941673742000	-6.2278277689000	-10.7715147867000
C45	-3.1553372950000	-5.1644305625000	-9.9384195267000
H46	-2.9117466844000	-4.1999059283000	-10.3541173340000
C47	-3.5718008112000	-6.0378369477000	-12.2519160912000
C48	-3.3052077605000	-4.3787609765000	-13.9583813019000
H49	-4.3116433735000	-4.2965351466000	-14.3842032991000
H50	-2.7537289500000	-5.1539931798000	-14.4965521136000
C51	-2.5761952881000	-3.0519969341000	-14.0021869211000
H52	-2.6819787837000	-2.6266406037000	-15.0175982665000
H53	-3.0386945051000	-2.3491552868000	-13.2904640045000
C54	-0.4646062445000	-2.0791307649000	-13.6353622474000
H55	-0.9011483908000	-1.3849426640000	-12.9040692238000
H56	-0.4793587577000	-1.5859420844000	-14.6252311825000
C57	0.9855783962000	-2.4086658134000	-13.2897442831000
H58	1.3085669378000	-3.2495430073000	-13.9134486130000
H59	1.6034291493000	-1.5308221056000	-13.5535810046000
C60	1.0604693164000	-1.7346503459000	-11.0161114537000
H61	0.0058905829000	-1.4612640670000	-10.9027455444000
H62	1.6271205475000	-0.8445495095000	-11.3415454131000
C63	1.5817826324000	-2.1753369991000	-9.6671322883000
H64	1.1879024059000	-1.4891970943000	-8.9064668546000
H65	1.2003981825000	-3.1807788232000	-9.4376691563000
C66	3.6592800921000	-2.8007873947000	-8.6155078986000
H67	4.6901579873000	-2.9873013348000	-8.9413931153000
H68	3.1951109477000	-3.7741509909000	-8.3872592192000
C69	3.7193509573000	-1.9665830423000	-7.3398129328000
H70	4.0203866303000	-0.9371886185000	-7.5504401523000
H71	4.4134628667000	-2.4030182982000	-6.6123071786000
C72	1.9470428723000	-0.8692369858000	-6.1379113602000
C73	0.4681421120000	-0.9468757546000	-5.9291037608000
C74	-0.1971799268000	0.0941720365000	-5.2763731092000
H75	0.3802763922000	0.9218087806000	-4.8775199433000
C76	-1.5846146609000	0.0398012496000	-5.1744435604000
H77	-2.1438698271000	0.8231322289000	-4.6740874786000
C78	-2.2621724794000	-1.0315324314000	-5.7492414289000
H79	-3.3441778305000	-1.1002836244000	-5.7190287583000
C80	-0.2760888477000	-2.0012678177000	-6.4532060498000
H81	0.1959344403000	-2.8347637720000	-6.9543912001000

Catalyst 3 at Ru^{III} oxidation state

E(B3LYP-D3/LACV3P**++) (a.u.) = -2454.951595

Solvation energy (kcal mol^{-1}) = -60.720

Zero-point energy (kcal mol^{-1}) = 394.623

ΔH_{298} (kcal mol^{-1}) = 28.318

ΔS_{298} (cal K^{-1} mol^{-1}) = 260.234

Cartesian coordinates

Ru1	5.4900994035000	4.2791906551000	16.4611581755000
O2	3.5111083597000	5.0862314529000	12.8124888399000
O3	5.0840585476000	4.9810976539000	14.4334603002000
O4	7.2288239938000	4.3222772344000	17.6503141737000
O5	7.9551832293000	4.1143598999000	19.7841566744000
O6	6.7678236279000	10.6634864039000	16.1398021065000
O7	8.0454010563000	8.7983619795000	15.8575182558000
O8	8.3959801119000	8.1871515628000	12.8805397644000
O9	7.4425106608000	5.3823605138000	12.4189303433000
O10	5.9309495132000	3.0459799929000	11.0812109119000
O11	4.5516433698000	1.0464788167000	12.2603635712000
O12	6.1453552632000	-0.5764415876000	12.0684111850000
O13	7.5259458145000	4.8900725756000	15.1416041338000
N16	3.4911204265000	3.8890717950000	16.1233005564000
N17	4.8291594095000	3.6146539340000	18.2566300948000
N18	5.1386334065000	6.2827918319000	17.0390710667000
N19	5.9661376858000	2.3031498331000	15.8282589284000
C20	3.8984621743000	4.7977672215000	13.9391522730000
C21	2.9453182340000	4.1475228385000	14.9272923033000
C22	1.6128236992000	3.8425483403000	14.6685243507000
H23	1.2175823375000	4.0705094743000	13.6848313394000
C24	0.8507835232000	3.2643404766000	15.6837198854000
H25	-0.1903331175000	3.0100168434000	15.5125616651000
C26	1.4283132227000	3.0193601376000	16.9330756176000
H27	0.8435598620000	2.5824763797000	17.7344439399000
C28	2.7684149905000	3.3461276321000	17.1376030863000
C29	3.5388165855000	3.2100712255000	18.3832777229000
C30	3.0808720908000	2.7678854368000	19.6246835402000
H31	2.0542698575000	2.4389996261000	19.7410470167000
C32	3.9579183827000	2.7624488750000	20.7131834655000
H33	3.6090258989000	2.4233107457000	21.6833702191000
C34	5.2757970672000	3.1936995995000	20.5494666295000
H35	6.0002105645000	3.2128551888000	21.3564888582000
C36	5.6847319815000	3.6142618473000	19.2890656525000
C37	7.0925481346000	4.0638966112000	18.9280204801000
C38	6.5981635916000	1.4240764285000	16.6354854763000
H39	6.8640412779000	1.7766203884000	17.6227268596000

C40	6.9242308465000	0.1371458478000	16.2218251984000
H41	7.4441894592000	-0.5267796086000	16.9038484390000
C42	6.5848777200000	-0.2674746273000	14.9327515523000
H43	6.8308870981000	-1.2546905701000	14.5543623029000
C44	5.9150841636000	0.6367076532000	14.1080091118000
C45	5.6419482624000	1.9167895334000	14.5821535108000
H46	5.2007391099000	2.6545144493000	13.9291212251000
C47	5.5685775780000	0.2806170781000	12.6981150417000
C48	4.4139022213000	1.2352530522000	10.8326510350000
H49	3.4001715449000	0.9367544515000	10.5516619315000
H50	5.1397650131000	0.6034559681000	10.3168392729000
C51	4.6532862985000	2.7088574969000	10.5575179840000
H52	4.6088497701000	2.8619999896000	9.4674851937000
H53	3.8807924370000	3.3344125447000	11.0272956647000
C54	6.4945779132000	4.1879108911000	10.4524794634000
H55	5.7527572365000	4.9958357484000	10.3826344057000
H56	6.8048389821000	3.9357206920000	9.4244637724000
C57	7.7213106474000	4.6628972180000	11.2214478866000
H58	8.3202544455000	3.7949245371000	11.5143762260000
H59	8.3290648394000	5.2922466772000	10.5539775327000
C60	6.7079325473000	6.6044688322000	12.2245369472000
H61	5.6352750200000	6.4035611340000	12.3177794192000
H62	6.9177221760000	7.0118821366000	11.2266163239000
C63	7.1475659470000	7.6138948713000	13.2650442121000
H64	6.3827131016000	8.4010300778000	13.3495733258000
H65	7.2206668615000	7.1110355763000	14.2295873745000
C66	9.3558154889000	8.3683245794000	13.9020158022000
H67	10.2710451717000	8.6927483635000	13.3955204048000
H68	9.5690871010000	7.4191886776000	14.4200560910000
C69	8.9880400220000	9.4194491607000	14.9465068143000
H70	8.5331247653000	10.3042983349000	14.4947463325000
H71	9.8654303486000	9.7185999362000	15.5286976270000
C72	6.9599561442000	9.4737454590000	16.2623192028000
C73	5.9457051834000	8.5269721208000	16.8332939187000
C74	4.7577713254000	9.0191225402000	17.3753905988000
H75	4.6178562626000	10.0923581080000	17.4585007070000
C76	3.7769922265000	8.1122018751000	17.7748938841000
H77	2.8424749600000	8.4488684212000	18.2102460791000
C78	3.9935407395000	6.7539728287000	17.5733086244000
H79	3.2375169456000	6.0200018807000	17.8279377130000
C80	6.1132470934000	7.1484050658000	16.7111565654000
H81	7.0196514045000	6.7148998094000	16.3122583787000
H80	8.3263333187000	4.4498103742000	15.4520160576000
H82	7.4833021520000	4.8675424024000	14.1576997612000

Catalyst 3 at Ru^{IV} oxidation state

E(B3LYP-D3/LACV3P**++) (a.u.) = -2454.291470

Solvation energy (kcal mol^{-1}) = -67.178

Zero-point energy (kcal mol^{-1}) = 387.375

ΔH_{298} (kcal mol^{-1}) = 27.935

ΔS_{298} (cal K^{-1} mol^{-1}) = 257.524

Cartesian coordinates

Ru1	5.6380195787000	4.2496329727000	16.5116286272000
O2	3.6813258980000	4.8554906449000	12.8231369583000
O3	5.1213723039000	4.9035426278000	14.5598161488000
O4	7.1330023942000	3.9899364945000	17.9595518376000
O5	7.6715019614000	4.1642960576000	20.1456977805000
O6	6.8945731686000	10.7099805693000	16.2329980163000
O7	8.1255269709000	8.8517422042000	15.7607982882000
O8	8.3802214492000	8.1945736702000	12.8536789660000
O9	7.4467147288000	5.6894732741000	11.5507272354000
O10	5.9085322433000	3.3327957417000	10.4482474045000
O11	5.1451015357000	1.2442082090000	12.1194386663000
O12	6.0431878230000	-0.8406717769000	12.2626337307000
O13	7.2696387559000	4.7860855225000	15.6665034988000
N16	3.5311031909000	3.7899556163000	16.1595707986000
N17	4.6821034480000	3.6965059450000	18.3854974999000
N18	5.3261062768000	6.2882500326000	17.0652114921000
N19	6.0215716832000	2.2451783726000	15.9071947999000
C20	3.9921377423000	4.6125486818000	13.9785908891000
C21	3.0409545277000	3.9401395729000	14.9233339844000
C22	1.7464097078000	3.5632464332000	14.5833291389000
H23	1.4254357231000	3.7199045993000	13.5592792382000
C24	0.9297518751000	3.0222211914000	15.5727767916000
H25	-0.0846015611000	2.7096219493000	15.3463210809000
C26	1.4251654019000	2.9072627148000	16.8723734657000
H27	0.8021661998000	2.5150221184000	17.6679678992000
C28	2.7348278982000	3.3059682586000	17.1423999469000
C29	3.3874649480000	3.3047256755000	18.4468852356000
C30	2.7976171544000	2.9850588513000	19.6707130785000
H31	1.7616264633000	2.6695458438000	19.7086325811000
C32	3.5512860447000	3.0869315509000	20.8408281667000
H33	3.1006001912000	2.8521465852000	21.7999044905000
C34	4.8860400126000	3.4776293518000	20.7574721447000
H35	5.5422025243000	3.5576910971000	21.6174564572000
C36	5.4164903396000	3.7557709247000	19.5025789153000
C37	6.8707801677000	4.0257110443000	19.2437779920000
C38	6.3513947570000	1.2910549384000	16.8053142715000
H39	6.4991208565000	1.6232930388000	17.8240036781000

C40	6.5184629491000	-0.0385537228000	16.4377544925000
H41	6.7942749029000	-0.7702241334000	17.1892669136000
C42	6.3254070287000	-0.4036869147000	15.1064379511000
H43	6.4349967416000	-1.4288139495000	14.7661131660000
C44	5.9826006422000	0.5850826576000	14.1839072555000
C45	5.8661371375000	1.9051049110000	14.6143869798000
H46	5.6665834132000	2.7009945688000	13.9094860885000
C47	5.7419885298000	0.2273959715000	12.7477272893000
C48	4.9871834803000	1.1692400243000	10.6810521232000
H49	4.1462220004000	0.5078235352000	10.4487774272000
H50	5.8997218080000	0.7564972397000	10.2437585694000
C51	4.7372722081000	2.5853908292000	10.2108166465000
H52	4.4834217235000	2.5547765696000	9.1366331868000
H53	3.8764807780000	3.0150550319000	10.7493284747000
C54	5.8104019630000	4.6741781623000	9.9837943827000
H55	4.9926190881000	5.1963730425000	10.5016716766000
H56	5.5865467778000	4.6840328035000	8.9027181543000
C57	7.1437299914000	5.3817957422000	10.2045501108000
H58	7.9471420901000	4.7274245662000	9.8507433696000
H59	7.1511855158000	6.2973380764000	9.5887549033000
C60	6.6452176777000	6.7228057844000	12.1071881404000
H61	5.6712787263000	6.3331053687000	12.4280031366000
H62	6.4742206757000	7.5260768363000	11.3718919588000
C63	7.3593459984000	7.3098443713000	13.3070124428000
H64	6.6214350830000	7.8446208338000	13.9235514282000
H65	7.7733475002000	6.4934013937000	13.9145754938000
C66	9.4073516355000	8.4986310286000	13.7715930165000
H67	10.2310371809000	8.9211328174000	13.1847954345000
H68	9.7793446220000	7.5909248800000	14.2741954462000
C69	9.0111443066000	9.5247794896000	14.8279414499000
H70	8.4885379599000	10.3772106260000	14.3874530191000
H71	9.8843098560000	9.8817076712000	15.3841543521000
C72	7.0806817242000	9.5134043479000	16.2729822503000
C73	6.0920439317000	8.5464781709000	16.8580232464000
C74	4.9262027996000	9.0168952142000	17.4601221298000
H75	4.7826804235000	10.0867984641000	17.5745542901000
C76	3.9674055991000	8.0915876859000	17.8725849215000
H77	3.0435094312000	8.4091196680000	18.3436339528000
C78	4.1930132190000	6.7405988863000	17.6436449319000
H79	3.4527100342000	5.9980141593000	17.9144746975000
C80	6.2674561128000	7.1727500237000	16.6964682638000
H81	7.1547714089000	6.7628111918000	16.2370057765000
H80	7.8618053324000	4.0198864908000	15.5916952690000

Catalyst 3 at Ru^{V} oxidation state

E(B3LYP-D3/LACV3P**++) (a.u.) = -2453.628909

Solvation energy (kcal mol^{-1}) = -67.189

Zero-point energy (kcal mol^{-1}) = 380.152

ΔH_{298} (kcal mol^{-1}) = 27.705

ΔS_{298} (cal K^{-1} mol^{-1}) = 256.281

Cartesian coordinates

Ru1	-2.1196617683000	-3.9475535261000	-7.6026320851000
O2	-2.1808034521000	-1.4769871511000	-11.0915924674000
O3	-1.5903075765000	-2.7928131228000	-9.3568052134000
O4	-1.6356852354000	-5.2565586974000	-5.9992321438000
O5	-1.9674978602000	-5.8820802283000	-3.8578219180000
O6	2.4826075425000	0.3727765919000	-5.7177704879000
O7	2.5674703883000	-1.7369105863000	-6.5742915799000
O8	3.2313000674000	-1.9206423618000	-9.4979910329000
O9	1.3902030132000	-3.0267181826000	-11.5934281950000
O10	-0.8963273302000	-3.6477377116000	-13.4966150604000
O11	-3.1727415840000	-5.0332910571000	-12.6844993055000
O12	-3.7774972270000	-7.1797353868000	-13.1368769286000
O13	-0.5024416052000	-4.4460097919000	-7.9722744353000
N16	-3.9364419823000	-2.8132891153000	-8.3657167091000
N17	-3.8754475125000	-4.0831113361000	-6.1793488811000
N18	-1.4006168038000	-2.2730537521000	-6.4887995878000
N19	-2.8852009192000	-5.5799201367000	-8.7322617569000
C20	-2.4286868354000	-2.1100638341000	-10.0751488556000
C21	-3.8234239387000	-2.1525226354000	-9.5225558056000
C22	-4.9053381167000	-1.5354863154000	-10.1445717239000
H23	-4.7248725301000	-1.0158889056000	-11.0789506771000
C24	-6.1532698693000	-1.6147301449000	-9.5353619279000
H25	-7.0253463703000	-1.1573137016000	-9.9919696159000
C26	-6.2698420964000	-2.2827790873000	-8.3152426789000
H27	-7.2280219117000	-2.3466343843000	-7.8127177094000
C28	-5.1385684050000	-2.8693069048000	-7.7469039370000
C29	-5.0900802748000	-3.5545127639000	-6.4562341863000
C30	-6.1386643227000	-3.6485580240000	-5.5407410927000
H31	-7.1070895302000	-3.2217275813000	-5.7747316320000
C32	-5.9214057172000	-4.2839386783000	-4.3163674647000
H33	-6.7260318230000	-4.3567839013000	-3.5914669212000
C34	-4.6666529683000	-4.8207890129000	-4.0441264058000
H35	-4.4206422598000	-5.3342691702000	-3.1211996744000
C36	-3.6749452519000	-4.7078479182000	-5.0151337664000
C37	-2.3138487361000	-5.3321313825000	-4.8859963669000
C38	-3.3994382178000	-6.6785919760000	-8.1411229569000
H39	-3.3267341049000	-6.7227851966000	-7.0626864823000

C40	-3.9641042561000	-7.7078048487000	-8.8854513948000
H41	-4.3595991368000	-8.5796419665000	-8.3759409963000
C42	-4.0083764468000	-7.5969476873000	-10.2747266050000
H43	-4.4439956889000	-8.3691225950000	-10.9014542601000
C44	-3.4663052502000	-6.4597636588000	-10.8749125960000
C45	-2.8868969486000	-5.4817108149000	-10.0721636423000
H46	-2.3947524408000	-4.6251619682000	-10.5103943062000
C47	-3.4940350969000	-6.2913532837000	-12.3645578873000
C48	-2.9297539648000	-4.7132903140000	-14.0762670054000
H49	-3.8895814640000	-4.6178476140000	-14.5943362315000
H50	-2.3553034190000	-5.5236193295000	-14.5321441730000
C51	-2.1558800015000	-3.4123221748000	-14.0840668370000
H52	-2.0605934881000	-3.0758898398000	-15.1314436544000
H53	-2.7122632695000	-2.6373421813000	-13.5312668403000
C54	-0.0388513069000	-2.5128984618000	-13.5619405987000
H55	-0.4830792940000	-1.6700027745000	-13.0141143042000
H56	0.0927530056000	-2.2024542220000	-14.6132723371000
C57	1.3315675453000	-2.8770914695000	-12.9982733455000
H58	1.6368622371000	-3.8395086484000	-13.4214828936000
H59	2.0517492445000	-2.1112547477000	-13.3335632877000
C60	1.2721752206000	-1.8102593373000	-10.8704797526000
H61	0.2248565702000	-1.4923791506000	-10.8087459825000
H62	1.8525581653000	-1.0062387986000	-11.3521393770000
C63	1.8111501887000	-2.0161905392000	-9.4682968150000
H64	1.3801010110000	-1.2460920168000	-8.8106923074000
H65	1.4823731953000	-2.9998474206000	-9.1007079018000
C66	3.9317373721000	-2.4628641997000	-8.3993842647000
H67	4.9767253796000	-2.5522601245000	-8.7164479919000
H68	3.5659852297000	-3.4714406874000	-8.1470352471000
C69	3.8948858589000	-1.5933617841000	-7.1470402216000
H70	4.0740923836000	-0.5405378696000	-7.3780740686000
H71	4.6232014218000	-1.9322455103000	-6.4032492177000
C72	1.9654826223000	-0.6788350563000	-6.0214636585000
C73	0.4927564086000	-0.9384836502000	-5.8878881001000
C74	-0.3404197105000	0.0147477849000	-5.3042171663000
H75	0.0974667046000	0.9111091534000	-4.8760973147000
C76	-1.7166566614000	-0.2084382107000	-5.3124492297000
H77	-2.4066541565000	0.5025057715000	-4.8711426573000
C78	-2.2109641058000	-1.3522856552000	-5.9256704810000
H79	-3.2755998250000	-1.5394038676000	-5.9818258101000
C80	-0.0714108728000	-2.0786289161000	-6.4537511674000
H81	0.5483617117000	-2.8349355903000	-6.9116068121000

Catalyst 4 at Ru^{II} oxidation state

E(B3LYP-D3/LACV3P**++) (a.u.) = -2270.984494

Solvation energy (kcal mol^{-1}) = -29.383

Zero-point energy (kcal mol^{-1}) = 422.836

ΔH_{298} (kcal mol^{-1}) = 27.403

ΔS_{298} (cal K^{-1} mol^{-1}) = 254.361

Cartesian coordinates

Ru1	-2.8869097313000	-3.2301793824000	-10.1308682623000
O3	-1.2798488528000	-3.0825913757000	-8.6034184571000
C4	-0.0943982657000	-3.0563942608000	-9.1194739851000
O5	0.9876955239000	-2.9995124745000	-8.5277178119000
C6	-0.3110348266000	-3.3129788782000	-13.4168192791000
C7	-1.4358231799000	-3.3217771687000	-12.5898906359000
N8	-1.2532321619000	-3.2421957162000	-11.2363121303000
C9	-0.0473980943000	-3.1376793798000	-10.6571720508000
C10	1.0987087718000	-3.1275382558000	-11.4450558301000
C11	0.9587887273000	-3.2188623939000	-12.8350157691000
C12	-5.6335884632000	-3.2617952853000	-13.2876286039000
C13	-5.0484490490000	-3.2461738406000	-12.0257199120000
N14	-3.7142118515000	-3.3049826832000	-11.9120168406000
C15	-2.8632813688000	-3.3567774356000	-12.9815332813000
C16	-3.4116509132000	-3.3786611572000	-14.2644749232000
C17	-4.8030050711000	-3.3351005505000	-14.4115510957000
O18	-5.0319685766000	-3.1908973841000	-9.6286015443000
C19	-5.7948872773000	-3.1620096481000	-10.6796642671000
O20	-7.0203175147000	-3.0993553077000	-10.7233681340000
C21	-2.8320284343000	-8.0838307073000	-9.3237960119000
C22	-4.0213389702000	-7.4443822604000	-9.6632657222000
C23	-4.0120455554000	-6.0775767813000	-9.9262951963000
N24	-2.8792654937000	-5.3422450446000	-9.8852225880000
C25	-1.7284306346000	-5.9653077958000	-9.5732870579000
C26	-1.6611670646000	-7.3246374747000	-9.2781988571000
C27	-2.9707836370000	1.6375410603000	-9.7831746678000
C28	-2.7339807939000	1.0594566790000	-11.0263250856000
C29	-2.7248663966000	-0.3271145605000	-11.1323431114000
N30	-2.9429629178000	-1.1352614067000	-10.0756203855000
C31	-3.1858498495000	-0.5804706854000	-8.8724139467000
C32	-3.2034881701000	0.8018005732000	-8.6881653071000
O33	-3.3025394238000	2.6040909917000	-7.1161153171000
O34	-3.7113098832000	0.4801236062000	-6.4280925998000
C35	-2.2149823309000	-0.9884078360000	-4.1502784312000
C36	-0.4164019644000	-3.6416619883000	-4.6918178382000
C37	1.3414164881000	-5.7369526752000	-6.4266370029000
O38	0.6370599514000	-7.0711412306000	-8.9627015123000

O39	-0.2853308466000	-9.0800239328000	-8.4107494859000
C40	-0.3734861889000	-7.9467968763000	-8.8392233742000
C41	1.9101510058000	-7.3475210202000	-8.3233496470000
C42	2.3165347039000	-6.0960468448000	-7.5561755655000
C43	1.8539214994000	-4.5794908340000	-5.5603370207000
C44	0.9523677972000	-4.2596394582000	-4.3546742291000
C45	-0.3206260877000	-2.2554149770000	-5.3468760831000
C46	-1.6720056902000	-1.5345877641000	-5.4826459131000
C47	-3.6288752025000	-0.3807795043000	-4.1916731239000
C48	-3.7731232542000	0.8784580875000	-5.0358819560000
C49	-3.4145198572000	1.4142306301000	-7.3394269425000
H50	-0.4228157033000	-3.3767259262000	-14.4941337754000
H51	2.0620109659000	-3.0493589529000	-10.9534612959000
H52	1.8386136868000	-3.2142481168000	-13.4710450983000
H53	-6.7147875383000	-3.2131763834000	-13.3538616240000
H54	-2.7659272279000	-3.4219154668000	-15.1351137831000
H55	-5.2345728409000	-3.3511779375000	-15.4076515393000
H56	-2.7893727277000	-9.1410153211000	-9.0824428432000
H57	-4.9598478054000	-7.9864725598000	-9.7070535088000
H58	-4.9224766010000	-5.5341019376000	-10.1410180320000
H59	-0.8368745994000	-5.3628596616000	-9.5272689926000
H60	-2.9743596111000	2.7124619551000	-9.6348438067000
H61	-2.5501762681000	1.6670685431000	-11.9060041947000
H62	-2.5347211009000	-0.8161956730000	-12.0808112515000
H63	-3.3489440706000	-1.2638779358000	-8.0501926561000
H64	2.6213014827000	-7.5952584100000	-9.1187134902000
H65	1.7942135877000	-8.2187886283000	-7.6724452957000
H66	3.3212941536000	-6.2734881650000	-7.1474818024000
H67	2.3902018252000	-5.2474149210000	-8.2469828958000
H68	1.9708801182000	-3.6950256640000	-6.1963613598000
H69	2.8557912313000	-4.8367902602000	-5.1866696231000
H70	0.8001465970000	-5.1811508175000	-3.7737865014000
H71	1.4869595122000	-3.5676091584000	-3.6885082846000
H72	0.1151284428000	-2.3643984355000	-6.3436452039000
H73	0.3671922114000	-1.6237878917000	-4.7625211351000
H74	-1.5591918969000	-0.7103790544000	-6.1943536430000
H75	-2.4029102008000	-2.2199048569000	-5.9335575682000
H76	-3.9370234402000	-0.1174130577000	-3.1711574192000
H77	-4.3510725072000	-1.1240131553000	-4.5535180182000
H78	-2.9771739703000	1.6052615884000	-4.8421436610000
H79	-4.7351369101000	1.3759277508000	-4.8703308780000
H80	-1.5146725218000	-0.2329074653000	-3.7637675459000
H81	-2.2247294850000	-1.7945299676000	-3.4060889822000
H82	-0.9819030617000	-4.3059302971000	-5.3594843353000

H83	-0.9983315245000	-3.5817825535000	-3.7618500844000
H84	1.1689392552000	-6.6223738276000	-5.7953259108000
H85	0.3756039035000	-5.4665910692000	-6.8680331607000

Catalyst 4 at Ru^{III} oxidation state

E(B3LYP-D3/LACV3P**++) (a.u.) = -2347.238787

Solvation energy (kcal mol^{-1}) = -57.651

Zero-point energy (kcal mol^{-1}) = 439.528

ΔH_{298} (kcal mol^{-1}) = 29.150

ΔS_{298} (cal K^{-1} mol^{-1}) = 264.835

Cartesian coordinates

Ru1	-2.9322692219000	-3.2834153535000	-10.0101138364000
O2	-3.5373446273000	-2.9483042629000	-7.5631936093000
O3	-5.0474113200000	-3.4325150608000	-9.6187420582000
C4	-5.8582555939000	-3.3530366052000	-10.6368945802000
O5	-7.0798171123000	-3.3900971660000	-10.6156302640000
C6	-3.5716822472000	-2.7592916804000	-14.1946505733000
C7	-2.9989499232000	-2.9191763751000	-12.9331554759000
N8	-3.8020091378000	-3.1082875847000	-11.8545799994000
C9	-5.1364327538000	-3.1610979387000	-11.9604362102000
C10	-5.7595351973000	-3.0124641986000	-13.1947122829000
C11	-4.9632431935000	-2.8044486070000	-14.3217716038000
C12	1.0100506072000	-3.1878345459000	-11.5891765783000
C13	-0.1069442023000	-3.2791920961000	-10.7649511138000
N14	-1.3410159095000	-3.1530524211000	-11.2699562880000
C15	-1.5671459896000	-2.9475439662000	-12.5941661959000
C16	-0.4817699687000	-2.8381621153000	-13.4628730067000
C17	0.8149512326000	-2.9556110190000	-12.9515896452000
O18	-1.2652129499000	-3.4940357707000	-8.7197339518000
C19	-0.0730458190000	-3.5210551777000	-9.2639544419000
O20	0.9899535761000	-3.6906692582000	-8.6973801006000
C21	-3.1650384468000	1.5641256226000	-9.2577846570000
C22	-1.9930033351000	0.8696566932000	-8.9651785744000
C23	-1.9279142803000	-0.4960186746000	-9.2219067127000
N24	-2.9592658817000	-1.1699013088000	-9.7728332819000
C25	-4.0854444622000	-0.4990908456000	-10.0627022426000
C26	-4.2333103430000	0.8614005488000	-9.8176712869000
C27	-3.1033046542000	-8.1796465616000	-10.1286978772000
C28	-2.6138571294000	-7.4929838125000	-11.2367948113000
C29	-2.5607571043000	-6.1052605911000	-11.1969176108000
N30	-2.9587406988000	-5.3991283337000	-10.1186018997000
C31	-3.4220361795000	-6.0598279840000	-9.0421928559000
C32	-3.5131799813000	-7.4499644952000	-9.0109272268000
O33	-4.3052415514000	-9.3756417437000	-7.8423154104000
O34	-4.3470097477000	-7.3458371147000	-6.8232264500000
C35	-6.7436887447000	-5.9256850611000	-5.4865890529000
C36	-7.5309491069000	-2.8884895628000	-6.5789752146000
C37	-8.2028550854000	-0.5815925654000	-8.8040994619000

O38	-6.3891151089000	0.7119826258000	-10.7056371196000
O39	-5.7913631835000	2.6638201327000	-9.6895307037000
C40	-5.5481337180000	1.5376774931000	-10.0629933579000
C41	-7.8100043621000	1.0358533704000	-10.7416446414000
C42	-8.5706845350000	-0.1846316797000	-10.2415181475000
C43	-9.1258681059000	-1.6727487502000	-8.2461998128000
C44	-8.8180917887000	-2.0713835871000	-6.7912421858000
C45	-7.5484659537000	-4.2579962569000	-7.2766998967000
C46	-6.4309309187000	-5.2064169324000	-6.8097529464000
C47	-5.5782549519000	-6.7091152026000	-4.8568530654000
C48	-5.0623621412000	-7.8851153531000	-5.6732259219000
C49	-4.0887379403000	-8.1825420269000	-7.8347037490000
H50	-2.9437328337000	-2.6018429156000	-15.0640867199000
H51	-6.8422980345000	-3.0582020919000	-13.2319112019000
H52	-5.4183488914000	-2.6776713443000	-15.2988568731000
H53	1.9904925552000	-3.3012141960000	-11.1393011156000
H54	-0.6452026239000	-2.6697955434000	-14.5214875780000
H55	1.6657845523000	-2.8719952164000	-13.6202298036000
H56	-3.2774928196000	2.6234162041000	-9.0491142176000
H57	-1.1388485847000	1.3686079350000	-8.5205205146000
H58	-1.0565590231000	-1.0807014465000	-8.9580608189000
H59	-4.9113659480000	-1.0557650207000	-10.4720285974000
H60	-3.1835081021000	-9.2618862672000	-10.1083937424000
H61	-2.2832358403000	-8.0187967822000	-12.1259031920000
H62	-2.1962845851000	-5.5365997938000	-12.0438463580000
H63	-3.7385415243000	-5.4581678079000	-8.2028622298000
H64	-8.0517240131000	1.2823805525000	-11.7799411123000
H65	-7.9804282560000	1.9187210635000	-10.1203697857000
H66	-9.6414844397000	0.0508333279000	-10.3059396498000
H67	-8.3893868048000	-1.0352131190000	-10.9102309296000
H68	-9.0755549526000	-2.5505481167000	-8.9014391162000
H69	-10.1613151356000	-1.3094777742000	-8.2945144412000
H70	-8.7750136140000	-1.1625044204000	-6.1754679340000
H71	-9.6598831342000	-2.6599359876000	-6.4025903855000
H72	-7.4674661692000	-4.1102554905000	-8.3562417048000
H73	-8.5186024158000	-4.7446022264000	-7.0999728816000
H74	-6.2284780589000	-5.9456393484000	-7.5909163809000
H75	-5.4905025019000	-4.6452665429000	-6.6835906707000
H76	-5.9001766960000	-7.1120971998000	-3.8886824385000
H77	-4.7367745068000	-6.0359345620000	-4.6450367223000
H78	-5.8695184313000	-8.5277396179000	-6.0391640642000
H79	-4.3624454416000	-8.5063106358000	-5.1056647983000
H80	-7.5894664739000	-6.6077209032000	-5.6505446761000
H81	-7.0898756486000	-5.1890218216000	-4.7510957090000

H82	-6.6584984416000	-2.3184436583000	-6.9342323953000
H83	-7.3836615621000	-3.0206305698000	-5.4984728196000
H84	-8.2507360803000	0.3050936139000	-8.1547658542000
H85	-7.1619571394000	-0.9277710260000	-8.7858757399000
H86	-4.4418059321000	-3.2572857746000	-7.4068605059000
H87	-2.9360378856000	-3.4298761908000	-6.9792642765000

Catalyst 4 at Ru^{IV} oxidation state

E(B3LYP-D3/LACV3P**++) (a.u.) = -2346.587043

Solvation energy (kcal mol^{-1}) = -60.386

Zero-point energy (kcal mol^{-1}) = 433.022

ΔH_{298} (kcal mol^{-1}) = 28.445

ΔS_{298} (cal K^{-1} mol^{-1}) = 260.442

Cartesian coordinates

Ru1	-2.9508547169000	-3.2568853213000	-9.8656228066000
O2	-3.3947176973000	-3.2464424174000	-8.0015964760000
O3	-1.1315587228000	-3.0215712829000	-8.8100136590000
C4	0.0445147765000	-3.1663368455000	-9.3534799377000
O5	1.1184858999000	-3.1406014229000	-8.7758489515000
C6	-0.4675883771000	-3.6039196113000	-13.5303986761000
C7	-1.5260721613000	-3.4403272616000	-12.6350140813000
N8	-1.3036004807000	-3.3571792104000	-11.3012180855000
C9	-0.0491119272000	-3.3765539607000	-10.8360989311000
C10	1.0527674231000	-3.5352942516000	-11.6695286773000
C11	0.8373016096000	-3.6670354753000	-13.0389636383000
C12	-5.6453905620000	-2.8837343183000	-13.3011917956000
C13	-5.0520482471000	-3.0001036759000	-12.0491932156000
N14	-3.7342935887000	-3.1740435875000	-11.8934129537000
C15	-2.9349867164000	-3.2882998117000	-12.9810613856000
C16	-3.4682837284000	-3.2095312127000	-14.2682519254000
C17	-4.8369779129000	-2.9912622248000	-14.4303011029000
O18	-5.0424459570000	-3.4363566338000	-9.7712242708000
C19	-5.8179622562000	-3.0625553806000	-10.7598229059000
O20	-7.0151515771000	-2.8666999488000	-10.7104424740000
C21	-2.5732956637000	-8.1606249642000	-9.9424790697000
C22	-3.7291632290000	-7.5436567711000	-10.4189035872000
C23	-3.8375281404000	-6.1590567188000	-10.3517124462000
N24	-2.8583274774000	-5.3914302173000	-9.8250018939000
C25	-1.7549605171000	-5.9859286020000	-9.3362920184000
C26	-1.5649657965000	-7.3638686941000	-9.3983883534000
C27	-2.9651038552000	1.6504362235000	-9.6416576144000
C28	-2.6821226203000	1.0177627220000	-10.8510784624000
C29	-2.7142258560000	-0.3684110439000	-10.9061657734000
N30	-3.0315486350000	-1.1267618370000	-9.8343913777000
C31	-3.3198362798000	-0.5227790035000	-8.6683930115000
C32	-3.2841488942000	0.8655225557000	-8.5348413185000
O33	-3.3641415999000	2.7147967689000	-7.0237925203000
O34	-3.7996851815000	0.6112150233000	-6.2745288302000
C35	-2.0926377667000	-0.7828089166000	-4.1219346337000
C36	-0.2671197909000	-3.4060716539000	-4.6014216169000
C37	1.4469693653000	-5.6212524940000	-6.2011446997000

O38	0.6047273565000	-7.0319018012000	-8.6275605715000
O39	-0.1451347073000	-9.1791037035000	-8.7530789207000
C40	-0.2960322749000	-7.9844260426000	-8.8884053840000
C41	1.8579062077000	-7.3995671001000	-7.9790631698000
C42	2.3774102472000	-6.1383172415000	-7.3070459645000
C43	1.9843275278000	-4.3586870057000	-5.5161186584000
C44	1.1529184615000	-3.9177918993000	-4.2988353902000
C45	-0.3000891393000	-2.1037071528000	-5.4181417575000
C46	-1.6801432415000	-1.4223443825000	-5.4609954213000
C47	-3.5281657398000	-0.2291202750000	-4.0420230610000
C48	-3.8040522998000	1.0136877184000	-4.8765516125000
C49	-3.4992434093000	1.5224532361000	-7.2021916508000
H50	-0.6601868831000	-3.6765712872000	-14.5940739058000
H51	2.0369001508000	-3.5409593538000	-11.2137297614000
H52	1.6706197396000	-3.8001417393000	-13.7214104777000
H53	-6.7185976830000	-2.7328690186000	-13.3438423860000
H54	-2.8203059162000	-3.3070267368000	-15.1312645448000
H55	-5.2615648617000	-2.9163996153000	-15.4262571201000
H56	-2.4350410868000	-9.2367963154000	-9.9793476063000
H57	-4.5430358004000	-8.1221926091000	-10.8421753658000
H58	-4.7184903592000	-5.6360736974000	-10.6998841736000
H59	-1.0159436388000	-5.3497333395000	-8.8735634334000
H60	-2.9298633703000	2.7296916874000	-9.5310826265000
H61	-2.4236035001000	1.5847563314000	-11.7387919320000
H62	-2.4736243704000	-0.8923011677000	-11.8223529105000
H63	-3.5578517350000	-1.1710777776000	-7.8367456299000
H64	2.5344983611000	-7.7811489825000	-8.7508561530000
H65	1.6547701151000	-8.2060023260000	-7.2679859160000
H66	3.3679692000000	-6.3655912159000	-6.8922738445000
H67	2.5174670916000	-5.3570431210000	-8.0644760891000
H68	2.0418240694000	-3.5513683161000	-6.2560538429000
H69	3.0136233463000	-4.5495714389000	-5.1824341806000
H70	1.0879182650000	-4.7601745158000	-3.5960126112000
H71	1.6972006151000	-3.1243698300000	-3.7691669988000
H72	0.0194815432000	-2.3135093736000	-6.4444069428000
H73	0.4339590060000	-1.3973006780000	-5.0019761411000
H74	-1.6628836009000	-0.6531070450000	-6.2403227020000
H75	-2.4374830347000	-2.1522186475000	-5.7794975134000
H76	-3.7506398415000	0.0384589339000	-3.0012483658000
H77	-4.2506352653000	-1.0065088159000	-4.3230831726000
H78	-3.0468796895000	1.7913328411000	-4.7364046969000
H79	-4.7861179722000	1.4457232312000	-4.6586643264000
H80	-1.3841061210000	0.0212023241000	-3.8755157418000
H81	-1.9881919509000	-1.5262710052000	-3.3229859800000

H82	-0.8488651709000	-4.1744553587000	-5.1306196286000
H83	-0.7776761803000	-3.2571644648000	-3.6416576575000
H84	1.3003528874000	-6.4140642545000	-5.4526124221000
H85	0.4602411615000	-5.4145236934000	-6.6328400162000
H86	-3.8033347275000	-4.0966220914000	-7.7705732314000

Catalyst 4 at Ru^{V} oxidation state

E(B3LYP-D3/LACV3P**++) (a.u.) = -2345.926265

Solvation energy (kcal mol^{-1}) = -60.294

Zero-point energy (kcal mol^{-1}) = 425.949

ΔH_{298} (kcal mol^{-1}) = 28.168

ΔS_{298} (cal K^{-1} mol^{-1}) = 259.175

Cartesian coordinates

Ru1	-3.0060709558000	-3.2859962154000	-9.7040566931000
O2	-3.4732122393000	-3.3233007338000	-8.0355351010000
O3	-1.1487873177000	-3.1099241273000	-8.6140379534000
C4	0.0285228044000	-3.0500391110000	-9.1602504648000
O5	1.0960683844000	-2.9080585537000	-8.5830743040000
C6	-0.3280928703000	-3.5471709720000	-13.3481873352000
C7	-1.4247893159000	-3.4512673348000	-12.4898430215000
N8	-1.2591704641000	-3.3003434448000	-11.1546383333000
C9	-0.0230659279000	-3.2155748517000	-10.6521644198000
C10	1.1176648624000	-3.2883885372000	-11.4469623207000
C11	0.9608795992000	-3.4680951162000	-12.8177213988000
C12	-5.5330219396000	-3.2480091341000	-13.3854560478000
C13	-4.9990538530000	-3.2395297454000	-12.0994713569000
N14	-3.6875313381000	-3.3302580821000	-11.8615295889000
C15	-2.8273642269000	-3.4433396250000	-12.9004954674000
C16	-3.2910746831000	-3.4812332963000	-14.2158134459000
C17	-4.6614743512000	-3.3795815022000	-14.4617378428000
O18	-5.1357733462000	-3.3328971796000	-9.7754936145000
C19	-5.8437982350000	-3.1708049483000	-10.8597637065000
O20	-7.0504630322000	-3.0289513698000	-10.9263158422000
C21	-2.6706736035000	-8.1860579920000	-9.8104356696000
C22	-3.8790640196000	-7.5679014654000	-10.1306187843000
C23	-3.9692541834000	-6.1807854154000	-10.0838768064000
N24	-2.9090032170000	-5.4175888417000	-9.7450121816000
C25	-1.7476897892000	-6.0071587520000	-9.4182601112000
C26	-1.5843299617000	-7.3878829460000	-9.4483100796000
C27	-3.0065800109000	1.6305806878000	-9.6302020852000
C28	-2.7833183528000	0.9616336365000	-10.8326483618000
C29	-2.8089327941000	-0.4261497332000	-10.8464218616000
N30	-3.0588936500000	-1.1516791738000	-9.7343014405000
C31	-3.2921674001000	-0.5109533107000	-8.5746719686000
C32	-3.2659721859000	0.8789429171000	-8.4851734033000
O33	-3.2638699591000	2.7587126590000	-7.0127942762000
O34	-3.7323854732000	0.6818540541000	-6.2105634654000
C35	-2.0239424606000	-0.7316647704000	-4.0653524522000
C36	-0.4013506564000	-3.4898276403000	-4.7621899096000
C37	1.3607159489000	-5.6885079663000	-6.4097682168000

O38	0.6524345613000	-7.0456449638000	-8.9087481965000
O39	-0.1272405887000	-9.1877671411000	-8.8490284805000
C40	-0.2758086749000	-8.0009950597000	-9.0399633937000
C41	1.9198123589000	-7.3586961294000	-8.2590371310000
C42	2.3482515306000	-6.1050017959000	-7.5097880738000
C43	1.8628129215000	-4.4989704473000	-5.5805081860000
C44	0.9448920996000	-4.1410683179000	-4.3973826764000
C45	-0.2707286833000	-2.0836309171000	-5.3702425746000
C46	-1.6040883797000	-1.3150390678000	-5.4279163214000
C47	-3.4424031738000	-0.1388974973000	-3.9766173068000
C48	-3.6893621824000	1.1054185244000	-4.8177301588000
C49	-3.4314303039000	1.5675676850000	-7.1607936780000
H50	-0.4779304764000	-3.6762124352000	-14.4136170158000
H51	2.0823397751000	-3.2040563194000	-10.9588899653000
H52	1.8248943001000	-3.5400222306000	-13.4708379102000
H53	-6.6087163071000	-3.1585204554000	-13.4901720572000
H54	-2.5922407969000	-3.5754918647000	-15.0384432575000
H55	-5.0344299528000	-3.3997615280000	-15.4809140299000
H56	-2.5541024483000	-9.2654165166000	-9.8214274800000
H57	-4.7534513278000	-8.1482934619000	-10.4038166871000
H58	-4.8930449689000	-5.6554646516000	-10.2880844930000
H59	-0.9420217273000	-5.3682530830000	-9.0964467881000
H60	-2.9738397599000	2.7130594517000	-9.5551879062000
H61	-2.5783514143000	1.5023379565000	-11.7502065746000
H62	-2.6211703025000	-0.9774327617000	-11.7579964394000
H63	-3.4830071326000	-1.1202378316000	-7.7030616439000
H64	2.6299482884000	-7.6474196151000	-9.0406353997000
H65	1.7652609667000	-8.2143848226000	-7.5955474197000
H66	3.3358540010000	-6.3051453934000	-7.0739167010000
H67	2.4760931168000	-5.2785536779000	-8.2203683985000
H68	1.9920549858000	-3.6335584096000	-6.2408930134000
H69	2.8587254436000	-4.7440254826000	-5.1866304268000
H70	0.7574904781000	-5.0517260816000	-3.8113985291000
H71	1.4840051850000	-3.4581921855000	-3.7271437733000
H72	0.1414255100000	-2.1675667526000	-6.3797963256000
H73	0.4537888754000	-1.4975019264000	-4.7853451696000
H74	-1.5173178068000	-0.5030224524000	-6.1589321220000
H75	-2.3899084891000	-1.9853345677000	-5.8057547295000
H76	-3.6493396562000	0.1377825580000	-2.9351695257000
H77	-4.1888439871000	-0.8960061722000	-4.2500944483000
H78	-2.8998710770000	1.8553181396000	-4.7078209963000
H79	-4.6494753106000	1.5772182002000	-4.5862383826000
H80	-1.2957274520000	0.0376807212000	-3.7715527167000
H81	-1.9503994559000	-1.5178410887000	-3.3043272622000

H82	-0.9613621299000	-4.1244272122000	-5.4635328100000
H83	-1.0109508898000	-3.4434412016000	-3.8501852885000
H84	1.1771421769000	-6.5462847177000	-5.7458468441000
H85	0.3963456234000	-5.4382581740000	-6.8684840479000

Back conformation of catalyst 1 at Ru^V oxidation state

E(B3LYP-D3/LACV3P**++) (a.u.) = -2299.740366

Solvation energy (kcal mol^{-1}) = -64.899

Zero-point energy (kcal mol^{-1}) = 341.480

ΔH_{298} (kcal mol^{-1}) = 25.327

ΔS_{298} (cal K^{-1} mol^{-1}) = 233.511

Cartesian coordinates

Ru1	-1.2870940444000	-0.0877541990000	-0.6462909052000
O2	2.1843536992000	5.4351553305000	-0.9011419653000
O3	2.5758371480000	3.4057258318000	0.0373540557000
O4	5.1182364419000	1.7291182561000	0.1513754176000
O5	5.3080500625000	-1.1820916062000	-0.0427987070000
O6	2.9979541759000	-3.1222854650000	-0.4565310150000
O7	2.6409208072000	-5.1852685632000	0.4171158506000
O8	-1.6062211610000	-0.0620132030000	-2.7636693057000
O9	-0.8550572659000	0.3908384194000	-4.8385572739000
O10	-2.0467211266000	-0.1753082406000	1.3543379587000
O11	-1.7124321834000	-0.5785326903000	3.5453441879000
N12	-1.1921512733000	2.0556241864000	-0.7771507900000
N13	-0.9860532404000	-2.2087358387000	-0.4744204584000
N14	0.4360435863000	-0.1204367472000	0.8134696871000
N15	0.6938273021000	0.1485319525000	-1.7029446452000
C16	0.1534915016000	-0.3259359264000	2.1032170616000
C17	1.1428745063000	-0.4820560631000	3.0688838871000
H18	0.8296135892000	-0.6467776425000	4.0940241216000
C19	2.4713217074000	-0.4266746909000	2.6613061428000
H20	3.2763996108000	-0.5624568331000	3.3769404032000
C21	2.7675137446000	-0.2010633093000	1.3161771451000
H22	3.7838627152000	-0.1932104779000	0.9569920326000
C23	1.7247391339000	-0.0429573394000	0.4065981241000
C24	1.8666622814000	0.2036554642000	-1.0291364494000
C25	3.0572102421000	0.4758567700000	-1.6977789004000
H26	3.9681724359000	0.5696821767000	-1.1298158299000
C27	3.0341178356000	0.6784721113000	-3.0783938421000
H28	3.9555718852000	0.9009818161000	-3.6076903928000
C29	1.8252322025000	0.5976115317000	-3.7609813185000
H30	1.7233222475000	0.7343197392000	-4.8320067250000
C31	0.6739437133000	0.3322231554000	-3.0263281301000
C32	-1.3136919884000	-0.3731474312000	2.4125865107000
C33	-0.6889422240000	0.2223945850000	-3.6434715349000
C34	-1.9644119736000	2.6994210898000	-1.6750400022000
H35	-2.8442862442000	2.1672838835000	-2.0178895642000
C36	-1.6076786464000	3.9534862059000	-2.1690425934000
H37	-2.2582056605000	4.4477748194000	-2.8815912776000

C38	-0.3889786751000	4.5146166792000	-1.7954476936000
H39	-0.0365201885000	5.4512619546000	-2.2156241426000
C40	0.4147832534000	3.8339870179000	-0.8712351083000
C41	-0.0607983963000	2.6445741870000	-0.3405750373000
H42	0.5053837947000	2.1287792780000	0.4160684420000
C43	1.7954511302000	4.3370293642000	-0.5724808556000
C44	3.9417191620000	3.8463131058000	0.2809643515000
H45	3.9085288465000	4.7665851938000	0.8715077558000
H46	4.4171246360000	4.0742934610000	-0.6773725259000
C47	4.7314905639000	2.7857059717000	1.0260860894000
H48	5.6254493324000	3.2823028116000	1.4323126359000
H49	4.1497429106000	2.3933769924000	1.8740956076000
C50	6.2068577931000	0.9382953206000	0.6271164374000
H51	6.0247182335000	0.5917324181000	1.6559981393000
H52	7.1347668076000	1.5296617026000	0.6281155257000
C53	6.3762806261000	-0.2674047310000	-0.2837805198000
H54	6.3819703171000	0.0677587642000	-1.3322935195000
H55	7.3435493631000	-0.7494164299000	-0.0769338043000
C56	5.2386334484000	-2.2659864182000	-0.9648956426000
H57	6.2479411722000	-2.6599062342000	-1.1574612930000
H58	4.8182493416000	-1.9320365458000	-1.9257567419000
C59	4.4237161024000	-3.4113225208000	-0.3931331156000
H60	4.7020569848000	-3.5959345598000	0.6484104391000
H61	4.6172965287000	-4.3245785432000	-0.9634032682000
C62	2.2119080981000	-4.1368330563000	-0.0084916824000
C63	0.7521706631000	-3.7963700575000	-0.0182956179000
C64	-0.1483253903000	-4.5740668382000	0.7221186388000
H65	0.2096596190000	-5.4699116185000	1.2195493942000
C66	-1.4737810782000	-4.1580755030000	0.8198305052000
H67	-2.2018563441000	-4.7310896681000	1.3827234524000
C68	-1.8547899773000	-2.9455973089000	0.2462597327000
H69	-2.8411945626000	-2.5191503872000	0.3877210121000
C70	0.2705674054000	-2.6626086607000	-0.6538592795000
H71	0.9240422395000	-2.0788916690000	-1.2796999273000
O72	-3.0085235242000	-0.1837709807000	-0.8333009654000

Back conformation of catalyst 2 at Ru^{V} oxidation state

E(B3LYP-D3/LACV3P**++) (a.u.) = -2227.916148

Solvation energy (kcal mol^{-1}) = -62.414

Zero-point energy (kcal mol^{-1}) = 372.291

ΔH_{298} (kcal mol^{-1}) = 25.503

ΔS_{298} (cal K^{-1} mol^{-1}) = 233.750

Cartesian coordinates

Ru1	-11.2951564330000	-0.5222822923000	-1.1506601079000
O2	-13.2428955824000	5.2735171379000	1.3768843816000
O3	-14.1573031799000	3.2016282845000	1.5231030686000
C4	-15.5059843433000	1.6666585132000	3.9036558658000
C5	-15.6171897221000	-1.9789129040000	4.0927233637000
O6	-13.6424767816000	-3.7872777677000	2.4651105362000
O7	-13.8393083871000	-5.9147278157000	1.7043126871000
O8	-9.3433663527000	-0.2820132468000	-0.2904839502000
O9	-8.0354039542000	0.1264087078000	1.4988126306000
O10	-12.5214002275000	-0.8257149182000	-2.8912952922000
O11	-14.5478194795000	-1.1916268149000	-3.8112179034000
N12	-11.3782830984000	1.6332114139000	-1.1013757489000
N13	-11.4818139656000	-2.6568728711000	-0.8794360896000
N14	-13.4481844131000	-0.5987124830000	-0.5358878846000
N15	-11.4911085059000	-0.2487988854000	1.0649224575000
C16	-14.3737445919000	-0.7341121259000	-1.4906399527000
C17	-15.7394385818000	-0.6687502405000	-1.2278695403000
H18	-16.4228174106000	-0.7954171678000	-2.0604810267000
C19	-16.1412572614000	-0.4113889924000	0.0762034426000
H20	-17.1922979060000	-0.3082984120000	0.3276560842000
C21	-15.1711532044000	-0.2769662611000	1.0708887859000
H22	-15.4605358405000	-0.0524566880000	2.0802472113000
C23	-13.8238903018000	-0.4111992305000	0.7514252822000
C24	-12.6951760884000	-0.3078273511000	1.6813177495000
C25	-12.7657233423000	-0.3337552841000	3.0705480158000
H26	-13.7171271962000	-0.4600188615000	3.5533632852000
C27	-11.5998075165000	-0.2221798209000	3.8309824839000
H28	-11.6574745977000	-0.2413976308000	4.9149133447000
C29	-10.3780938524000	-0.0977632074000	3.1816822103000
H30	-9.4255884076000	-0.0005080096000	3.6908348049000
C31	-10.3731777778000	-0.1379051685000	1.7892415548000
C32	-13.8167618787000	-0.9418699307000	-2.8686268630000
C33	-9.1145119403000	-0.0846365311000	0.9746632787000
C34	-10.2692165003000	2.3370495606000	-1.4057163970000
H35	-9.4967740451000	1.8031236817000	-1.9466944192000
C36	-10.1190149082000	3.6626595678000	-1.0017062504000
H37	-9.2180946344000	4.2005355197000	-1.2749069510000

C38	-11.0996045067000	4.2549798661000	-0.2102854357000
H39	-10.9955981331000	5.2658734501000	0.1709117854000
C40	-12.2444891302000	3.5159784742000	0.1172938562000
C41	-12.3599710987000	2.2319175207000	-0.3936769750000
H42	-13.2553737263000	1.6680048874000	-0.1997322843000
C43	-13.2632895832000	4.1065160004000	1.0537091483000
C44	-15.1573064399000	3.7639166571000	2.4402286579000
H45	-15.6893645167000	4.5550434903000	1.9048996397000
H46	-14.6257888212000	4.2292831856000	3.2764206502000
C47	-16.1142765136000	2.6700583357000	2.9087673231000
H48	-16.9731356082000	3.1750293749000	3.3684828239000
H49	-16.5066165402000	2.1470260369000	2.0268912278000
C50	-16.4495606882000	0.5014170017000	4.3026649057000
H51	-17.0062860443000	0.1496648734000	3.4216670721000
H52	-17.2185529572000	0.9053566670000	4.9698734493000
C53	-15.7457309045000	-0.7149043748000	4.9828454737000
H54	-14.7529312283000	-0.4037062423000	5.3395769477000
H55	-16.2883870762000	-0.9969138748000	5.8914951700000
C56	-14.5992288633000	-3.0176679553000	4.5939507460000
H57	-14.9270675597000	-3.4261289557000	5.5581089443000
H58	-13.6263122256000	-2.5446232684000	4.7803824962000
C59	-14.3922746239000	-4.2059932466000	3.6577580442000
H60	-15.3426847954000	-4.6357503691000	3.3258325410000
H61	-13.8217194824000	-5.0016745866000	4.1444667612000
C62	-13.4261430954000	-4.7843731112000	1.5720885378000
C63	-12.6604313256000	-4.3523482494000	0.3515863247000
C64	-12.6281874492000	-5.1903910729000	-0.7715960469000
H65	-13.1074151674000	-6.1628918179000	-0.7196544590000
C66	-11.9963270338000	-4.7425933704000	-1.9286035305000
H67	-11.9512456763000	-5.3608346426000	-2.8181773267000
C68	-11.4610829065000	-3.4559194150000	-1.9653804214000
H69	-11.0407693662000	-3.0270100725000	-2.8675018942000
C70	-12.0286637816000	-3.1212855167000	0.2646266313000
H71	-11.9921667573000	-2.4869356466000	1.1327195992000
O72	-10.1896084835000	-0.6169973833000	-2.4843979565000
H73	-16.6045507233000	-2.4495029867000	4.0019739297000
H74	-15.3343497533000	-1.7143024944000	3.0715563292000
H75	-15.1960272338000	2.1968216039000	4.8133093653000
H76	-14.5787331597000	1.2917023391000	3.4642185029000

Back conformation of catalyst 3 at Ru^V oxidation state

E(B3LYP-D3/LACV3P**++) (a.u.) = -2453.638175

Solvation energy (kcal mol^{-1}) = -65.314

Zero-point energy (kcal mol^{-1}) = 380.150

ΔH_{298} (kcal mol^{-1}) = 27.661

ΔS_{298} (cal K^{-1} mol^{-1}) = 253.815

Cartesian coordinates

Ru1	5.9347754737000	4.3078640355000	16.6358535882000
O2	3.2211566956000	4.7322871259000	13.4026445005000
O3	4.9731805656000	4.8755463198000	14.8121680369000
O4	7.6757215742000	4.2023861306000	17.8816346172000
O5	8.5640040982000	4.1043563778000	19.9520817203000
O6	2.6468620063000	9.0809087856000	19.9143107455000
O7	2.0166019670000	6.9943482248000	19.2887757125000
O8	-0.1825773475000	5.1307609892000	20.6399545094000
O9	0.5320886186000	2.2985034408000	20.6361913863000
O10	1.9371346718000	-0.1181327065000	19.9168334162000
O11	4.0463792657000	-0.5689210648000	17.7876771392000
O12	4.2968601285000	-2.0768168950000	16.1086740655000
O13	7.2234027309000	4.9016370456000	15.6340332179000
N14	3.7499476086000	3.7207545108000	16.7177554090000
N15	5.3536118409000	3.5241550219000	18.6682895273000
N16	5.6115237749000	6.2089510295000	17.5161070634000
N17	6.0988122367000	2.3362996111000	15.8113096448000
C18	3.7569054628000	4.5427971473000	14.4803654682000
C19	3.0260046497000	3.8689163998000	15.6055602139000
C20	1.7067495041000	3.4336506990000	15.5117464540000
H21	1.1857085627000	3.5962891008000	14.5744915366000
C22	1.1330239485000	2.8162381457000	16.6188958021000
H23	0.1080735799000	2.4602486205000	16.5859144493000
C24	1.8862662329000	2.6568405927000	17.7846917940000
H25	1.4627172812000	2.1919390984000	18.6670052776000
C26	3.1989418662000	3.1282130511000	17.8035123075000
C27	4.1149762095000	3.0476472720000	18.9369290865000
C28	3.8055685163000	2.5091170169000	20.1840246743000
H29	2.8209184472000	2.0926818054000	20.3416325303000
C30	4.7824471545000	2.5029518945000	21.1809389054000
H31	4.5574052377000	2.0929279220000	22.1608585083000
C32	6.0458650112000	3.0143632387000	20.9002258975000
H33	6.8607155982000	3.0363052205000	21.6156199738000
C34	6.2931379147000	3.4982201996000	19.6184399557000
C35	7.6420910144000	3.9793008191000	19.1650046447000
C36	6.4109704896000	2.1912272576000	14.5069333969000
H37	6.8053707800000	3.0700207599000	14.0100414284000

C38	6.1957076583000	0.9878234122000	13.8391257426000
H39	6.4669146750000	0.9055261857000	12.7926292533000
C40	5.5894580950000	-0.0688986966000	14.5133145656000
H41	5.3524807155000	-1.0059434955000	14.0194743843000
C42	5.2578996568000	0.0940775598000	15.8633807307000
C43	5.5728962437000	1.2955899617000	16.4820254083000
H44	5.3656621021000	1.4300350943000	17.5309785678000
C45	4.5040311605000	-0.9815072981000	16.5812259401000
C46	3.2795939611000	-1.5640273990000	18.5157346526000
H47	3.8572919632000	-2.4921705001000	18.5514131683000
H48	2.3474552486000	-1.7603003778000	17.9776054185000
C49	2.9913611808000	-1.0738468867000	19.9197815002000
H50	2.7002877293000	-1.9539354132000	20.5138631106000
H51	3.9033799415000	-0.6534223948000	20.3726663028000
C52	1.3345528344000	0.0494949310000	21.1973185059000
H53	2.0634662797000	0.4476821455000	21.9243179788000
H54	0.9794429929000	-0.9177433144000	21.5874250974000
C55	0.1480641092000	0.9920648014000	21.0536281973000
H56	-0.5191414758000	0.6027086129000	20.2777532469000
H57	-0.4100748193000	1.0274215179000	21.9994596067000
C58	0.8119351341000	3.2345022466000	21.6745414487000
H59	1.6956126099000	2.9306130582000	22.2588706720000
H60	-0.0410930166000	3.3154769852000	22.3623733349000
C61	1.0663670696000	4.5844924124000	21.0242240650000
H62	1.5856570562000	5.2453381463000	21.7354921993000
H63	1.7277496662000	4.4521082367000	20.1566507103000
C64	-0.1732309172000	6.0098942265000	19.5326883211000
H65	-1.2192885348000	6.2962659836000	19.3886134218000
H66	0.1670723937000	5.4947697216000	18.6184850504000
C67	0.6533441886000	7.2782230474000	19.7141410853000
H68	0.6548465989000	7.6102882368000	20.7561524357000
H69	0.2690337753000	8.0941417694000	19.0939612717000
C70	2.8914707529000	8.0160862746000	19.3938463376000
C71	4.2179219163000	7.6830754673000	18.7837749900000
C72	5.2929365501000	8.5629200969000	18.9510094946000
H73	5.1429371666000	9.4758306468000	19.5185900580000
C74	6.5235862482000	8.2376307188000	18.3884474264000
H75	7.3837805438000	8.8881376369000	18.4992336359000
C76	6.6573081599000	7.0448989796000	17.6841838031000
H77	7.5997698481000	6.7219822577000	17.2580228928000
C78	4.4144562341000	6.5234651013000	18.0431751662000
H79	3.6003540350000	5.8337902008000	17.8777466460000

Back conformation of catalyst 4 at Ru^{V} oxidation state

E(B3LYP-D3/LACV3P**++) (a.u.) = -2345.921354

Solvation energy (kcal mol^{-1}) = -60.413

Zero-point energy (kcal mol^{-1}) = 426.063

ΔH_{298} (kcal mol^{-1}) = 27.964

ΔS_{298} (cal K^{-1} mol^{-1}) = 253.110

Cartesian coordinates

Ru1	5.9508928411000	4.3175335839000	16.5950297088000
O2	3.3867506168000	4.8521707190000	13.2502437091000
O3	5.0955702895000	4.9103095170000	14.7204788851000
O4	7.6430235182000	4.1541465415000	17.9010893959000
O5	8.4528974290000	4.0030614657000	20.0006607776000
O6	2.8404248537000	9.0402530220000	20.1318491148000
O7	2.0482632879000	7.0681678678000	19.3461147534000
C8	-0.4352291552000	5.2246435848000	20.5580591124000
C9	-0.0940189945000	2.0758499389000	21.0012941236000
C10	1.7958433954000	-0.4376888031000	20.0061580334000
O11	4.1299392614000	-0.6602065099000	17.7991221290000
O12	4.5023660659000	-2.1558048964000	16.1330018066000
O13	7.2962949309000	4.8751489037000	15.6490204268000
N14	3.7530742132000	3.8161967394000	16.5801666000000
N15	5.2733894387000	3.5518759001000	18.5943515680000
N16	5.6421331272000	6.2209563913000	17.4931435915000
N17	6.0908781491000	2.3355855147000	15.7968086005000
C18	3.8780719097000	4.6403328491000	14.3448430747000
C19	3.0756942928000	4.0043268872000	15.4434042649000
C20	1.7401459352000	3.6397728201000	15.2985108674000
H21	1.2637882124000	3.8319108861000	14.3433590624000
C22	1.0957843926000	3.0510315196000	16.3808878034000
H23	0.0559856901000	2.7473662779000	16.3122476252000
C24	1.8035860841000	2.8541710746000	17.5679057211000
H25	1.3225544846000	2.4023131406000	18.4234232528000
C26	3.1376796217000	3.2511541315000	17.6451908344000
C27	4.0048464410000	3.1313850033000	18.8164182464000
C28	3.6318257604000	2.6241264811000	20.0573735178000
H29	2.6269884335000	2.2676908760000	20.2140651720000
C30	4.5661191048000	2.5813951048000	21.0932188410000
H31	4.2787478576000	2.1942550927000	22.0657479137000
C32	5.8599405719000	3.0309169415000	20.8556442083000
H33	6.6479796473000	3.0249113334000	21.6007118936000
C34	6.1737738534000	3.4938227055000	19.5805266869000
C35	7.5562175745000	3.9221245698000	19.1796642146000
C36	6.4445619637000	2.1746973092000	14.5050521031000
H37	6.8397636451000	3.0516591406000	14.0055539615000

C38	6.2738495267000	0.9557676241000	13.8537788053000
H39	6.5771954188000	0.8609348188000	12.8172049119000
C40	5.6831441375000	-0.1055812193000	14.5337714269000
H41	5.4962596021000	-1.0620375163000	14.0560619639000
C42	5.3091533414000	0.0710855145000	15.8712914432000
C43	5.5657012337000	1.2954445392000	16.4727074310000
H44	5.3235524176000	1.4435447990000	17.5127257421000
C45	4.6170970431000	-1.0433450601000	16.5988701770000
C46	3.5321055123000	-1.7400965461000	18.5850266140000
H47	4.2773568014000	-2.5358497086000	18.6702413467000
H48	2.6809010883000	-2.1425576785000	18.0260657996000
C49	3.1270818064000	-1.2058802838000	19.9531803558000
H50	3.0568698744000	-2.0719062913000	20.6235309687000
H51	3.9445997004000	-0.5853932284000	20.3435351999000
C52	1.4485237999000	0.0083605584000	21.4397023478000
H53	2.2421759581000	0.6661884506000	21.8232355660000
H54	1.4818117956000	-0.8797896948000	22.0835304682000
C55	0.0696464958000	0.6745302621000	21.6202225481000
H56	-0.6923461469000	0.0079463640000	21.1971643574000
H57	-0.1505681290000	0.7394439533000	22.6934179691000
C58	0.6485338998000	3.2041977381000	21.7471560859000
H59	1.6261697275000	2.8420490345000	22.0899678973000
H60	0.0970568433000	3.4484259953000	22.6642239049000
C61	0.8603800427000	4.4823763389000	20.9127253705000
H62	1.5340536737000	5.1613379917000	21.4507402805000
H63	1.3844717043000	4.2308836606000	19.9811400559000
C64	-0.2708080629000	6.3390959530000	19.5102285664000
H65	-1.2458600648000	6.8155017560000	19.3554114183000
H66	0.0168569243000	5.9123661881000	18.5402117375000
C67	0.7267168300000	7.4417423954000	19.8568893695000
H68	0.8087667034000	7.6131396408000	20.9350241362000
H69	0.4588913863000	8.3941506156000	19.3914871086000
C70	2.9969675553000	8.0096536920000	19.5139304740000
C71	4.2968459280000	7.6638572365000	18.8496025140000
C72	5.3963706626000	8.5090085435000	19.0426746622000
H73	5.2756602887000	9.3929206382000	19.6606047992000
C74	6.6094979904000	8.1889659624000	18.4421334398000
H75	7.4848313558000	8.8158988803000	18.5693318581000
C76	6.7069802563000	7.0277791200000	17.6821545593000
H77	7.6372246913000	6.7058117320000	17.2294515098000
C78	4.4568270030000	6.5386051277000	18.0507388353000
H79	3.6229804223000	5.8774680890000	17.8670562207000
H80	-0.8676315833000	5.6473084159000	21.4749333313000
H81	-1.1780090493000	4.5145299404000	20.1749460894000

H82	0.2320376902000	2.0442018079000	19.9519577696000
H83	-1.1618721536000	2.3166048763000	20.9562923676000
H84	0.9915834865000	-1.0822534583000	19.6264299570000
H85	1.8431643479000	0.4160328064000	19.3180122873000

Optimized structure of catalyst 1 of Ru^{III} complex in gas phase

Cartesian coordinates

Ru1	1.3480000000000	-0.0660000000000	-0.1340000000000
O2	0.6680000000000	-0.3390000000000	-2.0340000000000
O3	1.3040000000000	-0.7860000000000	-4.1550000000000
O4	0.2320000000000	0.3110000000000	1.5430000000000
O5	0.3050000000000	0.7520000000000	3.7410000000000
O6	-1.8590000000000	5.5740000000000	-0.7060000000000
O7	-2.4680000000000	3.4680000000000	-0.1610000000000
O8	-4.7400000000000	1.6650000000000	-0.0640000000000
O9	-4.4950000000000	-0.8690000000000	-1.1710000000000
O10	-2.7930000000000	-3.1710000000000	-0.9830000000000
O11	-2.9010000000000	-4.8700000000000	0.4980000000000
N12	2.7480000000000	0.0750000000000	1.3100000000000
N13	3.0540000000000	-0.3840000000000	-1.1410000000000
N14	1.3000000000000	1.9980000000000	-0.4860000000000
N15	0.9470000000000	-2.0940000000000	0.2130000000000
C16	2.3340000000000	2.8160000000000	-0.7580000000000
H17	3.3270000000000	2.3620000000000	-0.7920000000000
C18	2.1560000000000	4.1780000000000	-0.9910000000000
H19	3.0200000000000	4.8060000000000	-1.2120000000000
C20	0.8670000000000	4.7120000000000	-0.9280000000000
H21	0.6700000000000	5.7740000000000	-1.0920000000000
C22	-0.2020000000000	3.8570000000000	-0.6400000000000
C23	0.0520000000000	2.5010000000000	-0.4350000000000
H24	-0.7580000000000	1.7970000000000	-0.2260000000000
C25	-1.5930000000000	4.4120000000000	-0.5210000000000
C26	-3.8330000000000	3.8680000000000	0.0720000000000
H27	-4.3190000000000	4.0640000000000	-0.8960000000000
H28	-3.8410000000000	4.7990000000000	0.6600000000000
C29	-4.5350000000000	2.7560000000000	0.8140000000000
H30	-3.9370000000000	2.4490000000000	1.6930000000000
H31	-5.5070000000000	3.1450000000000	1.1780000000000
C32	-5.3360000000000	0.5390000000000	0.5560000000000
H33	-6.2650000000000	0.8280000000000	1.0850000000000
H34	-4.6440000000000	0.0930000000000	1.2940000000000
C35	-5.6790000000000	-0.4860000000000	-0.5040000000000
H36	-6.1650000000000	-1.3490000000000	-0.0050000000000
H37	-6.4040000000000	-0.0580000000000	-1.2240000000000
C38	-4.5880000000000	-1.9980000000000	-2.0110000000000
H39	-3.9010000000000	-1.8380000000000	-2.8570000000000
H40	-5.6080000000000	-2.1180000000000	-2.4230000000000
C41	-4.1930000000000	-3.2700000000000	-1.2840000000000
H42	-4.3680000000000	-4.1600000000000	-1.9110000000000

H43	-4.7590000000000	-3.4000000000000	-0.3480000000000
C44	-2.2920000000000	-3.9850000000000	-0.0520000000000
C45	-0.8560000000000	-3.6690000000000	0.2410000000000
C46	-0.0730000000000	-4.5720000000000	0.9630000000000
H47	-0.5080000000000	-5.5230000000000	1.2760000000000
C48	1.2440000000000	-4.2210000000000	1.2730000000000
H49	1.8960000000000	-4.9010000000000	1.8230000000000
C50	1.7150000000000	-2.9690000000000	0.8920000000000
H51	2.7270000000000	-2.6450000000000	1.1440000000000
C52	-0.3050000000000	-2.4430000000000	-0.1310000000000
H53	-0.8900000000000	-1.7110000000000	-0.6880000000000
C54	0.8390000000000	0.4880000000000	2.6810000000000
C55	2.3480000000000	0.3400000000000	2.5640000000000
C56	3.2780000000000	0.4580000000000	3.5930000000000
H57	2.9200000000000	0.6750000000000	4.6010000000000
C58	4.6350000000000	0.2930000000000	3.2840000000000
H59	5.3900000000000	0.3790000000000	4.0690000000000
C60	5.0310000000000	0.0200000000000	1.9690000000000
H61	6.0850000000000	-0.1080000000000	1.7210000000000
C62	4.0540000000000	-0.0870000000000	0.9730000000000
C63	4.2330000000000	-0.3660000000000	-0.4650000000000
C64	5.4190000000000	-0.5970000000000	-1.1680000000000
H65	6.3780000000000	-0.5870000000000	-0.6470000000000
C66	5.3570000000000	-0.8410000000000	-2.5470000000000
H67	6.2780000000000	-1.0230000000000	-3.1040000000000
C68	4.1220000000000	-0.8530000000000	-3.2060000000000
H69	4.0180000000000	-1.0390000000000	-4.2760000000000
C70	2.9710000000000	-0.6160000000000	-2.4580000000000
C71	1.5390000000000	-0.5900000000000	-2.9880000000000
O72	-2.1630000000000	0.3290000000000	-0.1050000000000
H73	-2.5470000000000	-0.5320000000000	0.1520000000000
H74	-2.9130000000000	0.7720000000000	-0.5420000000000
O75	-2.4540000000000	0.7250000000000	2.6670000000000
H76	-1.6500000000000	0.8430000000000	3.2060000000000
O77	-2.7990000000000	-1.7570000000000	1.6980000000000
H78	-2.6790000000000	-0.9520000000000	2.2630000000000
H79	-3.5320000000000	-2.2430000000000	2.0940000000000
H80	-2.1440000000000	0.8290000000000	1.7420000000000

Optimized structure of catalyst 2 of Ru^{III} complex in gas phase

Cartesian coordinates

Ru1	1.1980000000000	-0.1470000000000	-0.1320000000000
O2	0.3550000000000	-0.5490000000000	-2.0010000000000
O3	0.8010000000000	-1.3080000000000	-4.0770000000000
O4	0.3130000000000	0.5230000000000	1.6540000000000
O5	0.6790000000000	1.1510000000000	3.7770000000000
O6	-1.3960000000000	5.8070000000000	-0.8510000000000
O7	-2.1350000000000	3.8200000000000	-0.0660000000000
O8	-2.9770000000000	-3.2630000000000	-0.7510000000000
O9	-3.1410000000000	-4.9220000000000	0.7700000000000
N10	2.7520000000000	0.0370000000000	1.1660000000000
N11	2.7830000000000	-0.7190000000000	-1.2400000000000
N12	1.3380000000000	1.8840000000000	-0.6690000000000
N13	0.7130000000000	-2.1250000000000	0.4820000000000
C14	2.4260000000000	2.5340000000000	-1.1270000000000
H15	3.3370000000000	1.9460000000000	-1.2540000000000
C16	2.4010000000000	3.8940000000000	-1.4350000000000
H17	3.3060000000000	4.3760000000000	-1.8080000000000
C18	1.2150000000000	4.6130000000000	-1.2630000000000
H19	1.1430000000000	5.6790000000000	-1.4900000000000
C20	0.0910000000000	3.9340000000000	-0.7850000000000
C21	0.1940000000000	2.5730000000000	-0.5040000000000
H22	-0.6670000000000	2.0040000000000	-0.1610000000000
C23	-1.2190000000000	4.6460000000000	-0.5810000000000
C24	-3.4730000000000	4.3210000000000	0.1730000000000
H25	-3.8780000000000	4.6980000000000	-0.7800000000000
H26	-3.4050000000000	5.1720000000000	0.8680000000000
C27	-4.2940000000000	3.1780000000000	0.7450000000000
H28	-3.7900000000000	2.8040000000000	1.6530000000000
H29	-5.2600000000000	3.5940000000000	1.0790000000000
C30	-5.1820000000000	0.8070000000000	0.4400000000000
H31	-6.0640000000000	1.1350000000000	1.0160000000000
H32	-4.4640000000000	0.4140000000000	1.1800000000000
C33	-5.6000000000000	-0.3350000000000	-0.5020000000000
H34	-5.9770000000000	-1.1700000000000	0.1160000000000
H35	-6.4560000000000	-0.0170000000000	-1.1230000000000
C36	-4.7010000000000	-2.2230000000000	-2.0450000000000
H37	-4.0720000000000	-2.3350000000000	-2.9440000000000
H38	-5.7460000000000	-2.3360000000000	-2.3790000000000
C39	-4.3790000000000	-3.3720000000000	-1.1030000000000
H40	-4.5500000000000	-4.3550000000000	-1.5710000000000
H41	-4.9700000000000	-3.3420000000000	-0.1740000000000
C42	-2.5090000000000	-4.0550000000000	0.2190000000000

C43	-1.1060000000000	-3.6870000000000	0.6100000000000
C44	-0.5020000000000	-4.3320000000000	1.6920000000000
H45	-1.0210000000000	-5.1610000000000	2.1760000000000
C46	0.7390000000000	-3.8700000000000	2.1340000000000
H47	1.2560000000000	-4.3430000000000	2.9700000000000
C48	1.2980000000000	-2.7510000000000	1.5240000000000
H49	2.2360000000000	-2.3310000000000	1.8910000000000
C50	-0.4420000000000	-2.6220000000000	0.0030000000000
H51	-0.8660000000000	-2.1280000000000	-0.8650000000000
C52	1.0590000000000	0.7520000000000	2.6880000000000
C53	2.5240000000000	0.4730000000000	2.4160000000000
C54	3.5670000000000	0.6240000000000	3.3250000000000
H55	3.3340000000000	0.9810000000000	4.3290000000000
C56	4.8650000000000	0.3090000000000	2.9070000000000
H57	5.7070000000000	0.4150000000000	3.5950000000000
C58	5.0870000000000	-0.1460000000000	1.6020000000000
H59	6.0930000000000	-0.3960000000000	1.2640000000000
C60	4.0000000000000	-0.2770000000000	0.7310000000000
C61	4.0170000000000	-0.7310000000000	-0.6690000000000
C62	5.1190000000000	-1.1400000000000	-1.4250000000000
H63	6.1150000000000	-1.1550000000000	-0.9790000000000
C64	4.9290000000000	-1.5290000000000	-2.7570000000000
H65	5.7840000000000	-1.8510000000000	-3.3550000000000
C66	3.6460000000000	-1.5020000000000	-3.3130000000000
H67	3.4340000000000	-1.7920000000000	-4.3440000000000
C68	2.5840000000000	-1.0850000000000	-2.5140000000000
C69	1.1320000000000	-0.9920000000000	-2.9590000000000
O70	-1.4400000000000	0.1870000000000	-0.2100000000000
H71	-1.9150000000000	-0.3490000000000	0.4800000000000
H72	-1.7450000000000	-0.0970000000000	-1.0820000000000
O73	-2.1060000000000	1.3170000000000	2.9640000000000
H74	-1.3110000000000	1.3290000000000	3.5350000000000
O75	-2.4760000000000	-1.0980000000000	1.8710000000000
H76	-2.3710000000000	-0.2860000000000	2.4380000000000
H77	-3.3960000000000	-1.3690000000000	1.9890000000000
H78	-1.7430000000000	1.6210000000000	2.1180000000000
C79	-4.4760000000000	-0.8370000000000	-1.4190000000000
H80	-3.5450000000000	-0.8850000000000	-0.8390000000000
H81	-4.3010000000000	-0.1010000000000	-2.2190000000000
C82	-4.5330000000000	2.0210000000000	-0.2360000000000
H83	-3.5660000000000	1.7220000000000	-0.6700000000000
H84	-5.1630000000000	2.3720000000000	-1.0740000000000

Optimized structure of catalyst 1 of Ru^{III} complex with PCM solvation model

Cartesian coordinates

Ru1	1.3480000000000	-0.0500000000000	-0.1420000000000
O2	0.7490000000000	-0.3120000000000	-2.0980000000000
O3	1.4670000000000	-0.7640000000000	-4.1800000000000
O4	0.1640000000000	0.2660000000000	1.5160000000000
O5	0.1610000000000	0.7550000000000	3.6990000000000
O6	-1.9120000000000	5.5670000000000	-0.7930000000000
O7	-2.5190000000000	3.4560000000000	-0.2830000000000
O8	-4.8050000000000	1.6210000000000	-0.1600000000000
O9	-4.5750000000000	-0.9800000000000	-1.1240000000000
O10	-2.7840000000000	-3.2210000000000	-0.8400000000000
O11	-2.7140000000000	-5.0480000000000	0.4740000000000
N12	2.6940000000000	0.1150000000000	1.3480000000000
N13	3.0940000000000	-0.3350000000000	-1.0950000000000
N14	1.2770000000000	2.0200000000000	-0.4540000000000
N15	1.0040000000000	-2.0900000000000	0.1580000000000
C16	2.3150000000000	2.8500000000000	-0.6690000000000
H17	3.3130000000000	2.4080000000000	-0.6680000000000
C18	2.1350000000000	4.2140000000000	-0.8880000000000
H19	3.0040000000000	4.8490000000000	-1.0620000000000
C20	0.8420000000000	4.7370000000000	-0.8770000000000
H21	0.6530000000000	5.8000000000000	-1.0400000000000
C22	-0.2330000000000	3.8710000000000	-0.6440000000000
C23	0.0240000000000	2.5160000000000	-0.4400000000000
H24	-0.7890000000000	1.8090000000000	-0.2610000000000
C25	-1.6320000000000	4.4070000000000	-0.5940000000000
C26	-3.8970000000000	3.8420000000000	-0.1160000000000
H27	-4.3550000000000	3.9810000000000	-1.1090000000000
H28	-3.9440000000000	4.7970000000000	0.4270000000000
C29	-4.6110000000000	2.7620000000000	0.6600000000000
H30	-4.0250000000000	2.5010000000000	1.5610000000000
H31	-5.5890000000000	3.1630000000000	0.9900000000000
C32	-5.4020000000000	0.5300000000000	0.5290000000000
H33	-6.3270000000000	0.8530000000000	1.0430000000000
H34	-4.7050000000000	0.1380000000000	1.2910000000000
C35	-5.7510000000000	-0.5630000000000	-0.4580000000000
H36	-6.2190000000000	-1.3930000000000	0.1050000000000
H37	-6.4940000000000	-0.1900000000000	-1.1910000000000
C38	-4.6710000000000	-2.1840000000000	-1.8570000000000
H39	-4.0370000000000	-2.0800000000000	-2.7530000000000
H40	-5.7050000000000	-2.3710000000000	-2.1990000000000
C41	-4.1970000000000	-3.3780000000000	-1.0530000000000
H42	-4.3820000000000	-4.3190000000000	-1.5960000000000

H43	-4.7080000000000	-3.4400000000000	-0.0800000000000
C44	-2.1770000000000	-4.0850000000000	-0.0260000000000
C45	-0.7390000000000	-3.7320000000000	0.1960000000000
C46	0.1380000000000	-4.6630000000000	0.7600000000000
H47	-0.2240000000000	-5.6620000000000	1.0120000000000
C48	1.4630000000000	-4.2890000000000	0.9880000000000
H49	2.1860000000000	-4.9860000000000	1.4130000000000
C50	1.8600000000000	-2.9900000000000	0.6810000000000
H51	2.8810000000000	-2.6540000000000	0.8660000000000
C52	-0.2630000000000	-2.4560000000000	-0.1010000000000
H53	-0.9220000000000	-1.7010000000000	-0.5290000000000
C54	0.7440000000000	0.4830000000000	2.6570000000000
C55	2.2520000000000	0.3760000000000	2.5880000000000
C56	3.1480000000000	0.5220000000000	3.6410000000000
H57	2.7660000000000	0.7350000000000	4.6400000000000
C58	4.5160000000000	0.3880000000000	3.3730000000000
H59	5.2460000000000	0.4960000000000	4.1780000000000
C60	4.9560000000000	0.1170000000000	2.0730000000000
H61	6.0190000000000	0.0130000000000	1.8550000000000
C62	4.0110000000000	-0.0170000000000	1.0520000000000
C63	4.2450000000000	-0.2960000000000	-0.3770000000000
C64	5.4620000000000	-0.5120000000000	-1.0290000000000
H65	6.3970000000000	-0.4840000000000	-0.4700000000000
C66	5.4580000000000	-0.7640000000000	-2.4050000000000
H67	6.4020000000000	-0.9340000000000	-2.9250000000000
C68	4.2500000000000	-0.7970000000000	-3.1120000000000
H69	4.2020000000000	-0.9890000000000	-4.1840000000000
C70	3.0680000000000	-0.5740000000000	-2.4130000000000
C71	1.6660000000000	-0.5620000000000	-2.9970000000000
O72	-2.2490000000000	0.3400000000000	-0.1310000000000
H73	-2.5780000000000	-0.5220000000000	0.1930000000000
H74	-3.0450000000000	0.7550000000000	-0.5110000000000
O75	-2.4940000000000	0.8300000000000	2.6370000000000
H76	-1.6450000000000	0.8870000000000	3.1220000000000
O77	-2.7970000000000	-1.7180000000000	1.7370000000000
H78	-2.7050000000000	-0.8910000000000	2.2690000000000
H79	-3.6770000000000	-2.0520000000000	1.9570000000000
H80	-2.2240000000000	0.8900000000000	1.6950000000000

Optimized structure of catalyst 2 of Ru^{III} complex with PCM solvation model

Cartesian coordinates

Ru1	1.3372610888931	-0.0579363651508	-0.1454347119413
O2	0.7615418788480	-0.3168455069099	-2.1357945908322
O3	1.5151248985920	-0.7709681638454	-4.2033572246625
O4	0.1639555355413	0.2755626132589	1.5561211965946
O5	0.1929601644928	0.7990352162563	3.7303569624237
O6	-1.7368928854928	5.7160931134999	-0.3288520541970
O7	-2.3841844330800	3.6014477574973	0.1136484212366
O8	-2.6005581871719	-3.4332890016759	-1.0447872168883
O9	-2.4096626840387	-5.4240700251128	-0.0050681088562
N10	2.6902447966084	0.1126143368522	1.3525617983027
N11	3.0922414967176	-0.3317807411982	-1.0938918938211
N12	1.3191987835437	2.0372190963142	-0.3814227315924
N13	1.0582321316205	-2.1355745008994	0.1398171379853
C14	2.3876970027952	2.8354454894835	-0.5660471630855
H15	3.3620496612639	2.3542167982245	-0.6618273470699
C16	2.2704037409624	4.2237736347227	-0.6336951627995
H17	3.1638775689638	4.8288978136844	-0.7848382959967
C18	1.0099475840446	4.8103081509109	-0.5033972817432
H19	0.8746528323562	5.8921240273628	-0.5496870173460
C20	-0.0972617980498	3.9758457293456	-0.3109372827882
C21	0.1018034841775	2.5979195241273	-0.2614864346209
H22	-0.7350447022214	1.9116222552051	-0.1512268699980
C23	-1.4784831484087	4.5421376288219	-0.1805721574661
C24	-3.7749419721429	4.0039994156743	0.1968627832109
H25	-4.0791861781745	4.3973269190026	-0.7866040124775
H26	-3.8540489889581	4.8236314015690	0.9273629458317
C27	-4.6021708571395	2.8002243035357	0.6130392496563
H28	-4.1983255977316	2.4125498567709	1.5633899900566
H29	-5.6185592368215	3.1663621302791	0.8398006958393
C30	-5.3388055396817	0.4109313704109	0.1465163411128
H31	-6.2944099639695	0.6857009619941	0.6268900038200
H32	-4.6889374299534	0.0323127541380	0.9527777028124
C33	-5.5949962127670	-0.7307812442483	-0.8523353218244
H34	-5.9763275151692	-1.5998614519357	-0.2873211357952
H35	-6.4032848239388	-0.4481051506424	-1.5516762595319
C36	-4.4284637665611	-2.5407355281505	-2.3051627520902
H37	-3.7822575110588	-2.5777019932963	-3.1985405869419
H38	-5.4519879762002	-2.7620571580903	-2.6505913675862
C39	-3.9909670304337	-3.6645536300441	-1.3778141230762
H40	-4.0829460178004	-4.6533129498394	-1.8527413526608
H41	-4.5691162806366	-3.6848855000505	-0.4390915551405
C42	-1.9451185367162	-4.3566323262113	-0.3397283756229

C43	-0.5593754240609	-3.9083302631351	0.0222637319844
C44	0.2987992465034	-4.7570904459668	0.7286888071631
H45	-0.0234328512238	-5.7706931799197	0.9756502877979
C46	1.5518007652906	-4.2741679576227	1.1091767362341
H47	2.2601779448278	-4.8998675774840	1.6521157357693
C48	1.8915367647265	-2.9563236300487	0.8102858214037
H49	2.8516781757083	-2.5464672112138	1.1259948438560
C50	-0.1272543740013	-2.6159549785234	-0.2715942640277
H51	-0.7612724132931	-1.9339640622913	-0.8222797605041
C52	0.7566871874839	0.5001506709587	2.6804189299037
C53	2.2600773953151	0.3798169961193	2.5962602126078
C54	3.1623182882597	0.5279325671846	3.6453117598191
H55	2.7819140914244	0.7462678513321	4.6430333395829
C56	4.5278580070789	0.3911899550135	3.3722774499900
H57	5.2615329135807	0.4971780667434	4.1733677393715
C58	4.9578941974465	0.1190935321163	2.0704581800371
H59	6.0191303187258	0.0139915375123	1.8451018718884
C60	4.0066802351451	-0.0143241780657	1.0553272956027
C61	4.2415491710158	-0.2898826760814	-0.3727882799493
C62	5.4648576057030	-0.4932809112879	-1.0157094504967
H63	6.3943273696722	-0.4570927186505	-0.4485301379503
C64	5.4753986859704	-0.7407064421911	-2.3919545203458
H65	6.4243214091984	-0.9006354183377	-2.9056746658825
C66	4.2715867081595	-0.7799488497309	-3.1051065066219
H67	4.2294174202571	-0.9685879241407	-4.1777733005349
C68	3.0828326176622	-0.5686301788190	-2.4132234734073
C69	1.6906816197843	-0.5648354418302	-3.0165336747787
O70	-1.4509576767196	0.1657653030619	-0.5837093732260
H71	-1.9224079040581	-0.3948486852410	0.0844220803529
H72	-1.7390110196857	-0.1033414779318	-1.4647228873274
O73	-2.3810897734496	1.1746920756956	2.7286230451909
H74	-1.5370089124338	1.0850779821086	3.2277691303177
O75	-2.4692349972810	-1.2272499994020	1.4576180540947
H76	-2.4845781668795	-0.4177943253598	2.0313489133898
H77	-3.3485796741150	-1.6208862822460	1.5400281029508
H78	-2.1121145339147	1.6751371797051	1.9429093451766
C79	-4.3566797892162	-1.1474136256141	-1.6584586957331
H80	-3.4865518286928	-1.1351305672387	-0.9920418151002
H81	-4.1582108686808	-0.3927881134840	-2.4357356245196
C82	-4.6745252531114	1.6710291185979	-0.4243421053414
H83	-3.6526366203712	1.4266080104770	-0.7536549416859
H84	-5.2226744761216	2.0256441675911	-1.3167666659295

Optimized preactive structure of catalyst 1 of Ru^V complex via I2M mechanism

E(B3LYP-D3/LACV3P**++) (a.u.) = -4599.492183

Solvation energy (kcal mol^{-1}) = -137.455

Zero-point energy (kcal mol^{-1}) = 684.18

ΔH_{298} (kcal mol^{-1}) = 51.352

ΔS_{298} (cal K^{-1} mol^{-1}) = 420.821

Cartesian coordinates

Ru1	-0.9333555176000	0.2350528822000	-5.9609085539000
O2	-4.2495812000000	5.8668466724000	-6.6717650245000
O3	-4.7193467258000	3.9047538753000	-5.5966727222000
O4	-6.3387220236000	1.6448919037000	-5.6976108219000
O5	-6.1018847478000	-1.8608168423000	-6.4563400303000
O6	-4.1846241665000	-3.9206057371000	-6.7448529411000
O7	-3.7142876260000	-5.6053285624000	-5.2815510129000
O8	-2.9366169952000	0.1515893417000	-6.7654146787000
O9	-4.2466107679000	0.5333331998000	-8.5673723019000
O10	0.4220893510000	0.7201279682000	-4.3876713466000
O11	2.5031476951000	1.0760910718000	-3.6102992191000
N12	-1.0826659953000	2.3461597692000	-6.2366100780000
N13	-0.8466471137000	-1.8864430099000	-5.7207082351000
N14	1.1935338569000	0.0673726234000	-6.6973050713000
N15	-0.8427113227000	-0.1344915665000	-8.1739571592000
C16	2.1872156415000	0.2617618776000	-5.8214452349000
C17	3.5244819161000	0.0491674887000	-6.1342224092000
H18	4.2660791057000	0.2416055135000	-5.3665237610000
C19	3.8365346467000	-0.4071139825000	-7.4124353318000
H20	4.8654703229000	-0.6038160088000	-7.6968729617000
C21	2.8057562432000	-0.6003650136000	-8.3320233834000
H22	3.0243597906000	-0.9419992904000	-9.3369451035000
C23	1.4870486111000	-0.3428503218000	-7.9504446648000
C24	0.3123422473000	-0.4345151316000	-8.8121051767000
C25	0.3249218937000	-0.7508317966000	-10.1701293262000
H26	1.2577707334000	-1.0015739317000	-10.6616490293000
C27	-0.8700885051000	-0.7249710327000	-10.8905089489000
H28	-0.8746120146000	-0.9647266146000	-11.9490249620000
C29	-2.0455297884000	-0.3779234453000	-10.2322426866000
H30	-3.0106516232000	-0.3162369101000	-10.7228665510000
C31	-1.9846330250000	-0.1069359868000	-8.8685520906000
C32	1.7181252706000	0.7291386796000	-4.4799576545000
C33	-3.1869903012000	0.2309320338000	-8.0452855807000
C34	-0.0143359012000	3.1579008359000	-6.3724351383000
H35	0.9620739201000	2.6930585707000	-6.3697635148000
C36	-0.1528605821000	4.5353131202000	-6.5021331464000
H37	0.7343562996000	5.1501957055000	-6.5953336342000

C38	-1.4298078131000	5.0937803779000	-6.4810457996000
H39	-1.5909522842000	6.1614082105000	-6.5984888768000
C40	-2.5254387346000	4.2522738683000	-6.2758549118000
C41	-2.3163571107000	2.8808715345000	-6.1601675669000
H42	-3.1430813102000	2.1983273495000	-6.0135210863000
C43	-3.9236147787000	4.7942480976000	-6.2168790465000
C44	-6.1618997447000	4.0225872256000	-5.7108816304000
H45	-6.4163117610000	4.9456896153000	-6.2372291804000
H46	-6.5513914089000	4.0546089760000	-4.6902930783000
C47	-6.6666702943000	2.7972389928000	-6.4498628816000
H48	-7.7568860224000	2.8937378241000	-6.5909116006000
H49	-6.2020785706000	2.7563767385000	-7.4485284816000
C50	-6.6431336425000	0.4378789408000	-6.3955642149000
H51	-6.1576220536000	0.4286585495000	-7.3769277670000
H52	-7.7299083240000	0.3328643674000	-6.5402240659000
C53	-6.1001555533000	-0.7338453257000	-5.5865522234000
H54	-5.0784663132000	-0.5113456286000	-5.2523743847000
H55	-6.7277871935000	-0.9106279375000	-4.6987227967000
C56	-6.2742210178000	-3.1255403003000	-5.8412906386000
H57	-7.3445175169000	-3.3708774384000	-5.7355196964000
H58	-5.8093566632000	-3.1571057607000	-4.8484809284000
C59	-5.6143469300000	-4.1719912620000	-6.7238458403000
H60	-5.9425846097000	-4.0712477791000	-7.7602315553000
H61	-5.8070770049000	-5.1839803329000	-6.3604271646000
C62	-3.4085367985000	-4.6036022590000	-5.8914714609000
C63	-2.0776756930000	-3.9341910717000	-5.7343220475000
C64	-1.0068608953000	-4.5985901669000	-5.1428128220000
H65	-1.1066981844000	-5.6505136294000	-4.8930124591000
C66	0.1524124837000	-3.8776693272000	-4.8537161684000
H67	1.0087699813000	-4.3491510597000	-4.3839643884000
C68	0.1908814916000	-2.5192922299000	-5.1324154990000
H69	1.0417323332000	-1.9141706158000	-4.8553886638000
C70	-1.9603404756000	-2.5759135214000	-6.0164143374000
H71	-2.7979328919000	-2.0240615793000	-6.4170596071000
O72	-1.9583814084000	0.3405896720000	-4.5643030657000
Ru73	-0.8135237566000	-0.9098065354000	-1.1831768412000
O74	1.6335371390000	4.3554811723000	-3.7906214612000
O75	2.2624538648000	3.4352809419000	-1.8075416709000
O76	4.2404987168000	1.5362685109000	-1.0434888281000
O77	5.4322446046000	-1.3400259900000	0.7540992559000
O78	3.3796879072000	-3.5362052103000	0.8428845167000
O79	2.8806300089000	-5.6135682038000	1.6303630824000
O80	1.0036852663000	-0.2791094642000	-0.2561301416000
O81	2.1397758101000	0.4303706073000	1.5507899998000

O82	-1.8863775121000	-1.8591331135000	-2.8124747697000
O83	-3.8283189541000	-2.3591614076000	-3.8398974970000
N84	-1.0904148908000	1.1427419222000	-1.7757139557000
N85	-0.4084756309000	-2.8764308982000	-0.4652934882000
N86	-3.0159622655000	-1.0286708392000	-0.6955906259000
N87	-1.2419219543000	-0.2411149914000	0.9205424271000
C88	-3.8573722320000	-1.4317591841000	-1.6585632613000
C89	-5.2384140644000	-1.4296405627000	-1.5004490426000
H90	-5.8474067967000	-1.7644642408000	-2.3326239796000
C91	-5.7674054974000	-0.9979953218000	-0.2861324620000
H92	-6.8402778786000	-0.9769468985000	-0.1238103645000
C93	-4.8964173399000	-0.5950942522000	0.7256355946000
H94	-5.2805497274000	-0.2581174638000	1.6807883682000
C95	-3.5193251062000	-0.6191207379000	0.4906821413000
C96	-2.4916999226000	-0.2028698758000	1.4380064689000
C97	-2.7247019427000	0.2160402204000	2.7488467845000
H98	-3.7327927178000	0.2334565322000	3.1450305754000
C99	-1.6450760672000	0.6106436423000	3.5402038467000
H100	-1.8063063438000	0.9372331406000	4.5624407371000
C101	-0.3660672903000	0.5853561715000	2.9933505272000
H102	0.5239493232000	0.8867389469000	3.5351890792000
C103	-0.2155130043000	0.1567749687000	1.6780711193000
C104	-3.1752576257000	-1.9218609724000	-2.8987166363000
C105	1.1084394772000	0.1201983378000	0.9840897502000
C106	-2.2522800888000	1.6824427013000	-2.2039332720000
H107	-3.1407837207000	1.0727555235000	-2.1175375042000
C108	-2.3114814941000	2.9471322460000	-2.7759652449000
H109	-3.2623253964000	3.3248515370000	-3.1373934023000
C110	-1.1290835640000	3.6679962347000	-2.9481967231000
H111	-1.1190923724000	4.6276083337000	-3.4553445893000
C112	0.0614203647000	3.1175633600000	-2.4845507593000
C113	0.0445505086000	1.8548987522000	-1.8955149099000
H114	0.9703193133000	1.3874666640000	-1.5896311489000
C115	1.3977425040000	3.7300283375000	-2.7738433005000
C116	3.6771871614000	3.6109517790000	-2.0749464452000
H117	3.9054920528000	4.6802621983000	-2.1177493857000
H118	3.9019945362000	3.1444666338000	-3.0366755953000
C119	4.4298677608000	2.9317185032000	-0.9495268667000
H120	5.4973027320000	3.1982575456000	-1.0405027696000
H121	4.0757028760000	3.3190486555000	0.0200084336000
C122	4.9048715543000	0.8209281646000	-0.0110860389000
H123	4.5103916141000	1.0942799714000	0.9763440432000
H124	5.9876042073000	1.0245914490000	-0.0229955869000
C125	4.6797160318000	-0.6668723119000	-0.2481050745000

H126	3.6086080270000	-0.8940237034000	-0.1848317753000
H127	5.0345546985000	-0.9345852472000	-1.2561638329000
C128	5.6508355556000	-2.7232129284000	0.5739537957000
H129	6.6733584697000	-2.9381607142000	0.9121477894000
H130	5.5821563423000	-3.0140560077000	-0.4852961643000
C131	4.7242899220000	-3.5964389496000	1.4032448662000
H132	4.6697531383000	-3.2309849217000	2.4332657054000
H133	5.0601599352000	-4.6371685880000	1.4079935693000
C134	2.5790795135000	-4.5892769380000	1.0580853206000
C135	1.1999571955000	-4.3501425551000	0.5125531557000
C136	0.2312855896000	-5.3511191194000	0.6175023363000
H137	0.5018162799000	-6.3017502715000	1.0671798163000
C138	-1.0539455492000	-5.0968128183000	0.1405813737000
H139	-1.8311492962000	-5.8520667381000	0.1857885871000
C140	-1.3428336373000	-3.8461600340000	-0.3927196172000
H141	-2.3267227631000	-3.6038045057000	-0.7717533738000
C142	0.8485265920000	-3.1296874699000	-0.0524714898000
H143	1.5720150992000	-2.3350174113000	-0.1666691734000
O144	0.3778553547000	-1.1407190583000	-2.4200733415000

Optimized transition state structure of catalyst 1 of Ru^V complex via I2M mechanism

E(B3LYP-D3/LACV3P**++) (a.u.) = -4599.471837

Solvation energy (kcal mol^{-1}) = -137.207

Zero-point energy (kcal mol^{-1}) = 683.544

ΔH_{298} (kcal mol^{-1}) = 50.905

ΔS_{298} (cal K^{-1} mol^{-1}) = 415.728

Cartesian coordinates

Ru1	-0.4090232379000	0.5349375437000	-6.0578772823000
O2	-4.1079190000000	5.7587148667000	-7.5577400165000
O3	-4.5125060218000	3.8256759249000	-6.4153979406000
O4	-6.2111690050000	1.5150421430000	-6.4693685436000
O5	-6.0679318069000	-2.0235961131000	-7.1496259734000
O6	-3.7198483930000	-3.5854842565000	-6.3774461886000
O7	-3.3371207715000	-5.1545345638000	-4.7615938121000
O8	-2.4739257950000	0.3532588758000	-6.6106064450000
O9	-3.8837847898000	0.8549476169000	-8.3092882832000
O10	1.0988145017000	1.2846639485000	-4.7199953636000
O11	3.2179653012000	2.0448138900000	-4.4870209148000
N12	-0.6871374535000	2.5928695384000	-6.5811630793000
N13	-0.3456856739000	-1.5355484489000	-5.5612477498000
N14	1.6082250195000	0.2625236516000	-6.9925100019000
N15	-0.5600682019000	-0.1182879934000	-8.1953237776000
C16	2.6843925941000	0.6081746867000	-6.2821725227000
C17	3.9822105811000	0.3110737133000	-6.6869629349000
H18	4.8027543501000	0.6537033701000	-6.0661159707000
C19	4.1559469289000	-0.4078361419000	-7.8659657240000
H20	5.1482637241000	-0.6850923224000	-8.2077337905000
C21	3.0333413335000	-0.7489810370000	-8.6227244792000
H22	3.1441285315000	-1.2826451948000	-9.5591957654000
C23	1.7656158072000	-0.3818372261000	-8.1724374726000
C24	0.5096767196000	-0.5579931850000	-8.8934173655000
C25	0.3661885584000	-1.0441771395000	-10.1930336854000
H26	1.2302885339000	-1.4091935222000	-10.7365447139000
C27	-0.8944885610000	-1.0277855780000	-10.7926598520000
H28	-1.0203950252000	-1.3955775962000	-11.8061195039000
C29	-1.9772902207000	-0.5172242560000	-10.0809114616000
H30	-2.9790837681000	-0.4437714360000	-10.4893622502000
C31	-1.7622783598000	-0.0943944887000	-8.7734167565000
C32	2.3544878712000	1.3884527705000	-5.0463204576000
C33	-2.8334263431000	0.4254712009000	-7.8702601001000
C34	0.2947684559000	3.4095227207000	-7.0145232802000
H35	1.3003489833000	3.0077766350000	-7.0189242977000
C36	0.0304830025000	4.7035336364000	-7.4473470897000
H37	0.8483198637000	5.3310847933000	-7.7833173615000

C38	-1.2846248267000	5.1663950705000	-7.4502125218000
H39	-1.5444082274000	6.1536894307000	-7.8190920530000
C40	-2.2904933457000	4.3273062465000	-6.9663928769000
C41	-1.9472769058000	3.0604775243000	-6.5107008168000
H42	-2.7018173454000	2.3910379728000	-6.1261358463000
C43	-3.7311322355000	4.7430104959000	-7.0181962370000
C44	-5.9531067719000	3.8947565556000	-6.5903326164000
H45	-6.2070965013000	4.7915091001000	-7.1599605363000
H46	-6.3854298576000	3.9600236682000	-5.5876532330000
C47	-6.4276463923000	2.6401801230000	-7.2986158347000
H48	-7.4988885204000	2.7675494158000	-7.5331845507000
H49	-5.8819312249000	2.5329720468000	-8.2477543819000
C50	-6.5520111142000	0.2914326861000	-7.1187609975000
H51	-6.1555727811000	0.2685395497000	-8.1401981813000
H52	-7.6454145615000	0.1707214767000	-7.1736179930000
C53	-5.9296133058000	-0.8605482583000	-6.3369274231000
H54	-4.8739000369000	-0.6362996725000	-6.1309969283000
H55	-6.4393186797000	-0.9859473056000	-5.3731519970000
C56	-6.1571133067000	-3.2749872274000	-6.4836287428000
H57	-7.0581897834000	-3.7957396438000	-6.8409470799000
H58	-6.2344418820000	-3.1530238438000	-5.4021345300000
C59	-4.9763045500000	-4.1763268251000	-6.8128672867000
H60	-4.8718942075000	-4.2877657188000	-7.8953682011000
H61	-5.0968041871000	-5.1567014174000	-6.3447475822000
C62	-3.0183166806000	-4.1742249869000	-5.3990615896000
C63	-1.7028780262000	-3.4738011744000	-5.2024518570000
C64	-0.6770250768000	-4.0608582515000	-4.4623754875000
H65	-0.8363170488000	-5.0390604363000	-4.0186994604000
C66	0.5255719335000	-3.3673349868000	-4.3048730923000
H67	1.3436128304000	-3.7755995202000	-3.7231048038000
C68	0.6552961033000	-2.0990811378000	-4.8554461714000
H69	1.5407860365000	-1.4943923955000	-4.6992741887000
C70	-1.4949115376000	-2.2093308745000	-5.7384892079000
H71	-2.2866956417000	-1.7051188464000	-6.2678273793000
O72	-1.2611724658000	0.8682393569000	-4.5273834810000
Ru73	-2.1170528142000	-0.2332604688000	-1.7596095970000
O74	0.2799540203000	5.0973423431000	-4.1939890444000
O75	0.8509733544000	4.1176616077000	-2.2166361535000
O76	2.6233734359000	2.0513489922000	-1.6900945479000
O77	4.3532898470000	-1.1005601357000	-1.5371511896000
O78	2.2661807631000	-3.2725335210000	-1.3581578607000
O79	1.9290702571000	-5.2903558371000	-0.3689420658000
O80	-0.1463914456000	0.1885720680000	-1.0659553171000
O81	1.2552880188000	0.5581481335000	0.6621276623000

O82	-3.3892306635000	-1.0826348771000	-3.2568322634000
O83	-5.2360361122000	-2.3165226221000	-3.6324809691000
N84	-2.4304671629000	1.7832522994000	-2.3194353099000
N85	-1.6616978699000	-2.2563708422000	-1.1821511957000
N86	-4.1999094922000	-0.3165049626000	-0.9757568926000
N87	-2.2237519026000	0.5429367703000	0.3323654805000
C88	-5.1400585043000	-0.8819121821000	-1.7436353992000
C89	-6.4866588381000	-0.8816506458000	-1.3992337599000
H90	-7.1862375756000	-1.3808635685000	-2.0605561032000
C91	-6.8687183834000	-0.2311698922000	-0.2272164498000
H92	-7.9129918634000	-0.1862704487000	0.0655145080000
C93	-5.8902474430000	0.3555157085000	0.5768622300000
H94	-6.1612418389000	0.8536219536000	1.5001417816000
C95	-4.5515516701000	0.2825602585000	0.1853444000000
C96	-3.4068218465000	0.7750337155000	0.9455843447000
C97	-3.4601520236000	1.3874236004000	2.1984697185000
H98	-4.4155881653000	1.5786124218000	2.6725379888000
C99	-2.2681050027000	1.7360975627000	2.8364920601000
H100	-2.2904946843000	2.2118856157000	3.8117213049000
C101	-1.0569167669000	1.4519687663000	2.2116909321000
H102	-0.0904408686000	1.6688245359000	2.6536033894000
C103	-1.0845957301000	0.8654666235000	0.9504033302000
C104	-4.5874268400000	-1.5049643831000	-2.9909127536000
C105	0.1443694455000	0.5240801107000	0.1665637371000
C106	-3.5815148151000	2.2525771298000	-2.8414660831000
H107	-4.4362962601000	1.5886514097000	-2.8223938125000
C108	-3.6550161251000	3.5118302787000	-3.4241736158000
H109	-4.5912419545000	3.8481970822000	-3.8508077227000
C110	-2.4960839828000	4.2818930796000	-3.5268952231000
H111	-2.4945309910000	5.2393648022000	-4.0383265185000
C112	-1.3106104217000	3.7837507644000	-2.9886827848000
C113	-1.3175928160000	2.5358239581000	-2.3770484962000
H114	-0.3970825516000	2.0987828571000	-2.0145387774000
C115	0.0197922253000	4.4369377912000	-3.2083975560000
C116	2.2762756515000	4.3069176055000	-2.4177194832000
H117	2.5259220523000	5.3567686406000	-2.2333603939000
H118	2.5224258978000	4.0357986281000	-3.4444164027000
C119	2.9880218764000	3.3943549869000	-1.4375790897000
H120	4.0683402299000	3.5514421203000	-1.5835996933000
H121	2.7478471166000	3.6751309951000	-0.3994740600000
C122	3.6191116179000	1.1187985804000	-1.3024497321000
H123	3.7075937499000	1.0599012357000	-0.2090914571000
H124	4.5977631272000	1.4012034809000	-1.7194095837000
C125	3.2490881442000	-0.2549391958000	-1.8486832273000

H126	2.3172142726000	-0.6056954581000	-1.3955449550000
H127	3.1076036990000	-0.1891698384000	-2.9391557707000
C128	4.3697195382000	-2.3769855393000	-2.1304634233000
H129	5.4236645992000	-2.6590633119000	-2.2453501297000
H130	3.9180979046000	-2.3632086556000	-3.1380368696000
C131	3.7078311589000	-3.4593595907000	-1.2934104585000
H132	4.0203711154000	-3.3859547924000	-0.2481997352000
H133	3.9518719430000	-4.4591374602000	-1.6661782781000
C134	1.5101496434000	-4.2465460367000	-0.8158166342000
C135	0.0564316884000	-3.8864285346000	-0.8375272889000
C136	-0.8971102459000	-4.8372348311000	-0.4670532463000
H137	-0.5713162523000	-5.8304313956000	-0.1733268426000
C138	-2.2428161147000	-4.4772222572000	-0.4916334086000
H139	-3.0218967668000	-5.1862487468000	-0.2341991258000
C140	-2.5878245548000	-3.1792729934000	-0.8492165048000
H141	-3.6230750631000	-2.8669852055000	-0.8639969470000
C142	-0.3636312579000	-2.6108494086000	-1.2014209759000
H143	0.3428941539000	-1.8499590852000	-1.4986830992000
O144	-1.0807094319000	-0.4807030345000	-3.2015678613000

Optimized preactive structure of catalyst 1 of Ru^{V} complex via WNA mechanism

E(B3LYP-D3/LACV3P**++) (a.u.) = -2605.648714

Solvation energy (kcal mol^{-1}) = -67.102

Zero-point energy (kcal mol^{-1}) = 405.874

ΔH_{298} (kcal mol^{-1}) = 31.885

ΔS_{298} (cal K^{-1} mol^{-1}) = 279.428

Cartesian coordinates

Ru1	1.1630851234000	0.0040828745000	0.0129053148000
O2	-2.2254944208000	5.5599502365000	0.9779507988000
O3	-2.6270196445000	3.6274538359000	-0.1487068546000
O4	-4.7753788959000	1.7122441975000	-0.3538017950000
O5	-4.2524614241000	-1.2087666719000	0.3701642391000
O6	-2.5456217293000	-3.5479086651000	0.4784704738000
O7	-2.3703781359000	-5.3815970609000	-0.8516669881000
O8	0.6305544307000	0.5456907174000	-1.9984595462000
O9	1.1051139531000	1.8449645693000	-3.7783449976000
O10	0.7371384755000	-0.3751284456000	2.0438548758000
O11	1.1004920475000	-1.7884732272000	3.7550855989000
N12	1.0169902412000	2.0703227945000	0.5598171501000
N13	1.0319554431000	-2.0728800136000	-0.4760342582000
N14	3.0438009982000	-0.5968636882000	1.0961059044000
N15	3.0185536501000	0.5235447979000	-1.1538236394000
C16	2.8785682311000	-1.2367514104000	2.2582911253000
C17	3.9381354551000	-1.8214798164000	2.9432411485000
H18	3.7302149230000	-2.3337299038000	3.8760172624000
C19	5.2170154037000	-1.7044341807000	2.4013013953000
H20	6.0729555767000	-2.1426439445000	2.9047734970000
C21	5.3929658029000	-1.0073818160000	1.2044909775000
H22	6.3798627901000	-0.8964277196000	0.7697354392000
C23	4.2771329695000	-0.4646912884000	0.5644624925000
C24	4.2689875157000	0.2729413522000	-0.6989547803000
C25	5.3910566664000	0.6959687678000	-1.4115324394000
H26	6.3859664371000	0.4917000025000	-1.0322636927000
C27	5.2149360921000	1.3909724711000	-2.6099344770000
H28	6.0777790684000	1.7365375243000	-3.1705118267000
C29	3.9242176353000	1.6175903078000	-3.0796886703000
H30	3.7070230438000	2.1265077779000	-4.0123220107000
C31	2.8532426386000	1.1499990040000	-2.3233406858000
C32	1.4649418458000	-1.1801262852000	2.7602413008000
C33	1.4244279111000	1.2334619106000	-2.7763101257000
C34	1.8648257848000	2.6627573787000	1.4254754655000
H35	2.7646047547000	2.1115082933000	1.6759181525000
C36	1.5904775364000	3.9058191592000	1.9820262323000
H37	2.3022810939000	4.3570862175000	2.6646026691000

C38	0.3774851600000	4.5298198769000	1.6841936965000
H39	0.0948968672000	5.4775084026000	2.1321391110000
C40	-0.5045952969000	3.9020964875000	0.8043993358000
C41	-0.1308490119000	2.6901385982000	0.2313591987000
H42	-0.7743489809000	2.1995517521000	-0.4783978567000
C43	-1.8681537032000	4.4819912181000	0.5561522767000
C44	-4.0076450068000	3.9884431667000	-0.3997884974000
H45	-4.0383023237000	4.9253207004000	-0.9642866218000
H46	-4.5151735761000	4.1390241775000	0.5580862929000
C47	-4.6297358428000	2.8585020425000	-1.1971388129000
H48	-5.6159367351000	3.1869261802000	-1.5595334568000
H49	-3.9896750432000	2.6217551947000	-2.0555746436000
C50	-5.1945335772000	0.5396662902000	-1.0614640074000
H51	-4.4405433370000	0.2443196692000	-1.8011666319000
H52	-6.1338616222000	0.7537191080000	-1.5938560753000
C53	-5.4680887425000	-0.6081284036000	-0.1011896472000
H54	-6.0570722626000	-0.2414384053000	0.7507540752000
H55	-6.0625009740000	-1.3611000812000	-0.6377768152000
C56	-4.4415554505000	-2.3473958259000	1.2423772024000
H57	-5.4989642070000	-2.4415937314000	1.5152791735000
H58	-3.8430666311000	-2.1639169450000	2.1384688969000
C59	-3.9804310134000	-3.6366732444000	0.5795804494000
H60	-4.4009181059000	-3.7655473440000	-0.4231015401000
H61	-4.2534007712000	-4.5033440714000	1.1916414976000
C62	-1.8988836989000	-4.3922031193000	-0.3380658016000
C63	-0.5062683359000	-3.8964039315000	-0.5983740530000
C64	0.2980889089000	-4.5266816696000	-1.5426401235000
H65	-0.0385265733000	-5.4575198367000	-1.9884016797000
C66	1.4941684197000	-3.9144484462000	-1.9281582483000
H67	2.1470107372000	-4.3682960288000	-2.6657487508000
C68	1.8104717925000	-2.6695763896000	-1.4048136286000
H69	2.6832159011000	-2.1170366619000	-1.7345444210000
C70	-0.0850056373000	-2.6886568370000	-0.0447152808000
H71	-0.6715030155000	-2.1953939528000	0.7244837356000
O72	-0.5702932071000	-0.0682112539000	0.0105239591000
O73	-1.7868547306000	-1.4529149072000	2.6971327730000
H74	-1.0525063074000	-1.7034488008000	3.2772993848000
H75	-1.6189683956000	-0.5183037955000	2.5084429011000
O76	-2.3244738845000	-1.7901934095000	-1.7786196480000
H77	-2.2803659510000	-0.9026915428000	-2.1711655584000
H78	-2.8597643001000	-1.6442283366000	-0.9840933762000
O79	-2.1935119160000	1.0425034079000	-2.0142635418000
H80	-2.2692920833000	0.9937515102000	-1.0460488508000
H81	-1.2430405864000	0.9262757942000	-2.1936180762000

O82	-2.5890002845000	0.9356980358000	1.1407259304000
H83	-3.3317853911000	1.5123696325000	0.8981942636000
H84	-2.9736653691000	0.0527703280000	0.9887701159000

Optimized transition state structure of catalyst 1 of Ru^{V} complex via WNA mechanism

E(B3LYP-D3/LACV3P**++) (a.u.) = -2605.630396

Solvation energy (kcal mol^{-1}) = -60.83

Zero-point energy (kcal mol^{-1}) = 405.224

ΔH_{298} (kcal mol^{-1}) = 31.099

ΔS_{298} (cal K^{-1} mol^{-1}) = 273.106

Cartesian coordinates

Ru1	0.9695011200000	0.1525245657000	-0.1836125051000
O2	-2.2792821373000	5.5013839923000	1.1819328811000
O3	-2.4910871762000	3.6783056900000	-0.1618216615000
O4	-4.2672585415000	1.3924575289000	-0.1901486777000
O5	-3.9258215658000	-1.3007663037000	0.7557207164000
O6	-2.3466521199000	-3.6868814632000	0.5674593525000
O7	-2.2971027896000	-5.4175356243000	-0.9094952378000
O8	0.6794802878000	0.8162437332000	-2.1146322827000
O9	1.4522767286000	1.9288612802000	-3.9033803857000
O10	0.4875197796000	-0.3928905775000	1.8080314403000
O11	0.7063693776000	-1.4363583978000	3.7638194696000
N12	1.0338862856000	2.1544096651000	0.4868245451000
N13	0.8765130875000	-1.8786374214000	-0.8150783232000
N14	2.9168384316000	-0.6053736484000	1.1688092797000
N15	3.0585547365000	0.4694716495000	-1.1223389314000
C16	2.6306565806000	-1.1598123058000	2.3434607012000
C17	3.6087506986000	-1.7597736916000	3.1338501208000
H18	3.3311536737000	-2.2001688122000	4.0848545661000
C19	4.9200618941000	-1.7580024758000	2.6523256444000
H20	5.7152591938000	-2.2189284679000	3.2300346828000
C21	5.2142434958000	-1.1548031574000	1.4261685481000
H22	6.2311593030000	-1.1429927588000	1.0497517525000
C23	4.1666685165000	-0.5758204038000	0.7006024191000
C24	4.2567204672000	0.1242854306000	-0.5974530195000
C25	5.4464092746000	0.4344683608000	-1.2588730834000
H26	6.3997220758000	0.1568958207000	-0.8241451735000
C27	5.3953199461000	1.1114198390000	-2.4782326167000
H28	6.3130327530000	1.3657841275000	-2.9992014586000
C29	4.1561488743000	1.4452546756000	-3.0173912954000
H30	4.0357804054000	1.9555071811000	-3.9663630669000
C31	3.0108097108000	1.0945582003000	-2.3059041445000
C32	1.1747447919000	-1.0159533661000	2.7010143963000
C33	1.6255721054000	1.3394384960000	-2.8505666672000
C34	1.8265114545000	2.6027175044000	1.4830358130000
H35	2.6586396306000	1.9645700255000	1.7569724927000
C36	1.5671742759000	3.7997066260000	2.1401969950000
H37	2.2371887629000	4.1390975657000	2.9225027877000

C38	0.3992812819000	4.5017147702000	1.8323113952000
H39	0.0976397920000	5.3852783446000	2.3864699179000
C40	-0.4223028259000	4.0167133631000	0.8157332577000
C41	-0.0261657135000	2.8821908294000	0.1134956730000
H42	-0.6073128370000	2.5018357081000	-0.7118921118000
C43	-1.8128188702000	4.5327925725000	0.6277226397000
C44	-3.9216648812000	3.7934321739000	-0.2523560885000
H45	-4.1871363708000	4.7067352985000	-0.7949151331000
H46	-4.3466288621000	3.8477691171000	0.7549159851000
C47	-4.3990007386000	2.5724423948000	-1.0057665353000
H48	-5.4596036630000	2.7157606322000	-1.2555249354000
H49	-3.8241177883000	2.4442062813000	-1.9310947232000
C50	-4.8714500438000	0.2536122500000	-0.8350177038000
H51	-4.2309082722000	-0.1036198683000	-1.6449734214000
H52	-5.8377468434000	0.5529663042000	-1.2642922441000
C53	-5.1428205804000	-0.8434521260000	0.1705733905000
H54	-5.8155275606000	-0.4744915855000	0.9594227151000
H55	-5.6527706959000	-1.6585034073000	-0.3645443860000
C56	-4.0953659572000	-2.4764459955000	1.5620425407000
H57	-5.1212616536000	-2.5329228240000	1.9497414483000
H58	-3.4064589325000	-2.3698721835000	2.4031286052000
C59	-3.7660750220000	-3.7478152250000	0.7923864785000
H60	-4.2777619432000	-3.7991400668000	-0.1739397174000
H61	-4.0165169447000	-4.6399029735000	1.3769009677000
C62	-1.7921620055000	-4.4456929919000	-0.3982082497000
C63	-0.4797847774000	-3.8501311874000	-0.8038202538000
C64	0.2762684178000	-4.3987359809000	-1.8351057298000
H65	-0.0120909082000	-5.3561609588000	-2.2573992030000
C66	1.3553858471000	-3.6651628051000	-2.3341441482000
H67	1.9678917238000	-4.0474463236000	-3.1433667769000
C68	1.6059575627000	-2.3964987205000	-1.8270674018000
H69	2.3830940873000	-1.7633265212000	-2.2370260485000
C70	-0.1186120437000	-2.6064621466000	-0.2909679886000
H71	-0.6776525230000	-2.1513367408000	0.5112007222000
O72	-0.8947387427000	0.0787599383000	-0.2387314681000
O73	-1.9521710255000	-0.6346762171000	3.0737373103000
H74	-1.0710249310000	-0.9312152321000	3.4029559716000
H75	-2.4062589929000	-0.2288974891000	3.8226948922000
O76	-2.6060789308000	-1.9475043651000	-1.8315914925000
H77	-2.4247408376000	-1.1304054447000	-2.3278862255000
H78	-2.7322772313000	-1.6539741753000	-0.9171699171000
O79	-2.1548861638000	0.8139225992000	-2.6435311833000
H80	-1.6425734912000	0.7326785966000	-1.8219835093000
H81	-1.4636606662000	0.9916804093000	-3.2977513860000

O82	-1.7999232330000	0.8973789249000	0.9844757364000
H83	-2.6929566957000	0.9646954256000	0.5338128729000
H84	-1.8392442719000	0.2092705350000	1.7514647807000
